# Supplementary material for: FOXO1 links KRAS G12D and G12V alleles to glutamine and nitrogen metabolism in colorectal cancer
Source: EMBO Rep. 2025 Nov 20;27(1):142–62. doi: 10.1038/s44319-025-00641-z (PMC12795846; doi:10.1038/s44319-025-00641-z)
Supplement: Supplementary file 1 — Appendix [file 44319_2025_641_MOESM1_ESM.pdf]

Appendix for

FOXO1 links KRAS G12D and G12V alleles to glutamine and nitrogen metabolism in colorectal cancer

Table of Contents

Appendix Table S1 2
Appendix Table S2 2
Appendix Table S3 2
Appendix Figure S1 3
Appendix Figure S2 5
Appendix Figure S3 6
Appendix Figure S4 8
Appendix Figure S5 10
Appendix Figure S6 11
Appendix Figure S7 13
Matlab code 14
Venn diagram 15
Enrichment analysis (Fig. 1 and Appendix Table S1) 15
Enrichment analysis (Fig. 4, Appendix Fig. S3 and Appendix Table S1) 16
References 17

Appendix Table S1

Genes differentially regulated in	Number of genes	Jupyter files	Link
SW48 G12A	851	1	EnrichR
SW48 G12C	482	2	EnrichR
SW48 G12D	1229	3	EnrichR
SW48 G12V	558	4	EnrichR
at least one mutant cell line (**Fig. 1A**)	1,935	5	EnrichR
all mutant cell lines with similar trends (**Fig. S1F** left)	97	6	EnrichR
in G12D and G12V with similar trends (**Fig. S1F** right)	334	7	EnrichR
orange cluster in **Fig. S1F** right	113	8	EnrichR

Appendix Table S1. Differentially regulated gene sets in SW48 cell lines. Every gene set was analysed using thresholds for false discovery rates (FDR) of 5% and a minimum 2-fold change. Links to gene enrichment analysis carried out with *EnrichR*[81] are embedded in the table. Data, matlab scripts, and Jupyter files are available in source data of **Appendix Figure S1F-J.**

Appendix Table S2

Id	Cell lines	Drugs	Media	Type	Figures
1	SW48 (WT, G12A/C/D/V)	-	Full media	RNAseq	1A-C and S1D-G, 2E
2	SW48 (WT, G12A/C/D/V)	-	Low nutrient	RNAseq	4C
3	SW48 (WT, G12D and V)	DMSO and iFOXO1	Low nutrient	RNAseq	4D, S3C
4	SW48 (WT, G12A/C/D/V)	-	Low nutrient	^{13}C-glucose LC-MS	2A-B, S2A-B, S2D, S2F
5	SW48 (WT, G12D and V)	DMSO and iFOXO1	Low nutrient	^{13}C-glucose LC-MS	3F, S3B
6	SW48 (WT, G12D and V)	-	Low nutrient	^{15}N-ammonia, ^{15}N-alpha-glutamine and ^{15}N-amide-glutamine LC-MS	4A-B, S4A-C
7	SW48 (WT, G12D and V)	DMSO and iFOXO1	Low nutrient	^{15}N-ammonia LC-MS	4E

Appendix Table S2. List of transcriptomics and metabolomics experiments. List of transcriptomics and metabolomics experiments with conditions and figures where the data is used. Data analysis and matlabl scripts used to generate figures are provided in source data files. Raw data is available on the Gene Expression Omnibus (GSE306286) and Metabolomics Workbench (ST004144) repositories.

Appendix Table S3

Genes differentially regulated in	Number of genes	Jupyter file	Link
SW48 Parental	4779	1	EnrichR
SW48 G12D	5221	2	EnrichR
SW48 G12V	5375	3	EnrichR
All	3058	4	EnrichR

Appendix Table S3. AS1842856 downregulates FOXO1 gene targets. List of transcripts downregulated (FDR<10% and log2 fold change less than -0.2) upon treatment with AS1842856. Gene enrichment analysis performed with EnrichR confirms that this small molecule inhibitor targets FOXO1 at the used concentration of 1 µM. Links to gene enrichment analysis carried out with *EnrichR*[81] are embedded in the table, data and scripts are available in the data source files of Figure 4 (EnrichR subfolder).

Appendix Figure S1

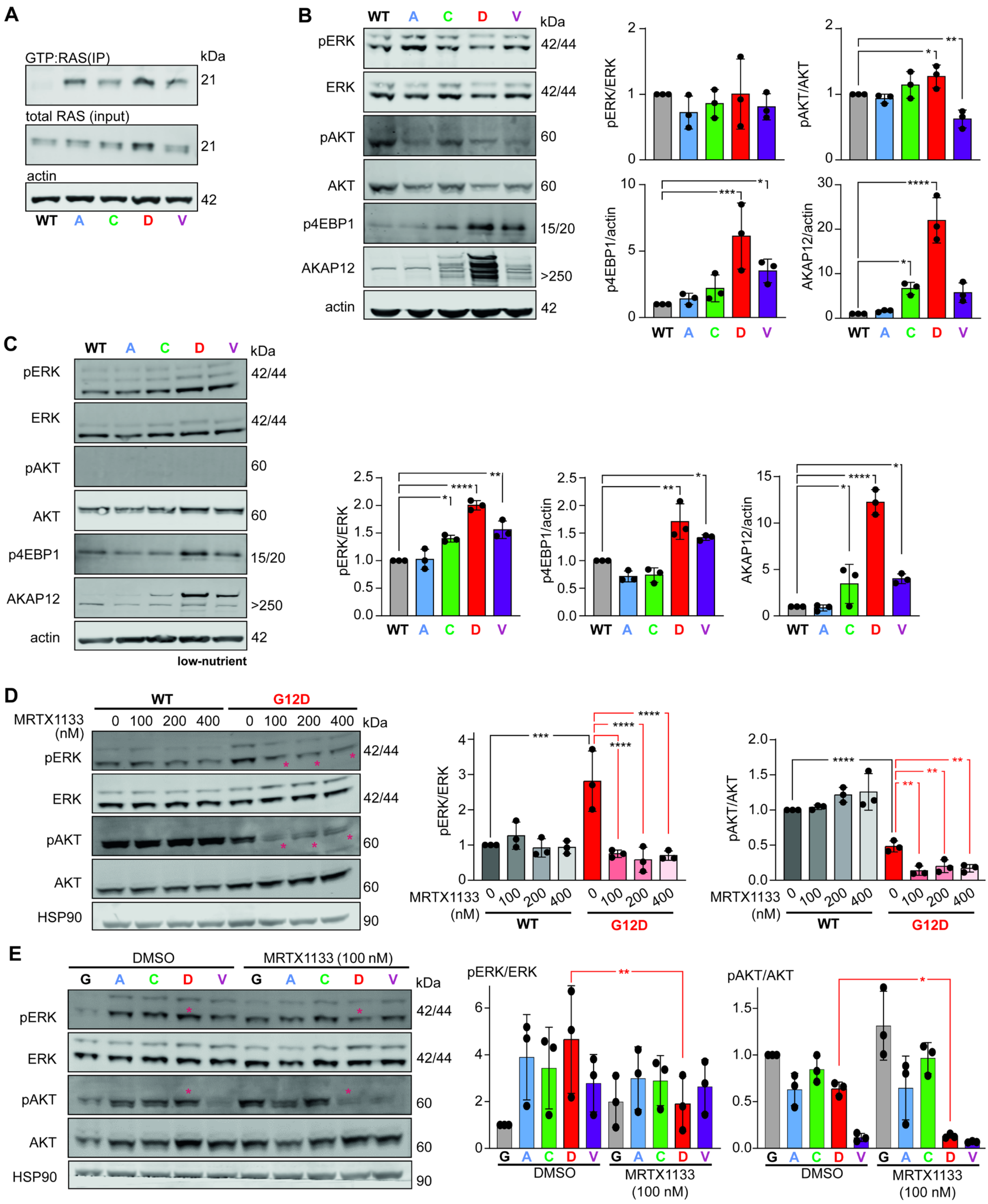

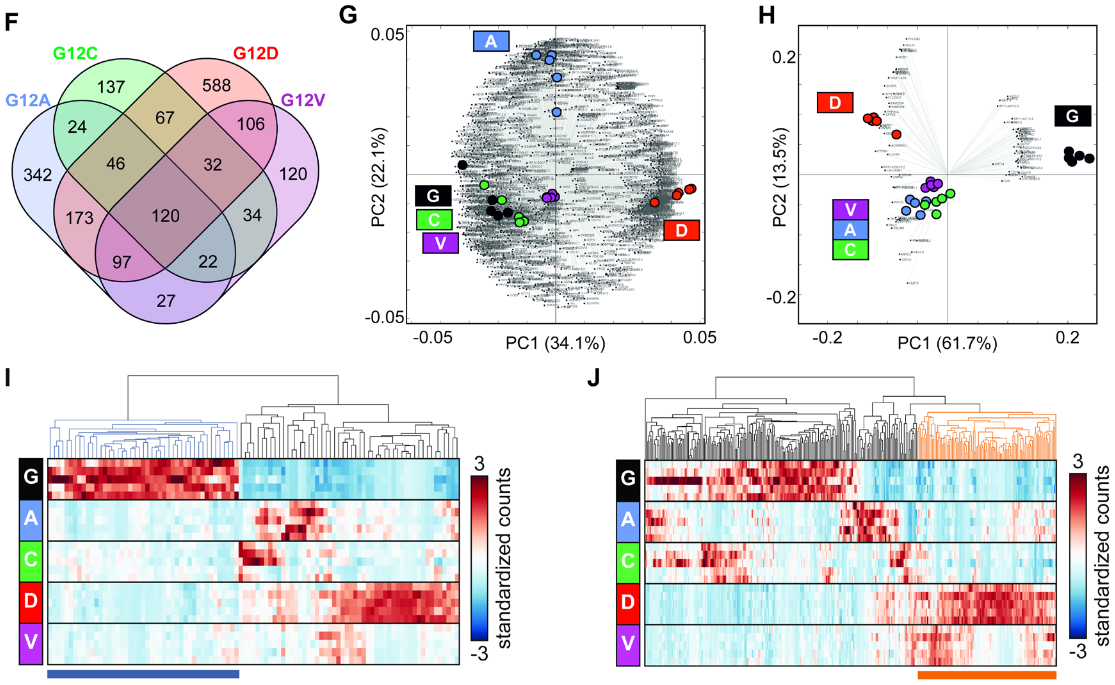

Appendix Figure S1 (related to Figure 1). Characterisation of the SW48 isogenic panel

(A) Immunoprecipitation of GTP-bound KRAS blotted tor RAS and actin. Lysates were prepared from SW48 cultured in full media.

(B-C) Western blots show differences in downstream signalling amongst the different cell lines of the SW48 isogenic panel cultured in full media (10% FCS and 11 mM glucose) (B) or in low nutrient media (1% FCS and 2 mM glucose) conditions (C). Graphs are means ± standard deviations of three biological replicates. Statistical analyses were performed using one-way ANOVA followed by Dunnett's multiple comparisons test comparing all mutants to the WT. Only statistically significant p values are shown (B- AKTs *p=0.015, **p=0.0022; p4EBP1 ***p=0.0008, *p=0.047; AKAP12 *p=0.05, **** $p \leq 0.0001$) (C- ERKs *p=0.013, **** $p \leq 0.0001$, **p=0.0018; p4EBP1 **p=0.003, *p=0.049; AKAP12 *p=0.03, **** $p \leq 0.0001$, *p=0.011).

(D) Validation of the SW48$^{+/G12D}$ cell line with the selective KRAS G12D inhibitor MRTX1133. Already at 100 nM MRTX1133 decreases the phosphorylation of AKT and ERK in SW48$^{+/G12D}$ but not in the parental line. This experiment was performed in full media with all samples controlled with the same concentration of DMSO. Means ± standard deviations of three biological replicates. Statistical analyses were performed using one-way ANOVA followed by Dunnett's multiple comparisons test for all comparisons. Only statistically significant p values are shown (ERKs ***p=0.0002, **** $p \leq 0.0001$; AKTs **** $p \leq 0.0001$, **p=0.0011, **p=0.0060, **p=0.0028).

(E) Effects of the selective KRAS G12D inhibitor MRTX1133 on the SW48 isogenic panel. This experiment was performed in full media with all samples controlled with the same concentration of DMSO. Other markers from the same lysate are shown in **Fig. 3C**. Graphs are means ± standard deviations of three biological replicates. Statistical analyses were performed using one-way ANOVA followed by Dunnett's multiple comparisons test for all comparisons. Only statistically significant p values are shown (ERKs **p=0.0024; AKTs *p=0.028).

(F) Venn diagram related to **Figure 1A** showing the number of genes that are differentially expressed in each mutant (n=5, FDR<5%, fold change > 2). For example, in these stringent statistical conditions, only 120 genes are differentially expressed in all mutants and G12D cells are those with the largest number (588) of genes to be uniquely regulated relative to WT.

(G) Biplot related to **Figure 1A**. Panels show the results of PCA analysis with the gene loading in the background, and the sample scores coloured in the foreground. The code, data and vectorial files useful to inspect other genes are listed in **Appendix Table S1**.

(H) Biplots related to the clustering analysis reported in panel I. The code, data and vectorial files useful to inspect other genes are listed in **Appendix Table S1**.

(I-J) The same analysis reported in **Figure 1A** but all genes are constrained in exhibiting similar trends of up- or down- regulation relative to wild-type (I). All cell lines exhibit downregulation of common genes (blue cluster). Panel J shows a similar analysis but for those genes that are similarly up- or down- regulated in the G12D and G12V mutant lines. The orange cluster highlights genes that are particularly upregulated in G12D with a significant overlap in G12V.

Appendix Figure S2

Appendix Figure S2 (related to Figure 2). Carbon flux from ^{13}C-labelled glucose in SW48 cells in low nutrient conditions.

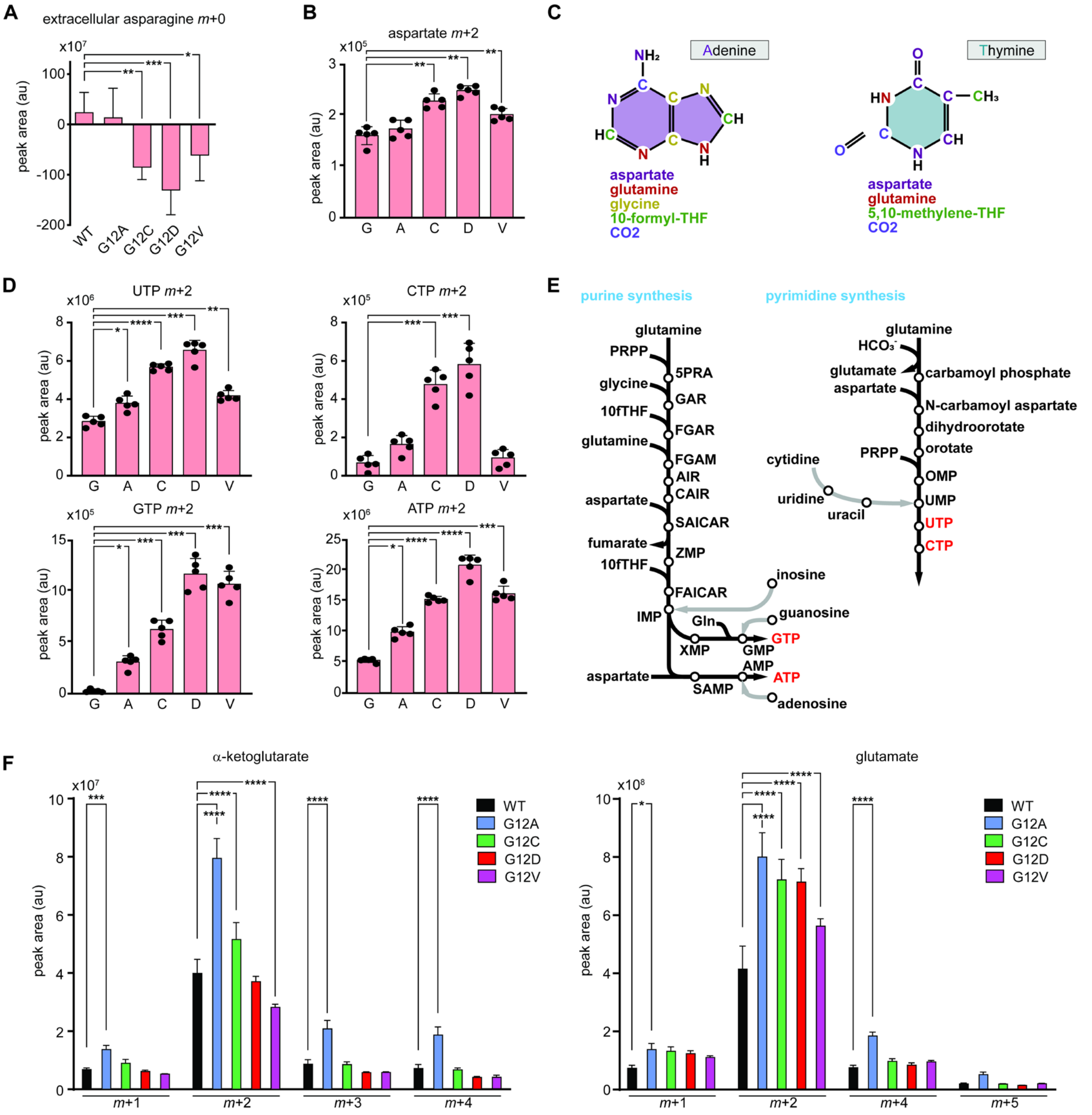

(A) Metabolic flux analysis using ^{13}C-labelled glucose shows increased asparagine uptake in KRAS G12C, G12D and G12V mutants determined by consumption-release measurements. Data are shown as means ± standard deviations of 5 technical replicates. Statistical analyses were performed using one-way ANOVA followed by Dunnett's multiple comparisons test comparing all mutants to WT. Only statistically significant p values are shown (**p=0.0036, ***p=0.0001, *p=0.024).

(B) Metabolic flux analysis using ^{13}C-labelled glucose shows increased synthesis of aspartate (m+2) in KRAS G12C, G12D and G12V mutants. Data are shown as means ± standard deviations of 5 technical replicates. Statistical analyses were performed using one-way ANOVA followed by Dunnett's multiple comparisons test comparing all mutants to WT. Only statistically significant p values are shown (**p=0.0022, **p=0.0013, **p=0.005).

(C) Diagrammatic representations of adenine and thymine depicting the sources of each atom for purine and pyrimidine nucleotides.

(D) Metabolic flux analysis using ^{13}C-labelled glucose shows increased synthesis of ATP, CTP, GTP, UTP in KRAS G12C, G12D and G12V mutants. Data are shown as means ± standard deviations of 5 technical replicates. Statistical analyses were performed using one-way ANOVA followed by Dunnett's multiple comparisons test comparing all mutants to WT. Only statistically significant p values are shown (UTP m+2 *p=0.035, **** p ≤ 0.0001, ***p=0.001, **p=0.008; CTP m+2 G12C ***p=0.0002, G12D ***p=0.0006; GTP m+2 **p=0.0014, G12C ***p=0.0003, G12D ***p=0.0002, G12V ***p=0.0001; ATP m+2 **p=0.0015, **** p ≤ 0.0001, ***p=0.0001).

(E) Pathways showing the synthesis of purine (ATP, GTP) and pyrimidine (UTP, CTP) nucleotides.

(F) Metabolic flux analysis using ^{13}C-labelled glucose shows all detected isotopologues of α-ketoglutarate (left) and glutamate (right) in KRAS G12 mutants showing higher levels of all isotopologues in the G12A mutant cells. Data are shown as means ± standard deviations of 5 technical replicates. Statistical analyses were performed using two-way ANOVA followed by Dunnett's multiple comparisons test for all comparisons. Only statistically significant p values are shown (αKG ***p=0.0003, **** p ≤ 0.0001; glutamate *p=0.0281, **** p ≤ 0.0001).

Appendix Figure S3

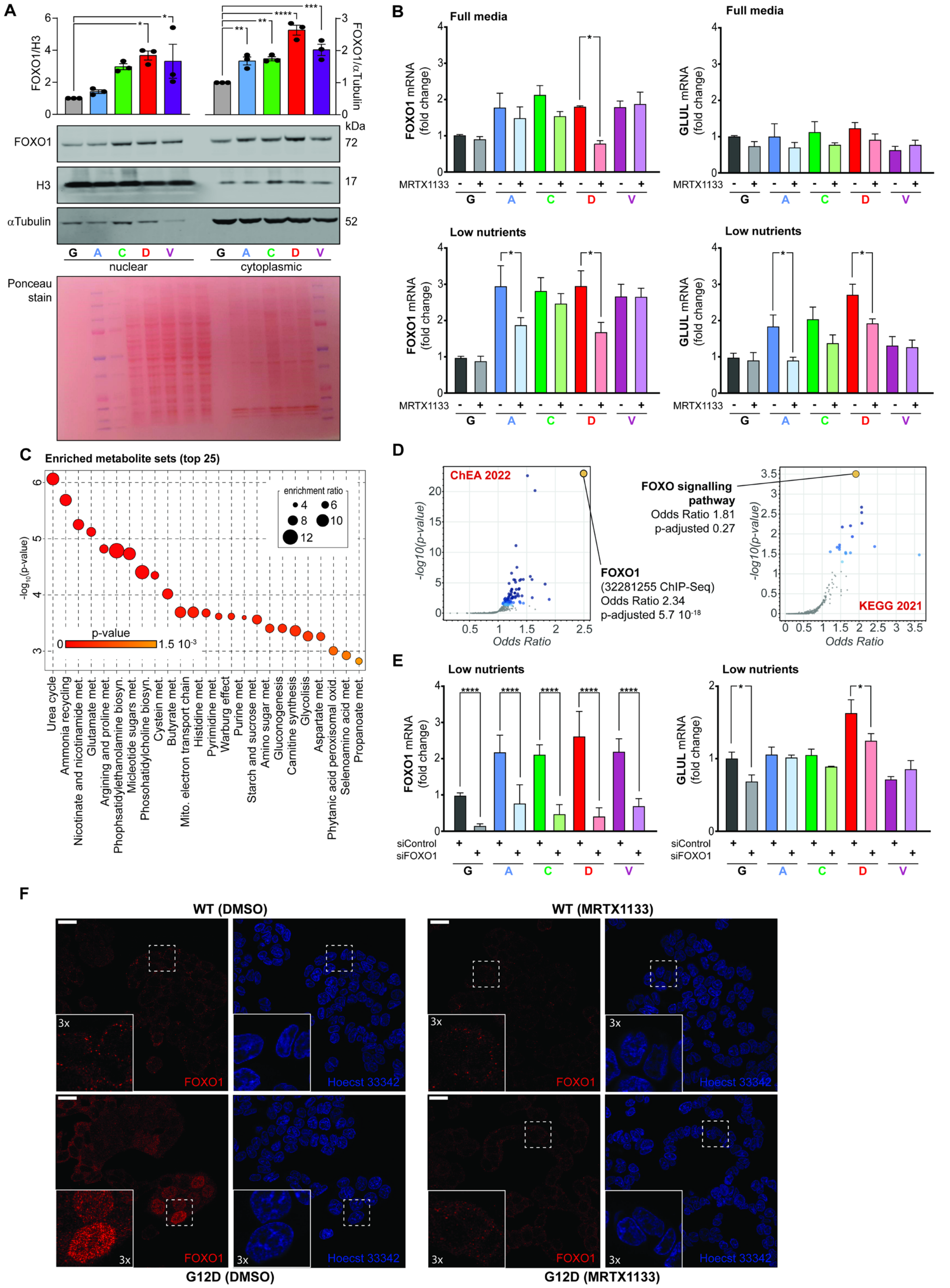

Appendix Figure S3 (related to Figure 3). FOXO1 nuclear and cytoplasmic fractions

(A) Representative immunoblot from nuclear-cytoplasmic fractionation experiments showing an increase in nuclear FOXO1 protein in KRAS mutants (full media). Graphs are means ± standard deviations of three biological replicates. Statistical analyses were performed using one-way ANOVA followed by Dunnett's multiple comparisons test comparing all mutants to the WT. Only statistically significant p values are shown (FOXO1 Cyto G12A **p=0.009, G12C **p=0.005, ****p≤0.0001, ***p=0.0005; FOXO1 Nuc G12D *p=0.012, G12V *p=0.027).

(B) qPCR analysis of *FOXO1* and *GLUL* (GS) mRNA expression relative to wild-type cells in full media (top row) and low-nutrient conditions (bottom row) in the presence or absence of MRTX1133 (100 nM). Graphs are means ± standard deviations of three biological replicates. Statistical analyses were performed using two-way ANOVA followed by Sidak's multiple comparisons test for all comparisons. Only statistically significant p values are shown (Full media FOXO1 *p=0.0263; Low nutrient FOXO1 G12A *p=0.046, G12D *p=0.015; GLUL G12A *p=0.013, G12D *p=0.045).

(C) Enrichment analysis of metabolites changed upon FOXO1 inhibition in ^{13}C-glucose carbon labelling experiment (low nutrient). The analysis was performed using Metaboanalyst 6.0[82].

(D) Volcano plots for all genes downregulated by FOXO1 inhibition (AS1842856 at 1 µM; low nutrient) in wild-type, G12D and G12V cell lines as measured by RNAseq. P-values and odd ratios for gene set enrichment were performed with EnrichR[81] (see **Appendix Table S3**); here, target genes of transcription factors (ChEA 2022) and KEGG pathways (KEGG 2021) are shown, both confirming that AS1842856 inhibits FOXO1.

(E) qPCR analysis of *FOXO1* and *GLUL* (GS) mRNA expression relative to wild-type cells in low-nutrient conditions upon FOXO1 knock-down. Graphs are means ± standard deviations of three biological replicates. Statistical analyses were performed using two-way ANOVA followed by Sidak's multiple comparisons test for all comparisons. Only statistically significant p values are shown (*FOXO1* ****p≤0.0001; *GLUL* WT *p=0.0415, G12D *p=0.0106).

(F) Immunostaining of SW48 cells (WT *vs* G12D) in full media in the presence of the MRTX1133 inhibitor or matched DMSO control shows the loss of FOXO1 nuclear expression upon inhibition of the KRAS G12D allele.

Appendix Figure S4

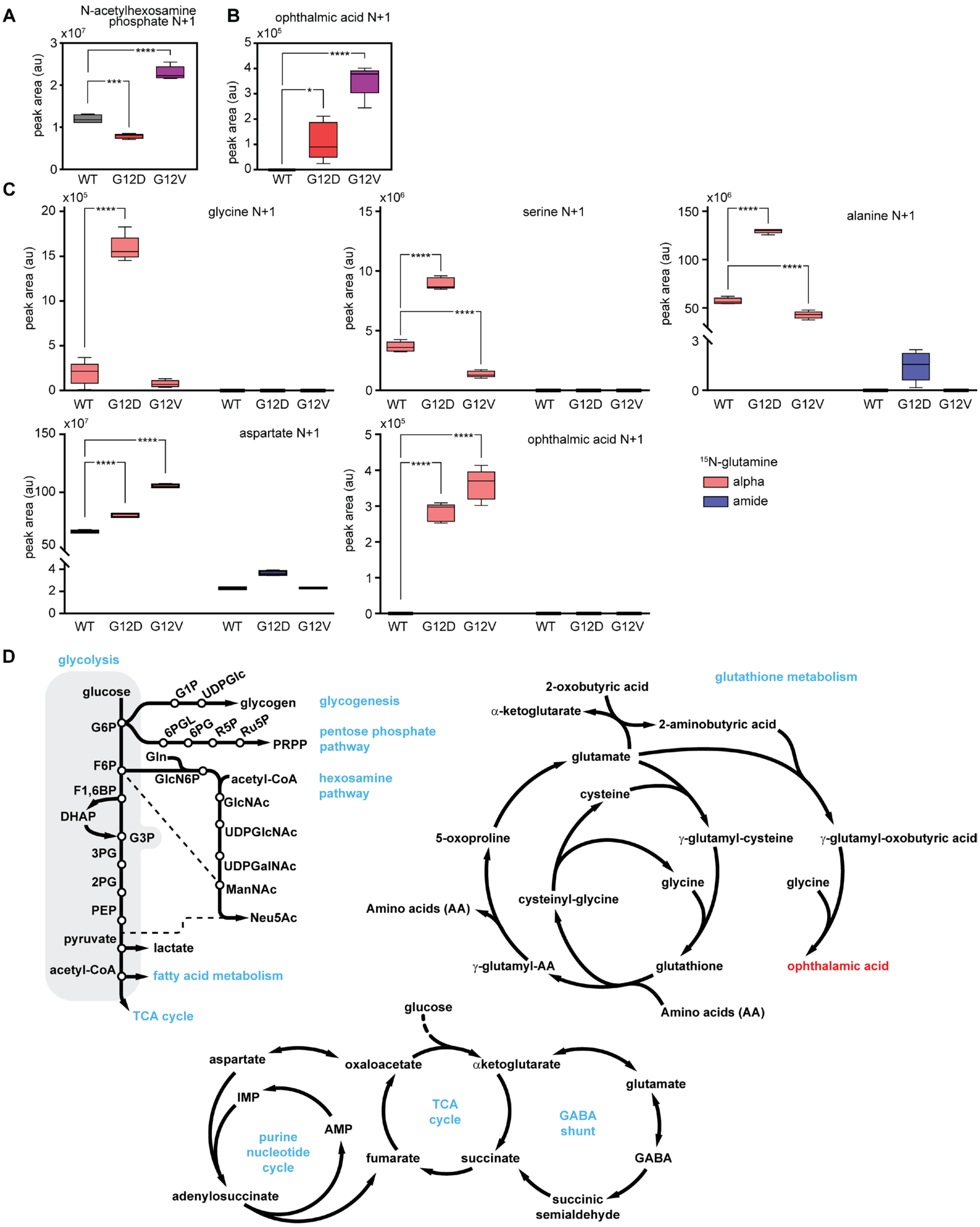

Appendix Figure S4 (related to Figure 4). Nitrogen metabolism in G12D and G12V SW48 mutant cells (low nutrient conditions).

(A) Nitrogen tracing using $^{15}NH_3$ shows that the synthesis of N-acetylhexosamine, a metabolite within the hexosamine pathway (see also panel D), is significantly upregulated in the SW48 G12V mutant line. Statistical analyses of 5 technical replicates were performed using one-way ANOVA followed by Dunnett's multiple comparisons test comparing all mutants to WT. Only statistically significant p values are shown (***p=0.0001, ****p≤0.0001). Box plots show the median (center line), interquartile range (box), and whiskers extending to the minimum and maximum values.

(B) Nitrogen tracing using $^{15}NH_3$ shows that the synthesis of ophthalmic acid, a metabolite produced by the same enzymes that synthesise glutathion (see also panel D), is significantly upregulated in the mutant cell lines, SW48 G12V in particular. Statistical analyses of 5 technical replicates were performed using one-way ANOVA followed by Dunnett's multiple comparisons test comparing all mutants to WT. Only statistically significant p values are shown (*p=0.014, ****p≤0.0001). Box plots show the median (center line), interquartile range (box), and whiskers extending to the minimum and maximum values.

(C) Nitrogen tracing with ^{15}N-amide-glutamine and ^{15}N-alpha-glutamine. The nitrogen at the glutamine alpha-carbon is incorporated into the backbone of several amino acids through transamination reactions that are particularly upregulated in G12D cells. Higher integration of nitrogen from glutamate into aspartate and ophthalmic acid is observed in both G12D and G12V mutants. Box plots show the median (center line), interquartile range (box), and whiskers extending to the minimum and maximum values.Statistical analyses of 5 technical repeats were performed using two-way ANOVA followed by Dunnett's multiple comparisons test for all comparisons. Only statistically significant p values are shown (****p≤0.0001).

(D) Diagrammatic representations of various pathways discussed in the paper.

Appendix Figure S5

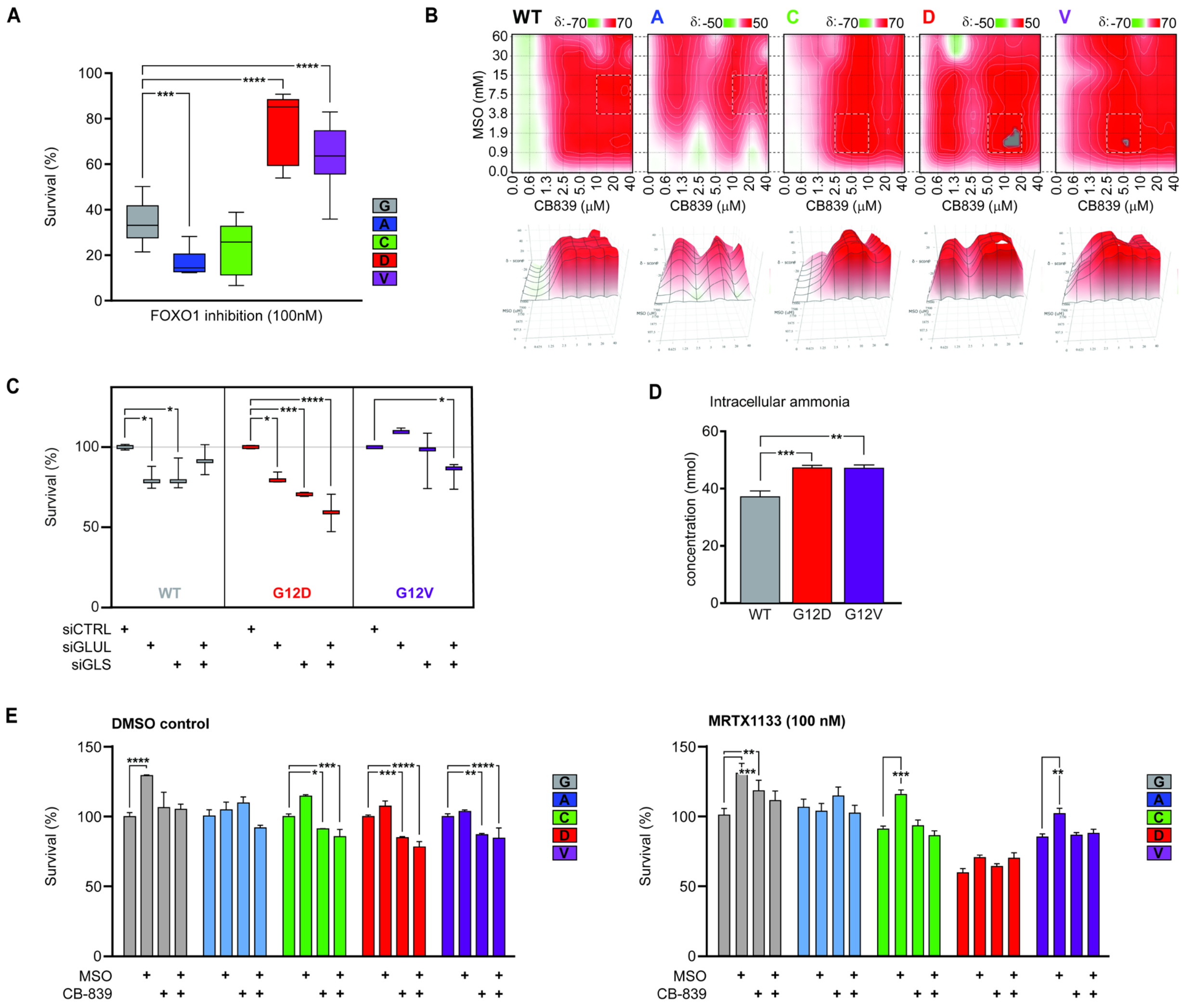

Appendix Figure S5 (related to Figure 5). Synergy analysis and validation (SW48 panel in low nutrient conditions)

(A) SRB viability assay of SW48 cells upon inhibition of FOXO1 (100 nM AS1842856), data normalised to DMSO control of each cell line. Box plots show the median (center line), interquartile range (box), and whiskers extending to the minimum and maximum values.Statistical analyses of 3 biological repeats were performed using one-way ANOVA followed by Dunnett's multiple comparisons test. Only statistically significant p values are shown (***p=0.0007, ****p≤0.0001).

(B) Synergistic effect of GS inhibitor, MSO, and GLS inhibitor, CB839, on KRAS mutant SW48 cells. BLISS analysis was performed with Synergy Finder[83].

(C) The combined knockdown of GLS and GLUL genes in SW48 cells sensitises G12D and G12V mutants. Box plots show the median (center line), interquartile range (box), and whiskers extending to the minimum and maximum values.Statistical analyses of 3 biological repeats were performed using two-way ANOVA followed by Dunnett's multiple comparisons test. Only statistically significant p values are shown (WT *p=0.017, *p=0.032; G12D *p=0.018, ***p=0.0004, ****p≤0.0001; G12V *p=0.047).

(D) Intracellular ammonia levels in SW48 cells were measured using an ammonia colourimetric assay. The graph shows means and standard deviations of 3 biological repeats. Statistical analyses were performed using one-way ANOVA followed by Dunnett's multiple comparisons test comparing all mutants to WT. Only statistically significant p values are shown (***p=0.0008, **p=0.0012).

(E) The effects of 100 nM MRTX1133 (right) or DMSO (left) on the response to MSO (2 mM) and CB839 (100 nM). Selective inhibition of KRAS G12D mutant protein abrogates SW48 sensitivity to MSO and CB839. Data are shown as means ± standard deviations of 3 biological repeats. Statistical analyses were performed using two-way ANOVA followed by Dunnett's multiple comparisons test. Only statistically significant p values are shown (DMSO WT ****p≤0.0001; G12C *p= 0.0321, ***p=0.0004; G12D ***p=0.0002, ****p≤0.0001; G12V **p=0.0011, ****p≤0.0001) (MRTX1133 WT ****p≤0.0001, **p=0.0037; G12C ***p=0.0001; G12V **p=0.005).

Appendix Figure S6

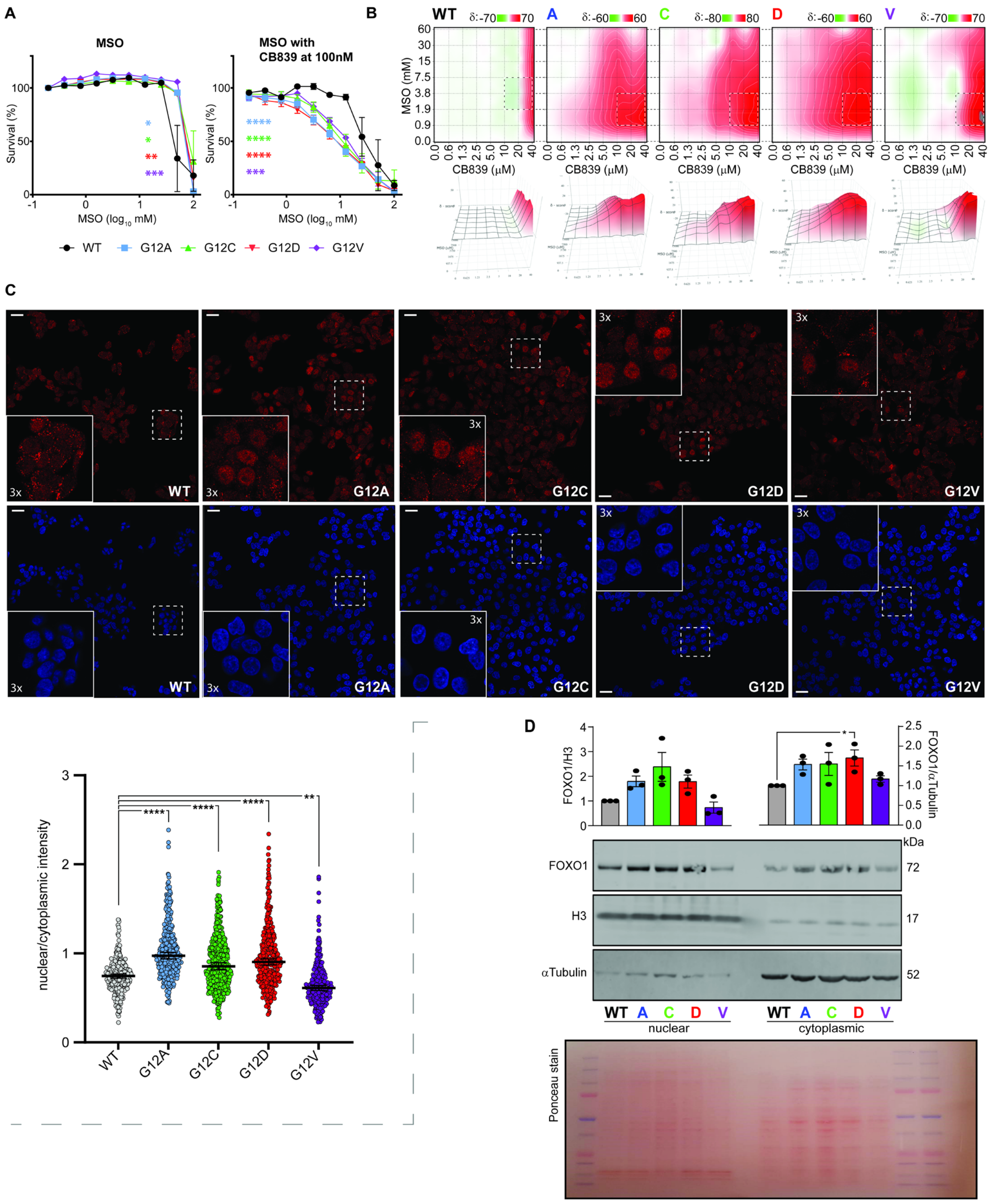

Appendix Figure S6 (related to Figure 5). Synergy analysis and validation (LIM1215)

(A) Viability curves showing titration of GS inhibitor, MSO, alone (left panel) or in combination with sub-lethal doses of GLS inhibitor, CB839, (right panel) in SW48 cells (low nutrient conditions). Data is presented as mean and standard error. Statistical analyses of Area Under the Curve (AUC) from 3 biological repeats were performed using one-way ANOVA followed by Sidak's multiple comparisons test for all comparisons. Only statistically significant p values are shown (left *p=0.0116, *p=0.0143, **p=0.0097,***p=0.0007) (right ****p≤0.0001, ***p=0.0001). **(B)** Synergistic effect of GS inhibitor, MSO, and GLS inhibitor, CB839, on KRAS mutant LIM1215 cells in low-nutrient conditions. BLISS analysis was performed with Synergy Finder[83].

(C) Immunostaining for FOXO1 (red) and Hoechst 3342 (blue) in LIM1215 cells (low-nutrient conditions). Scale bar: 25 µm; inserts: 3x magnification. The lower panel illustrates the quantification of nuclear/cytoplasmis intensity ratio from 3 biological repeats. Statistical analysis were performed using one-way ANOVA followed by Dunnett's multiple comparisons test. Only statistically significant p values are shown (****p≤0.0001, **p=0.0026).

(D) Representative immunoblots for FOXO1 labelling from nuclear-cytoplasmic fractionation experiments in LIM1215 cells (full media). Quantification shows means ± standard deviations of three biological replicates. Statistical analyses were performed using one-way ANOVA followed by Dunnett's multiple comparisons test comparing all mutants to the WT. Only statistically significant p values are shown (*p=0.036).

Appendix Figure S7

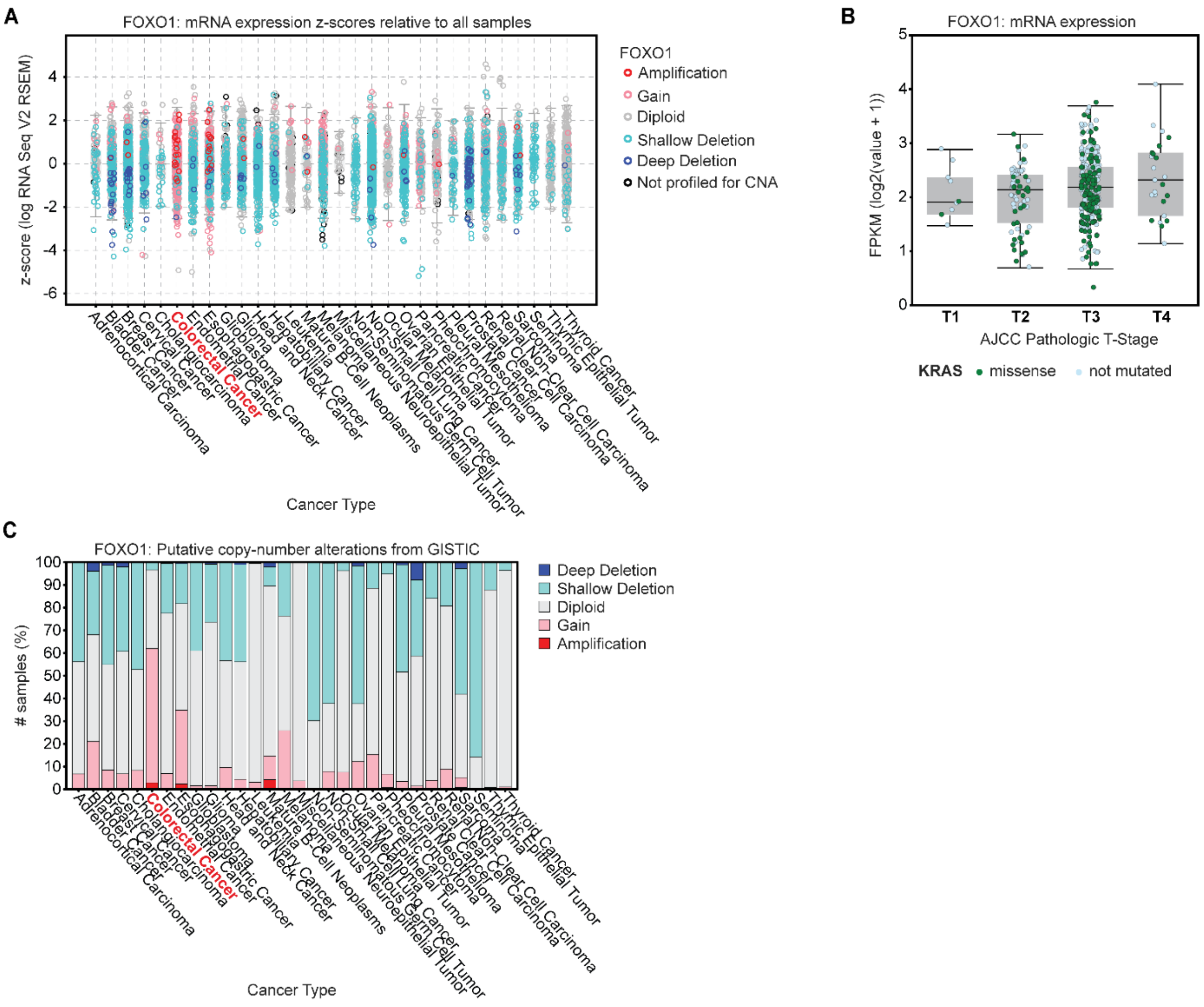

Appendix Figure S7. FOXO1 expression in cancer.

(A) FOXO1 mRNA expression across different cancer types. The graph was generated with cBioPortal.org using the TCGA PanCancer Atlas Studies. FOXO1 mRNA does not seem to correlate with cancer type. The colour of each measurement indicates copy number variations in the FOXO1 gene. Gains and amplifications seems to be particularly prevalent in colorectal cancers.

(B) FOXO1 mRNA expression in colorectal adenocarcinoma as a function of the American Joint Committee on Cancer (AJCC) tumour stage. The graph was generated with cBioPortal.org using the Colon Adenocarcinoma (TCGA, GDC) dataset. FOXO1 exhibits a mild upregulation during tumour progression. While exhibiting a strong correlation (R^2=0.95) with statistically different differences (one-way ANOVA, p~0.01), no statistical significance was identified between different groups or KRAS mutational status.

(C) The dataset shown in (A) was reanalysed for putative copy-number alterations, showing that the colorectal cancers exhibit the highest frequency for FOXO1 gene gain and amplifications across all cancers included in the TCGA PanCancer Atlas Studies.

Matlab code

The folder **MATLAB_KRAS_SW48** in the source data of **Appendix Figure S1F-J** stores Matlab code and results used to generate figures related to transcriptomics with **generate_data_for_figures_2024.m** script. All data generated by the Samarajiwa at Imperial College London (formerly at the MRC Cancer Unit, University of Cambridge, UK) was consolidated into a Matlab workspace **rnaseqdb2024.mat**.

plot_gene_expression.m codes for a function that plots the differential expression of genes, providing standard errors and adjusted p-values. The following Matlab command generates a bar plot for GLUL. Matlab figures were exported as EMF files and imported into Adobe Illustrator to generate panes at publication quality.

```
plot_gene_expression('GLUL',gene_names, fc, qv, se, cnt);
```

fig_process_2024.m codes for a function that can plot the following graphs:

- Hierarchical clustering of differentially expressed genes using gene counts
- Hierarchical clustering of differentially expressed genes using the differential expression contrasts relative to the wild-type cell line
- Vulcano plot showing genes that have significant fold changes and adjusted p-values (or FDR)
- Biplot showing how individual RNAseq experiment and sample clusters using Principal Component Analysis

The function should be called as follows:

```
fig_process_2024(cnt, qv, fc, lbl, gene_names, exp_def)
```

The variable that can be changed by the user is the structure defining the specific analysis (or experiment) that should be run (exp_def). This structure requires the following fields to be defined:

- **.trend** defines specific gene sets according to their upregulation or downregulation relative to wild-type. It supports four values:

  ‘+’ upregulated genes
  ‘-’ downregulated genes
  ‘’ (empty string) for any differentially regulated gene
  ‘=’ differentially regulated genes that exhibit the same trend across mutants

- **.fdr** defines the maximum accepted false discovery rate (or p-adjusted values) to create cohort of differentially regulated genes.

- **.foldchange** defines the minimum accepted fold change expression (in log2) to create cohort of differentially regulated genes.

- **.hits** defines the minimum number of mutant that show differences for a gene to be considered into a new gene set. For example, if analysing all four mutant cell lines, .hits=1 requires that a gene should be differentially regulated significantly in at least one mutant, while .hits=4 requires the gene to be differentially expressed in all mutants

- **.mutants** defines which mutant is analysed statistically. [1 2 3 4] or the array of constants [cMUT_A cMUT_C cMUT_D cMUT_V] requests the analysis of all mutants. However, if we want to plot only those genes altered in G12D, we would use .mutants=cMUT_D.

- **.name** simply define the folder name were to store all output files.

- **.kegg** defines a KEGG pathway (*e.g.*, hsa00250) that should be analysed. The code will restrict any analysis only on the gene sets defined by KEGG.

- **.highlightgenes** defines a list of genes that should be highlighted in the Biplots or Vulcano plots. For example .highlightgenes={‘FOXO1’,’GAD1’} will highlight the FOXO1 and GAD1 genes.

  - **.filetypes** defines which output the code will return, for example {{‘emf’,’png’}} will export both vectorial and raster images.

- **.destroy** is a Boolean flag the triggers the deletion of all figures if set to true. It is a useful option when multiple figures are generated with a single script. Data will be still accessible through the saved files.

- **.stats** is a Boolean flag, usually set to true, that activate statistical filtering that uses .fdr, .foldchange and .hits. If set to false, the script will not filter out any gene.

Licences: we are using cbrewer2 that can be downloaded from Matlab Exchange to implement Cynthia Brewer http://colorbrewer.org lookuptables.

The script **generate_data_for_figures_2024.m** contains several examples on how to use the code.

Venn diagram

The results used to plot the Venn diagram in **Appendix Fig. S1F** are available in source data files (sub-folder **FigS1F**). The Venn diagram was generated with the webtool https://bioinformatics.psb.ugent.be/webtools/Venn/ freely available at the webpage of the Bioinformatics & Evolutionary Genomics group, University of Gent, Belgium.

In the folder, a text file with the gene sets corresponding to the intersections shown below is available.

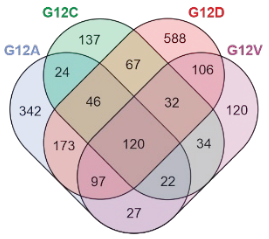

Enrichment analysis (Fig. 1 and Appendix Table S1)

Examples of data analysis utilized for **Figure 1** and described also in **Appendix Table S1** are available in the folder **AppendixTableS1**.

We provide the Jupyter notebooks generated with EnrichR. Link to the datasets stored on EnrichR are provided in **Appendix Table S1**. The data is one example of enrichment analysis using KEGG 2021 pathways. In this compressed folder we also include the comma separated files providing p- and q- values generated by EnrichR, and the plots related to the relevant clusters. The data is summarized in the figure below.

Each panel shows a volcano plot generated with EnrichR's Appyter included in the Jupyter notebooks and generated using the -log10(p-values) plotted versus the odds ratios. The blue circles represent the KEGG 2021 pathways that are identified as enriched of differentially regulated genes in G12A, G12C, G12D, and G12V, in at least one mutant cell line, and in all cell lines with similar trends (*i.e.*, upregulated or downregulated in each mutant cell line).

The last two plots shows the pathways enriched of genes differentially regulated with similar trends in G12D and G12V mutant cells, and those genes that are upregulated in these two mutants that represent the cluster shown in **Appendix Fig. S1J** with the orange bar.

The lists below the plot show the pathways identified by EnrichR as statistically significant. The results and methods are discussed in the main manuscript. These files are provided to ensure long-term access to the analyses in the case EnrichR will become unavailable in the future.

Enrichment analysis (Fig. 4, Appendix Fig. S3 and Appendix Table S1)

AS1842856 is a well-characterized inhibitor for FOXO1. To validate that AS1842856 inhibits FOXO1 at the sub-lethal concentration of 1 µM, we selected all genes downregulated upon FOXO1 treatment (log2<-0.2 and fdr<0.1). The lists of genes for SW48 parental, G12D and G12V were obtained by filtering the excel file "SW48_transcriptomics_iFOXO1.xlsx" found in **Fig. 4 data sources**. A Venn diagram was generated with the webtool https://bioinformatics.psb.ugent.be/webtools/Venn/ freely available at the webpage of the Bioinformatics & Evolutionary Genomics group, University of Gent, Belgium. In the folder, a text file with the gene sets corresponding to the intersections shown below is available.

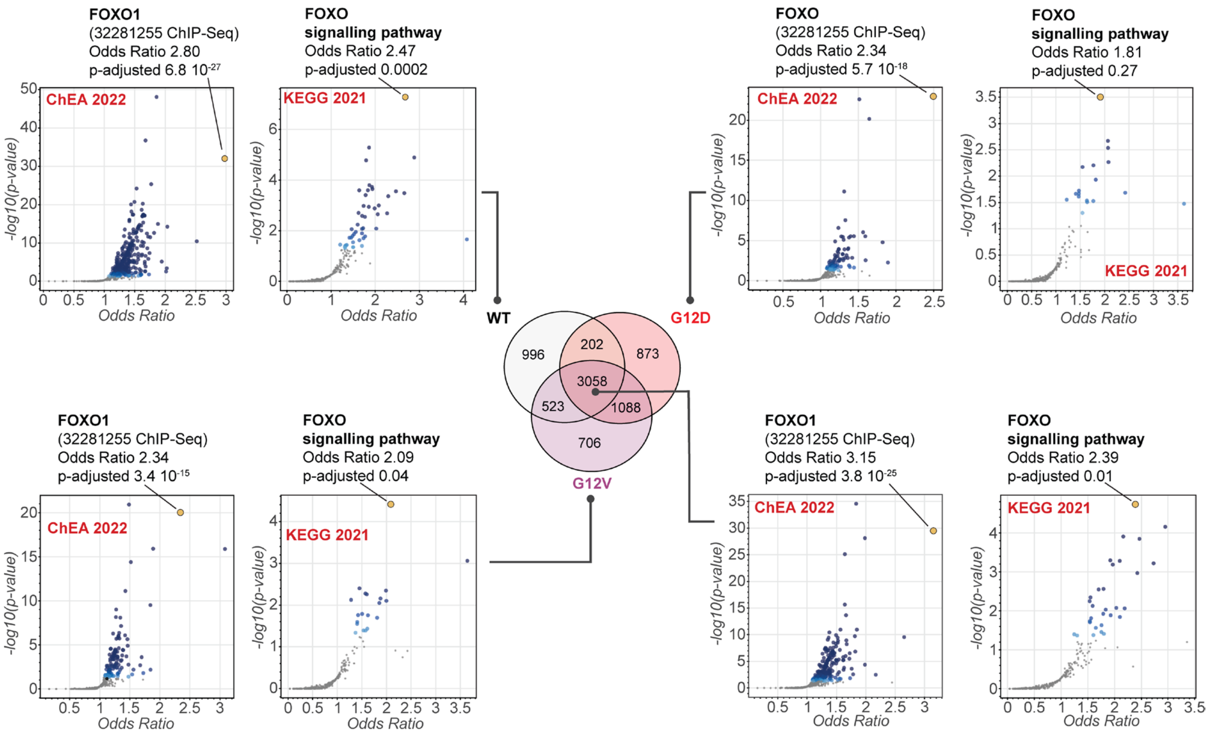

Data analysis utilized for **Appendix Figure S3** and described also in **Appendix Table S3**. We provide the Jupyter notebooks generated with EnrichR[1]. Link to the datasets stored on EnrichR are provided in **Appendix Table S3**. Each panel shows a volcano plot generated with EnrichR's Appyter included in the Jupyter notebooks and generated using the -log10(p-values) plotted versus the odds ratios. The blue circles represent either the KEGG 2021 pathways of the ChEA 2022 transcription factor target analysis that are identified as enriched of down-regulated genes upon treatment of cells with FOXO1 inhibitor.

This data was used to validate FOXO1 inhibition.

References

1. Chen, E.Y., Tan, C.M., Kou, Y., Duan, Q., Wang, Z., Meirelles, G.V., Clark, N.R., and Ma'ayan, A. (2013). Enrichr: interactive and collaborative HTML5 gene list enrichment analysis tool. BMC Bioinformatics 14, 128. https://doi.org/10.1186/1471-2105-14-128.
