# Supplementary figures and images for: FOXO1 links KRAS G12D and G12V alleles to glutamine and nitrogen metabolism in colorectal cancer
Source: EMBO Rep. 2025 Nov 20;27(1):142–62. doi: 10.1038/s44319-025-00641-z (PMC12795846; doi:10.1038/s44319-025-00641-z)

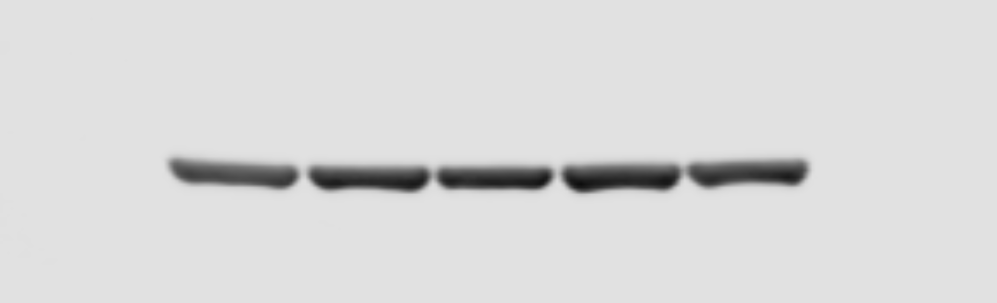

Supplement: Supplementary file 4 — Source data Fig. 2 [file 44319_2025_641_MOESM4_ESM.zip › Fig2/2F - Actin.png]

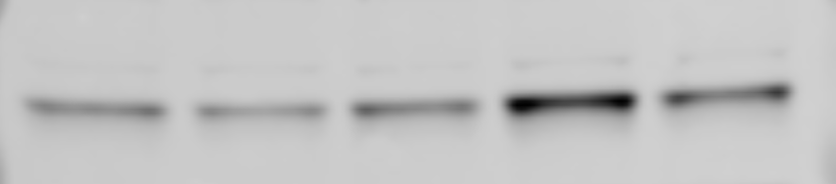

Supplement: Supplementary file 4 — Source data Fig. 2 [file 44319_2025_641_MOESM4_ESM.zip › Fig2/2F - GS cropped.png]

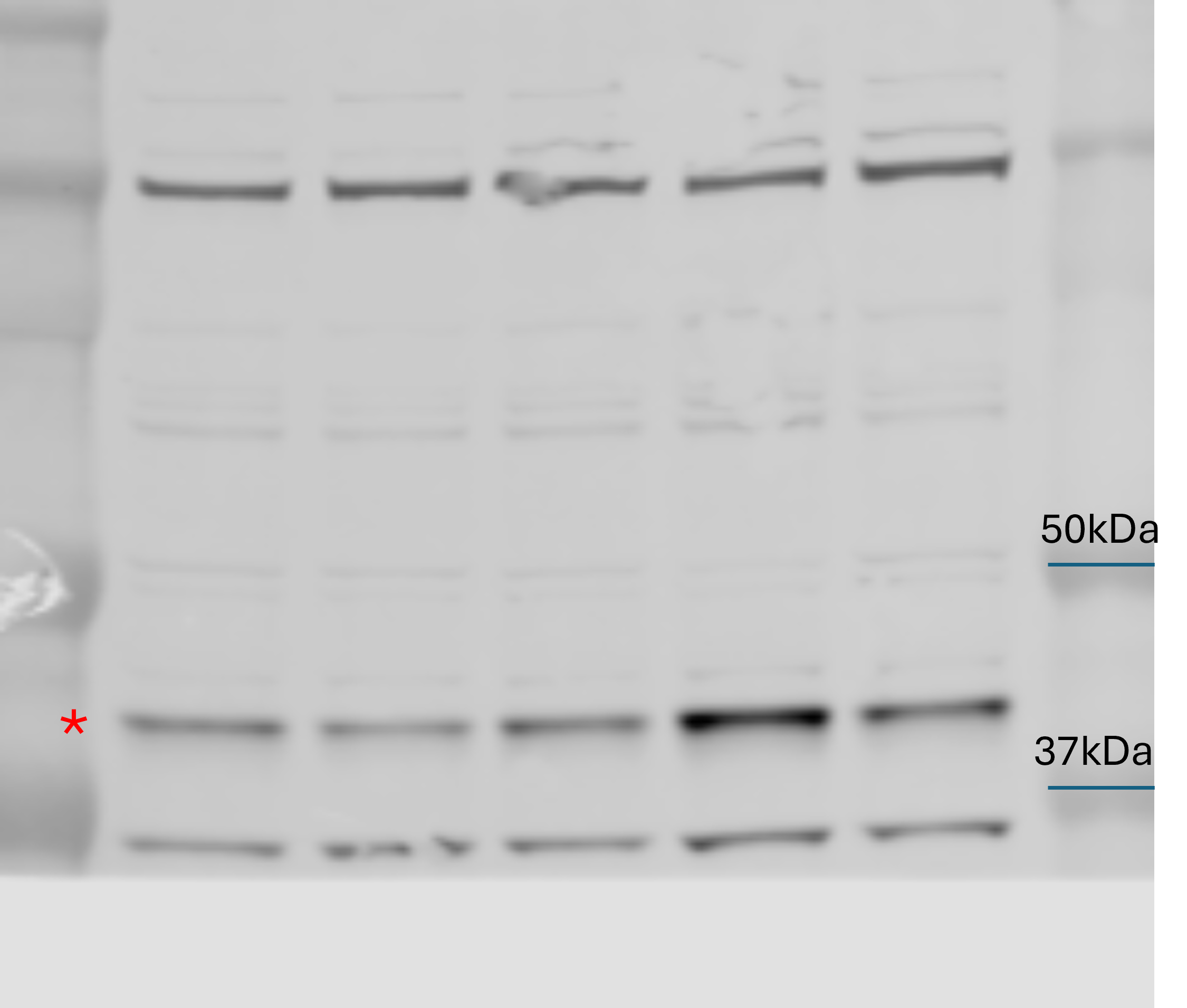

Supplement: Supplementary file 4 — Source data Fig. 2 [file 44319_2025_641_MOESM4_ESM.zip › Fig2/2F - GS labelled.png]

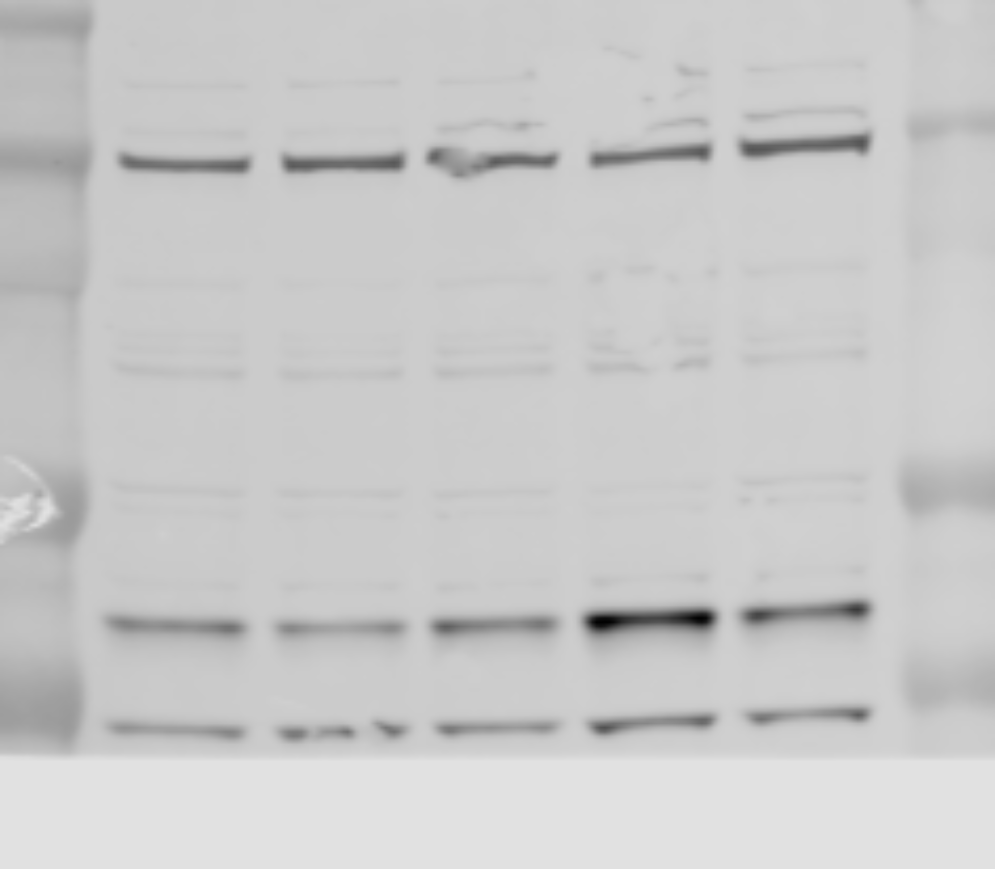

Supplement: Supplementary file 4 — Source data Fig. 2 [file 44319_2025_641_MOESM4_ESM.zip › Fig2/2F - GS.png]

# A.

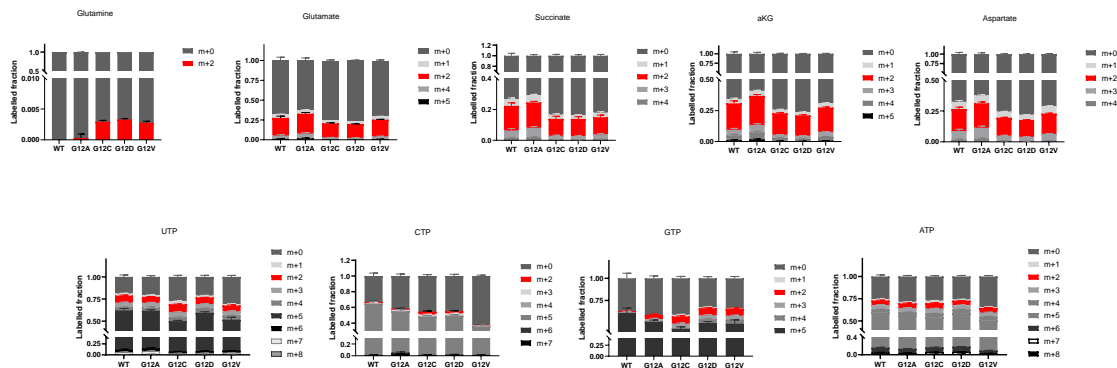

# B.

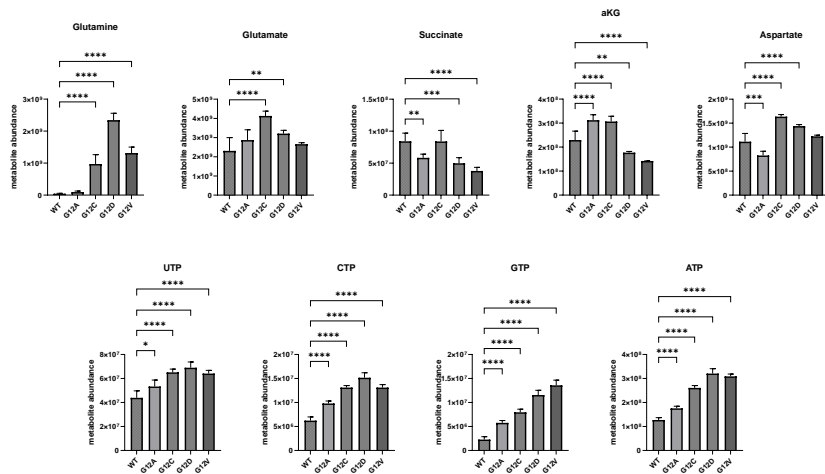

Supplement: Supplementary file 4 — Source data Fig. 2 [file 44319_2025_641_MOESM4_ESM.zip › Fig2/Metabolomics_13C_glucose_ALL/13C labelling_Fractions and total.pdf]

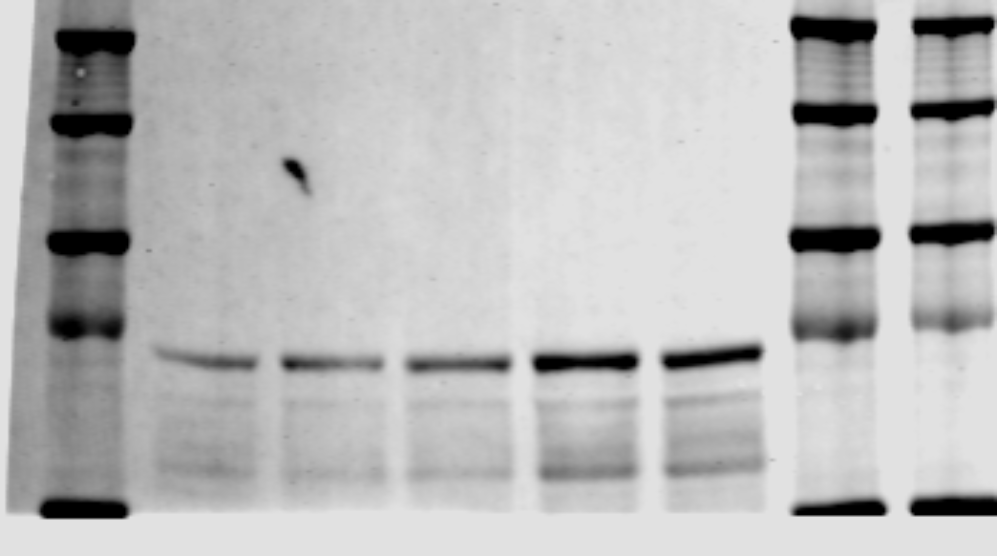

Supplement: Supplementary file 5 — Source data Fig. 3 [file 44319_2025_641_MOESM5_ESM.zip › Fig3/Fig3/3A/3A - Foxo1.png]

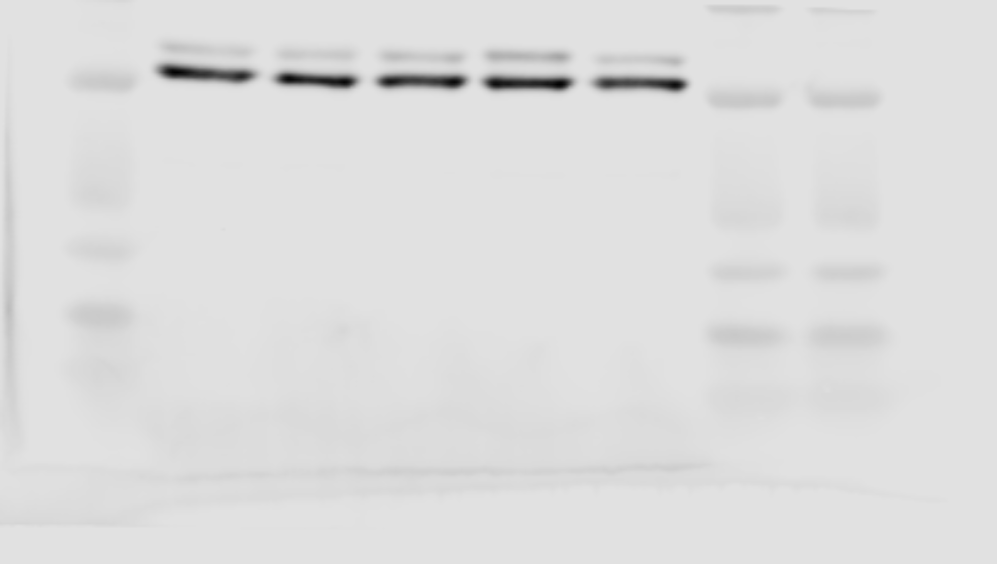

Supplement: Supplementary file 5 — Source data Fig. 3 [file 44319_2025_641_MOESM5_ESM.zip › Fig3/Fig3/3A/3A - totErk.png]

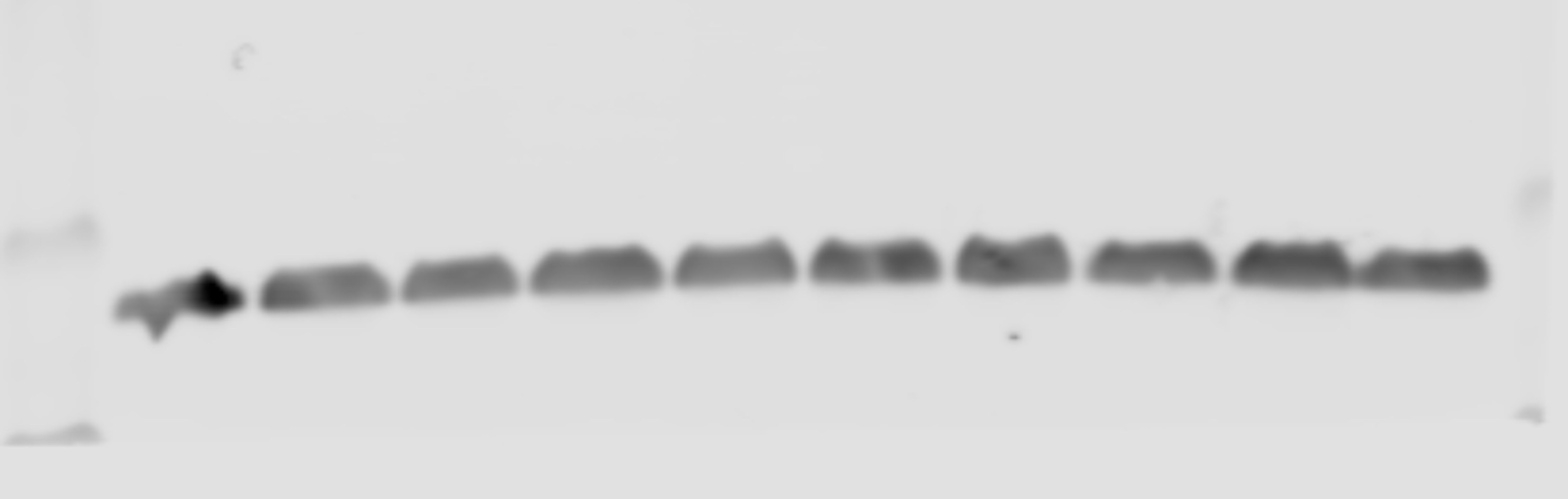

Supplement: Supplementary file 5 — Source data Fig. 3 [file 44319_2025_641_MOESM5_ESM.zip › Fig3/Fig3/3C/3C - Actin.png]

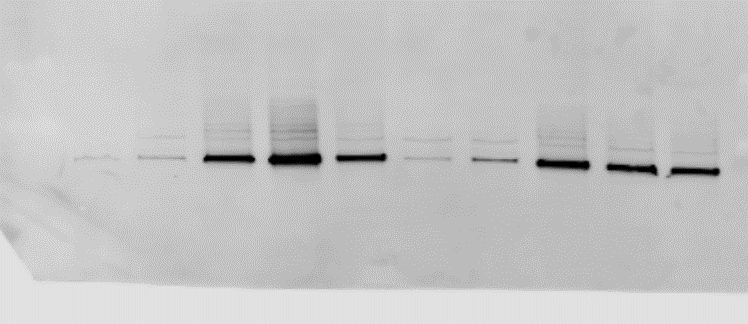

Supplement: Supplementary file 5 — Source data Fig. 3 [file 44319_2025_641_MOESM5_ESM.zip › Fig3/Fig3/3C/3C - Akap12.png]

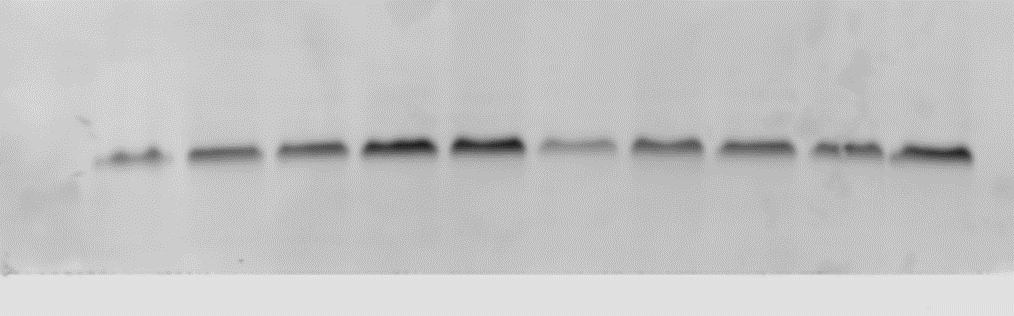

Supplement: Supplementary file 5 — Source data Fig. 3 [file 44319_2025_641_MOESM5_ESM.zip › Fig3/Fig3/3C/3C - Foxo1.png]

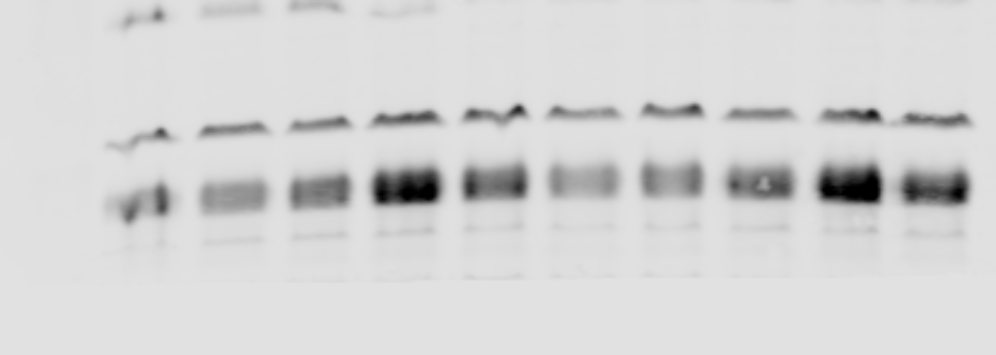

Supplement: Supplementary file 5 — Source data Fig. 3 [file 44319_2025_641_MOESM5_ESM.zip › Fig3/Fig3/3C/3C - GS.png]

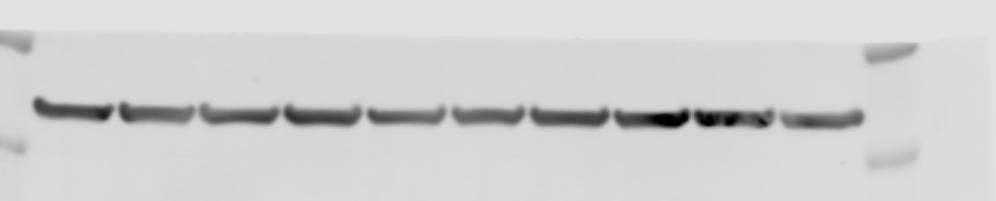

Supplement: Supplementary file 5 — Source data Fig. 3 [file 44319_2025_641_MOESM5_ESM.zip › Fig3/Fig3/3D/3D - Actin.png]

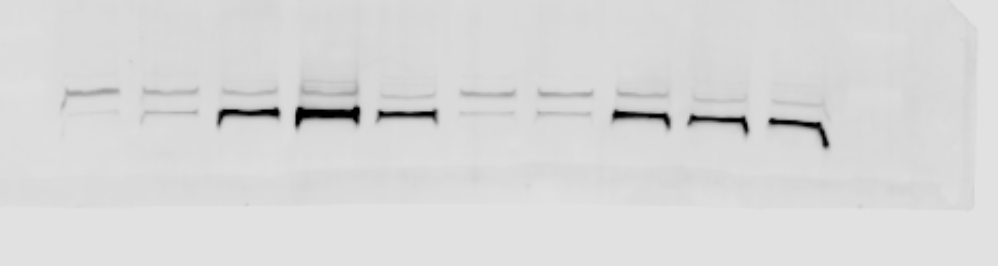

Supplement: Supplementary file 5 — Source data Fig. 3 [file 44319_2025_641_MOESM5_ESM.zip › Fig3/Fig3/3D/3D - Akap12.png]

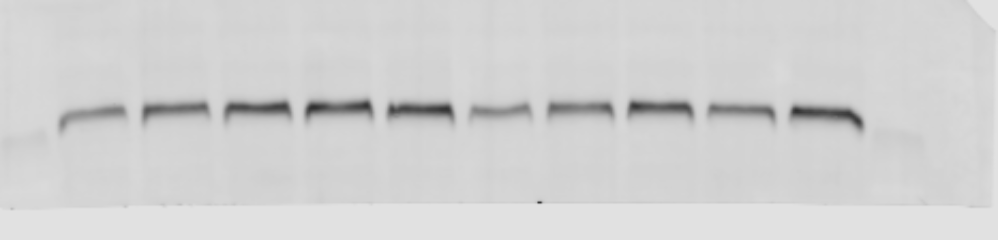

Supplement: Supplementary file 5 — Source data Fig. 3 [file 44319_2025_641_MOESM5_ESM.zip › Fig3/Fig3/3D/3D - Foxo1.png]

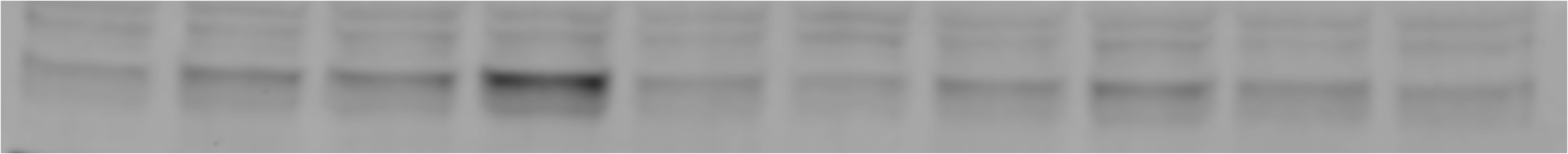

Supplement: Supplementary file 5 — Source data Fig. 3 [file 44319_2025_641_MOESM5_ESM.zip › Fig3/Fig3/3D/3D - GS cropped.png]

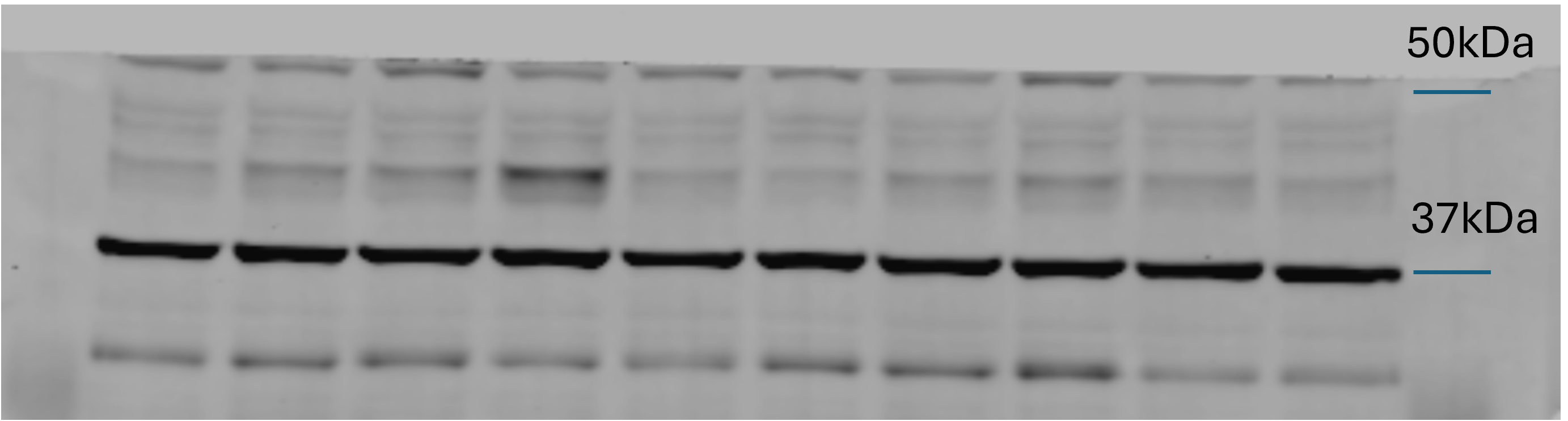

Supplement: Supplementary file 5 — Source data Fig. 3 [file 44319_2025_641_MOESM5_ESM.zip › Fig3/Fig3/3D/3D - GS labelled.png]

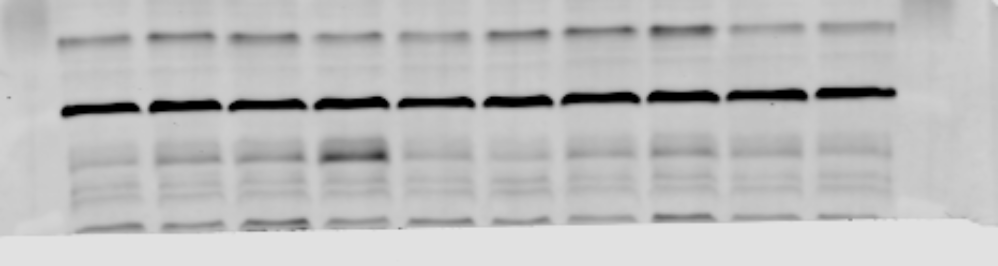

Supplement: Supplementary file 5 — Source data Fig. 3 [file 44319_2025_641_MOESM5_ESM.zip › Fig3/Fig3/3D/3D - GS.png]

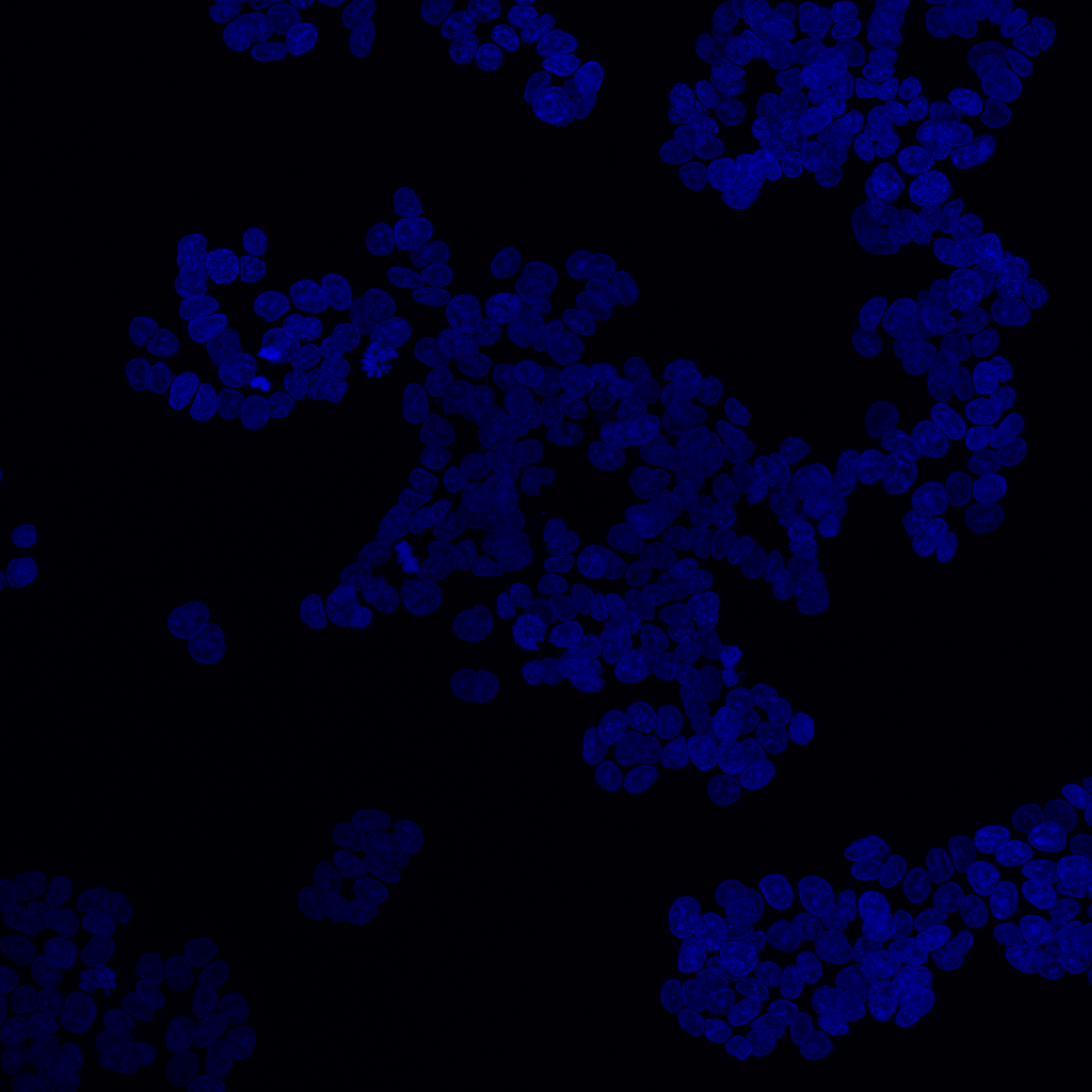

Supplement: Supplementary file 5 — Source data Fig. 3 [file 44319_2025_641_MOESM5_ESM.zip › Fig3/Fig3/3E/3E - DAPIs/G12Adapi.tif]

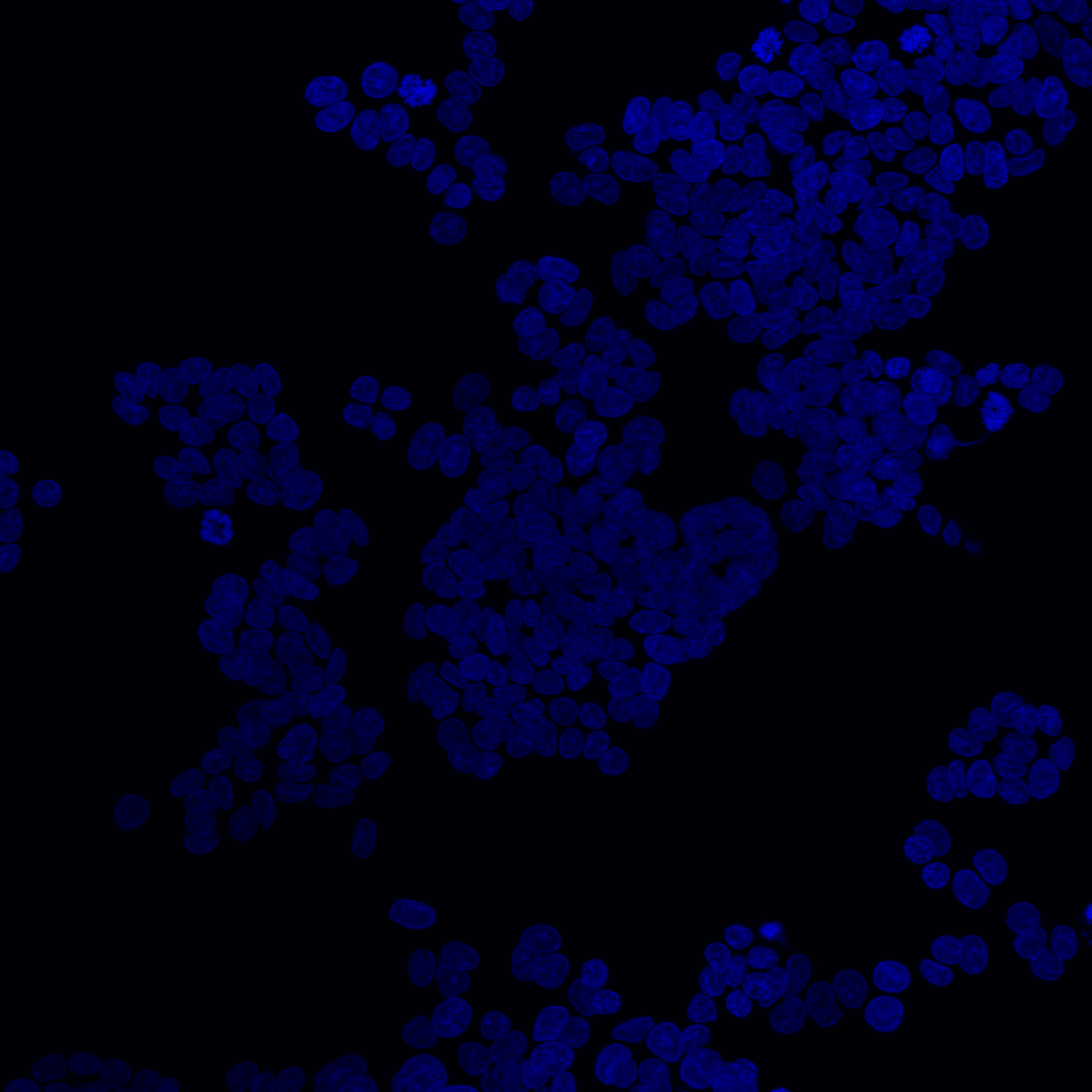

Supplement: Supplementary file 5 — Source data Fig. 3 [file 44319_2025_641_MOESM5_ESM.zip › Fig3/Fig3/3E/3E - DAPIs/G12Cdapi.tif]

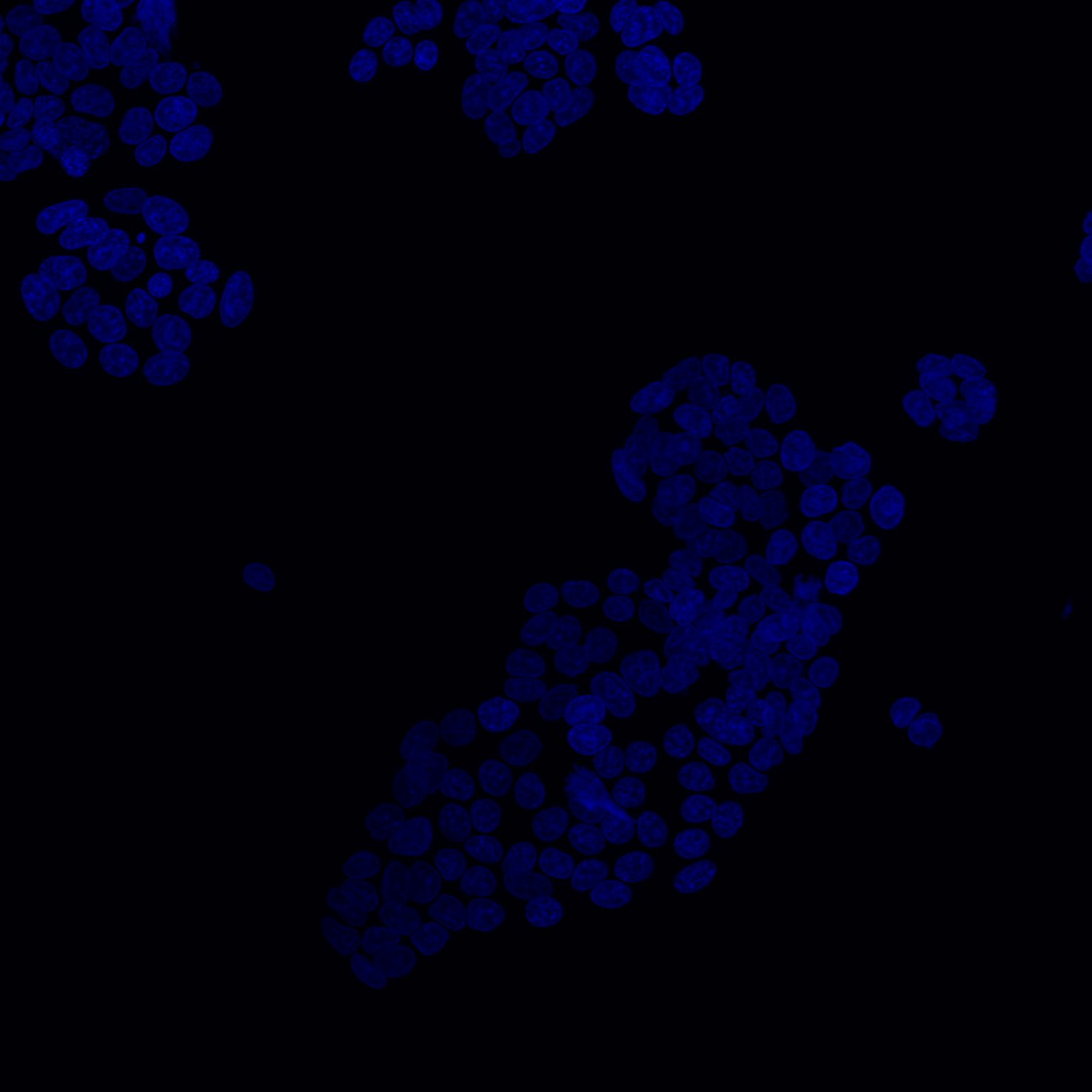

Supplement: Supplementary file 5 — Source data Fig. 3 [file 44319_2025_641_MOESM5_ESM.zip › Fig3/Fig3/3E/3E - DAPIs/G12Ddapi.tif]

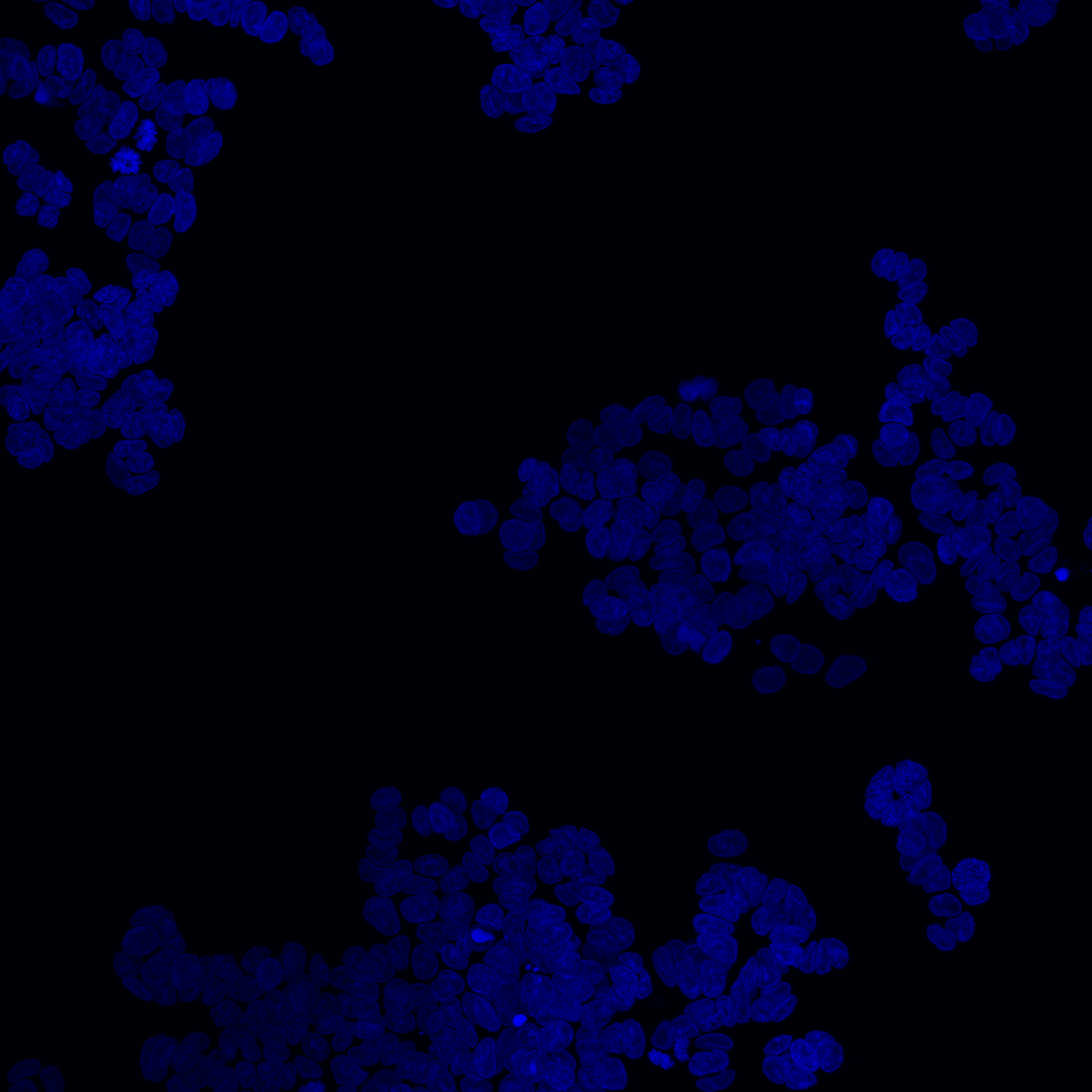

Supplement: Supplementary file 5 — Source data Fig. 3 [file 44319_2025_641_MOESM5_ESM.zip › Fig3/Fig3/3E/3E - DAPIs/G12Vdapi.tif]

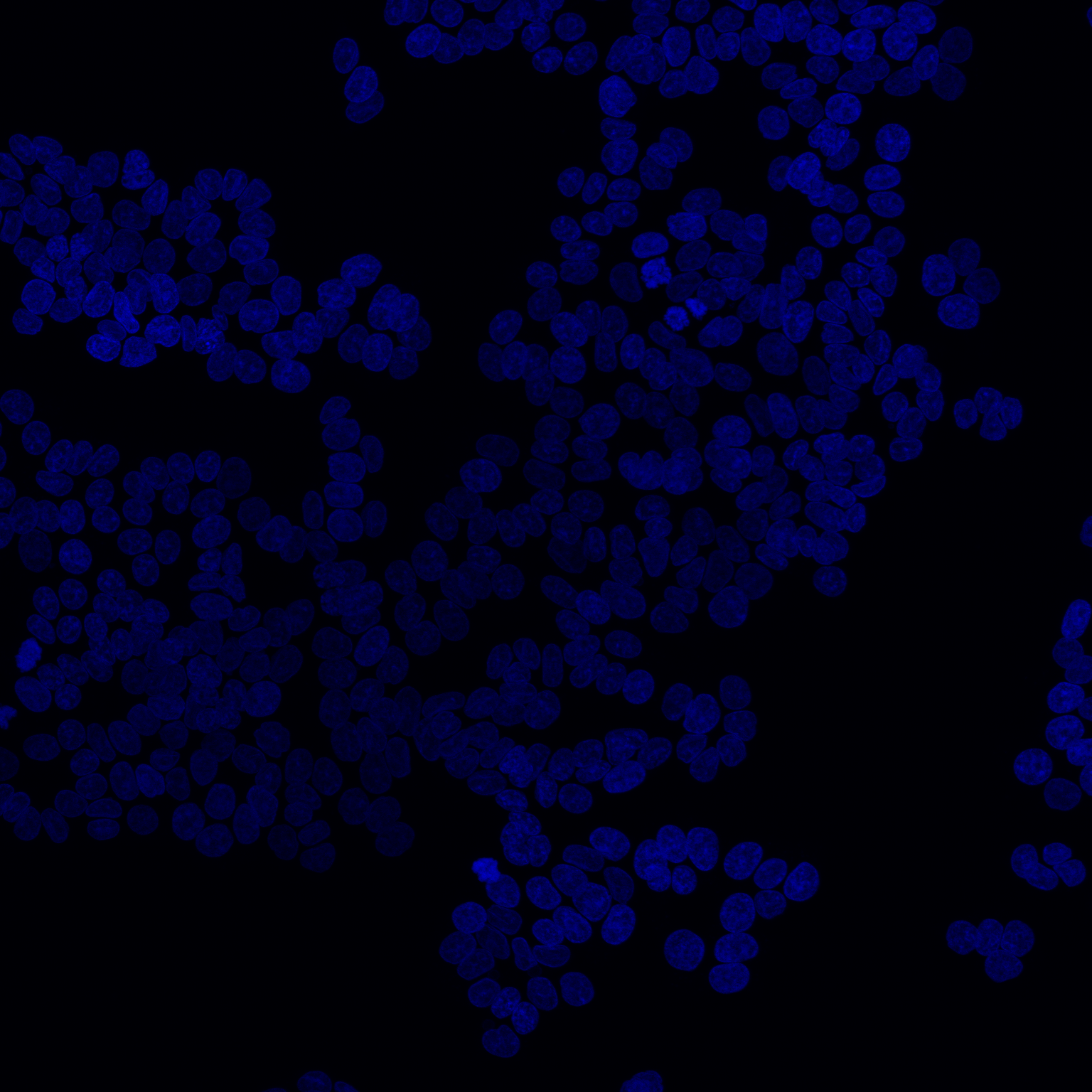

Supplement: Supplementary file 5 — Source data Fig. 3 [file 44319_2025_641_MOESM5_ESM.zip › Fig3/Fig3/3E/3E - DAPIs/WTdapi.tif]

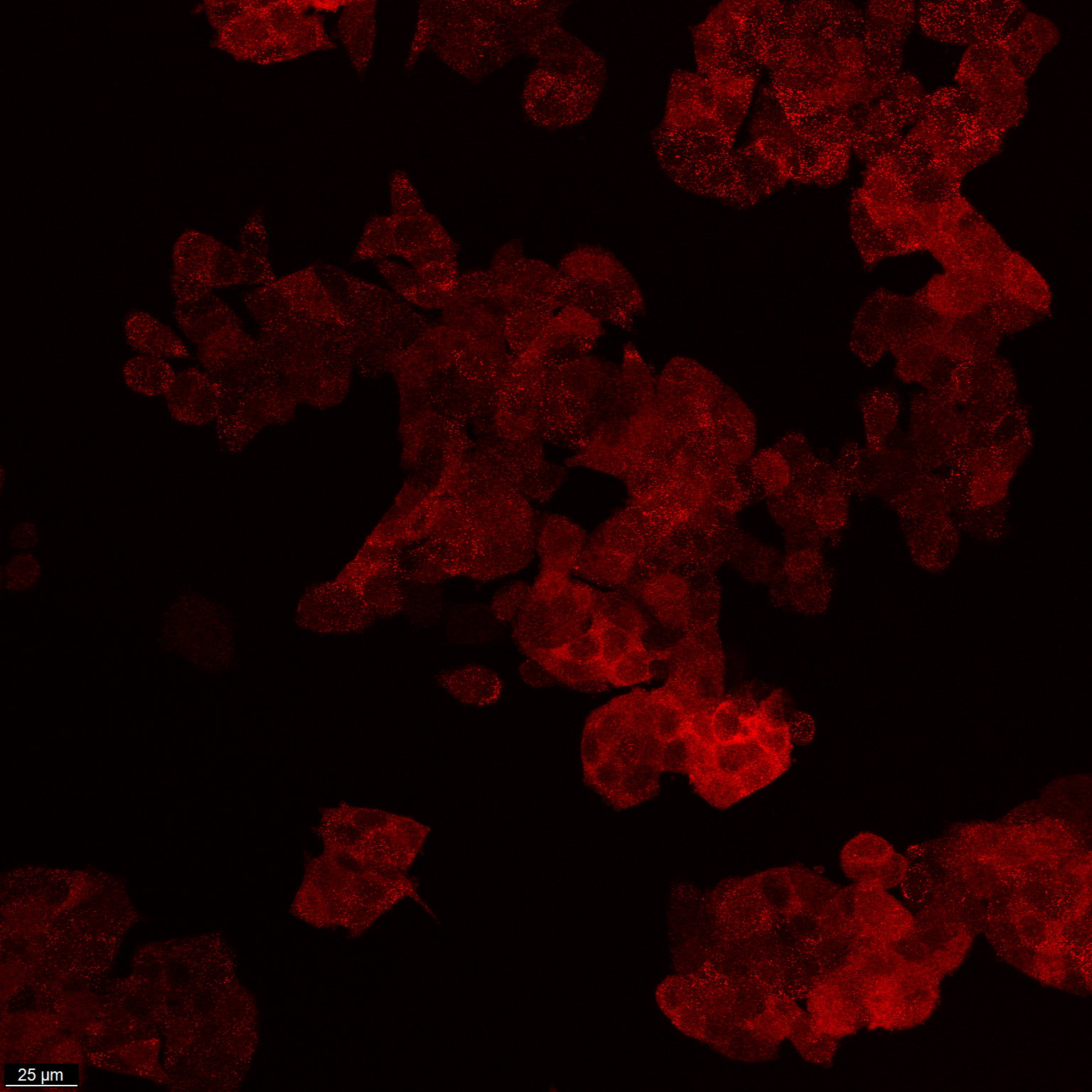

Supplement: Supplementary file 5 — Source data Fig. 3 [file 44319_2025_641_MOESM5_ESM.zip › Fig3/Fig3/3E/3E - FOXO1/G12A.tif]

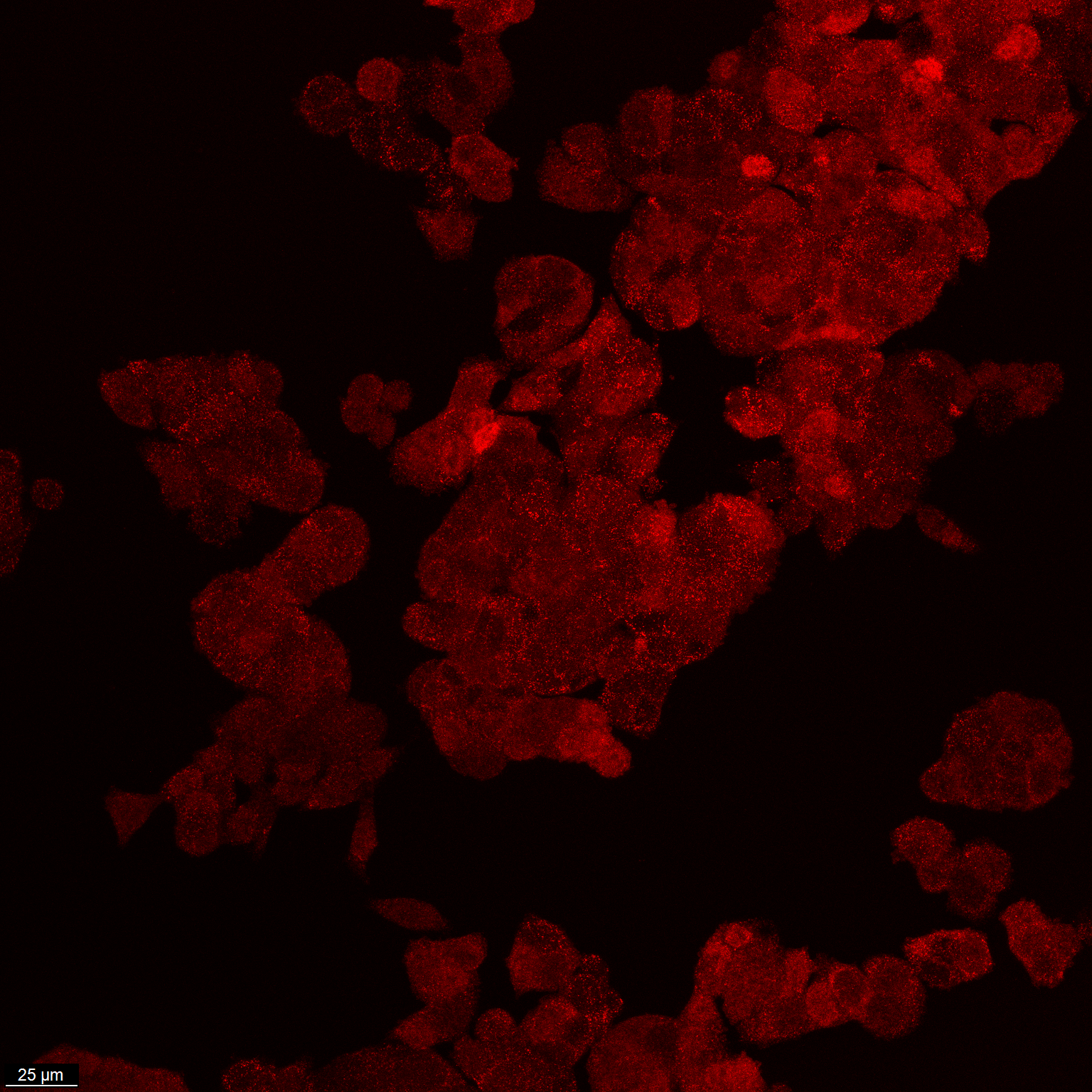

Supplement: Supplementary file 5 — Source data Fig. 3 [file 44319_2025_641_MOESM5_ESM.zip › Fig3/Fig3/3E/3E - FOXO1/G12C.tif]

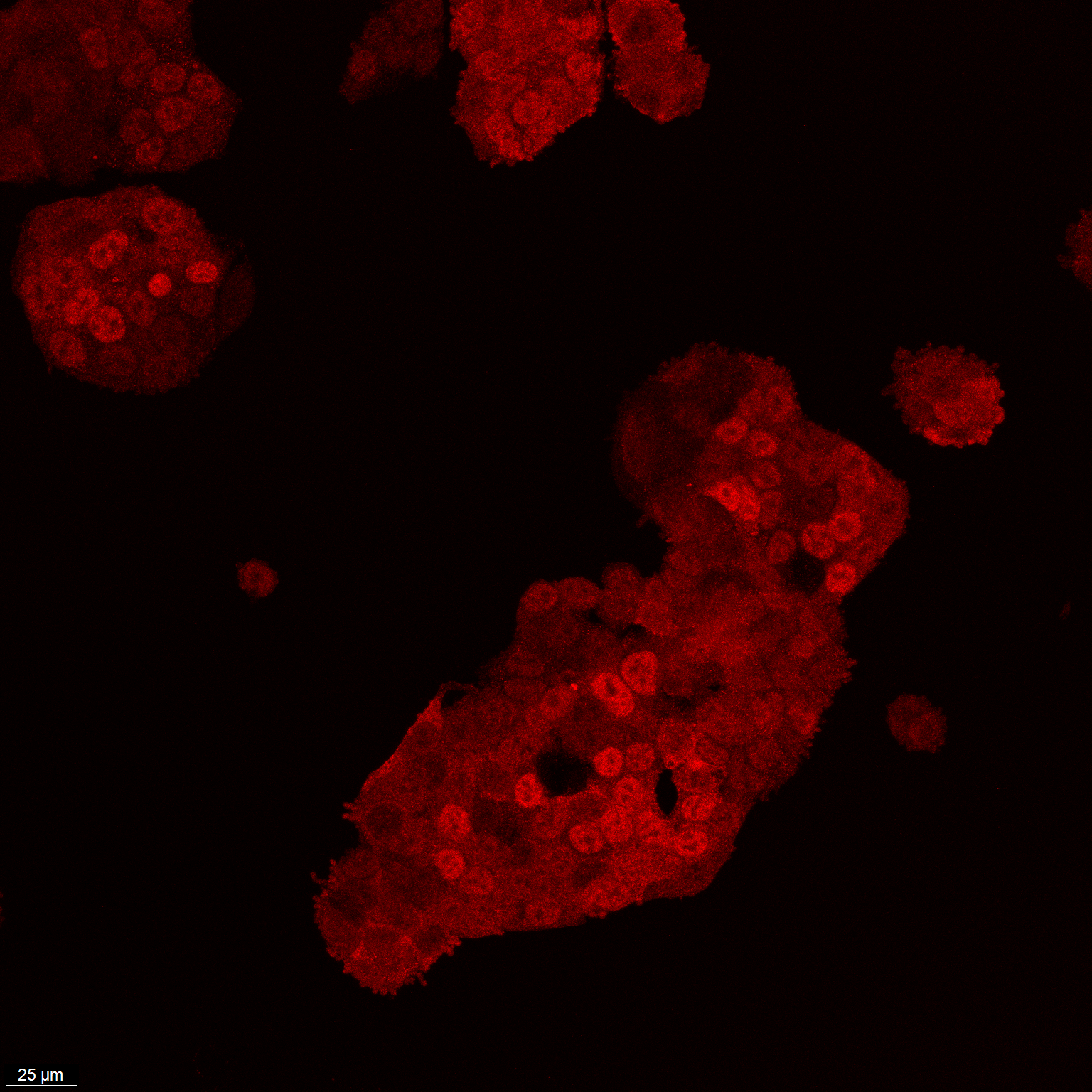

Supplement: Supplementary file 5 — Source data Fig. 3 [file 44319_2025_641_MOESM5_ESM.zip › Fig3/Fig3/3E/3E - FOXO1/G12D.tif]

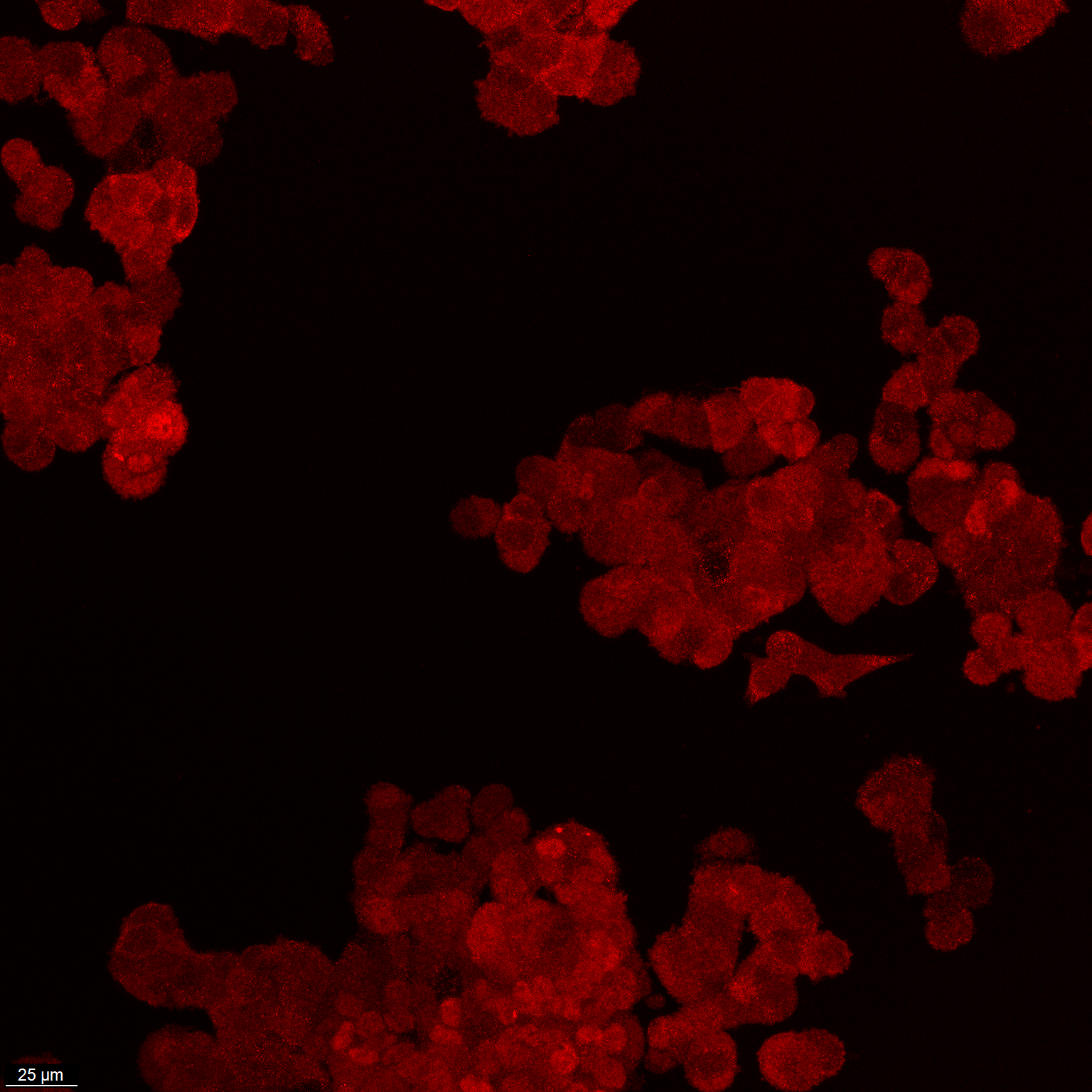

Supplement: Supplementary file 5 — Source data Fig. 3 [file 44319_2025_641_MOESM5_ESM.zip › Fig3/Fig3/3E/3E - FOXO1/G12V.tif]

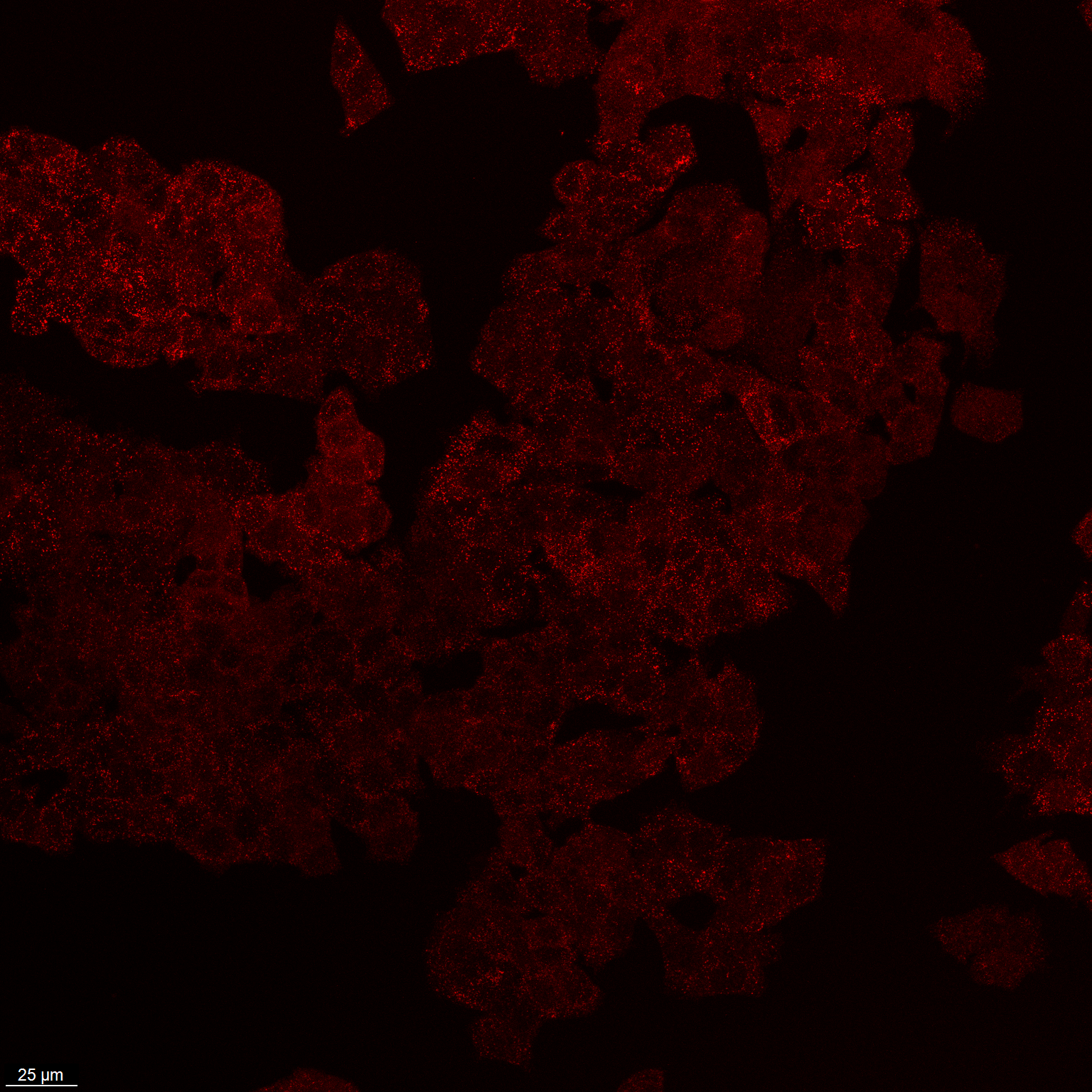

Supplement: Supplementary file 5 — Source data Fig. 3 [file 44319_2025_641_MOESM5_ESM.zip › Fig3/Fig3/3E/3E - FOXO1/WT.tif]

# A.

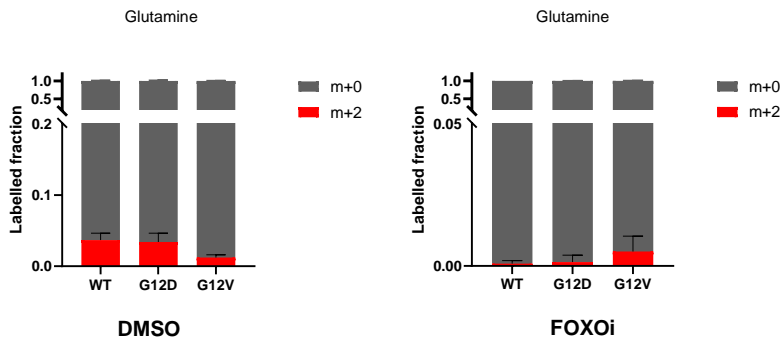

# B.

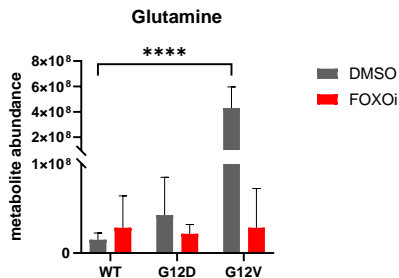

Supplement: Supplementary file 5 — Source data Fig. 3 [file 44319_2025_641_MOESM5_ESM.zip › Fig3/Fig3/Metabolomics_13C_glucose_iFOXO1_ALL/13C labelling_DMSO_FOXOi_Fractions and total.pdf]

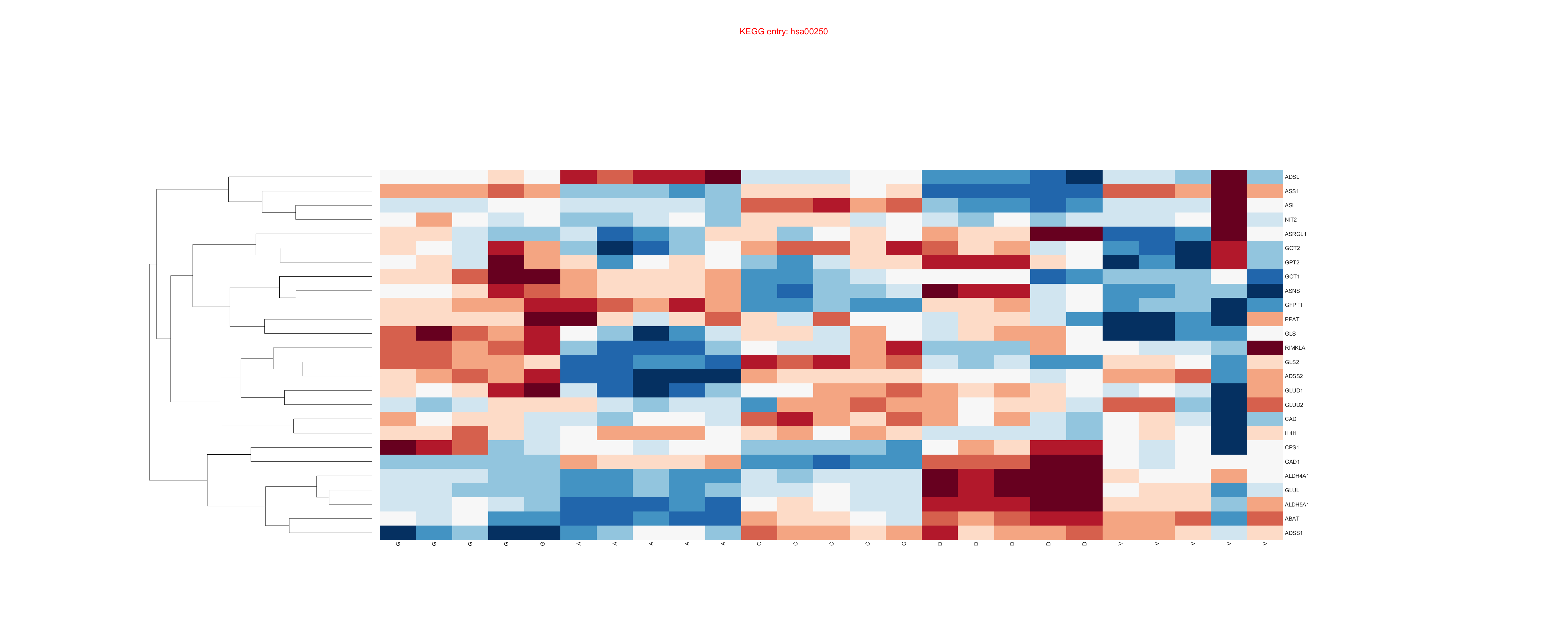

Supplement: Supplementary file 6 — Source data Fig. 4 [file 44319_2025_641_MOESM6_ESM.zip › Fig4/Fig 4C-D/output/hsa002501_cnt.png]

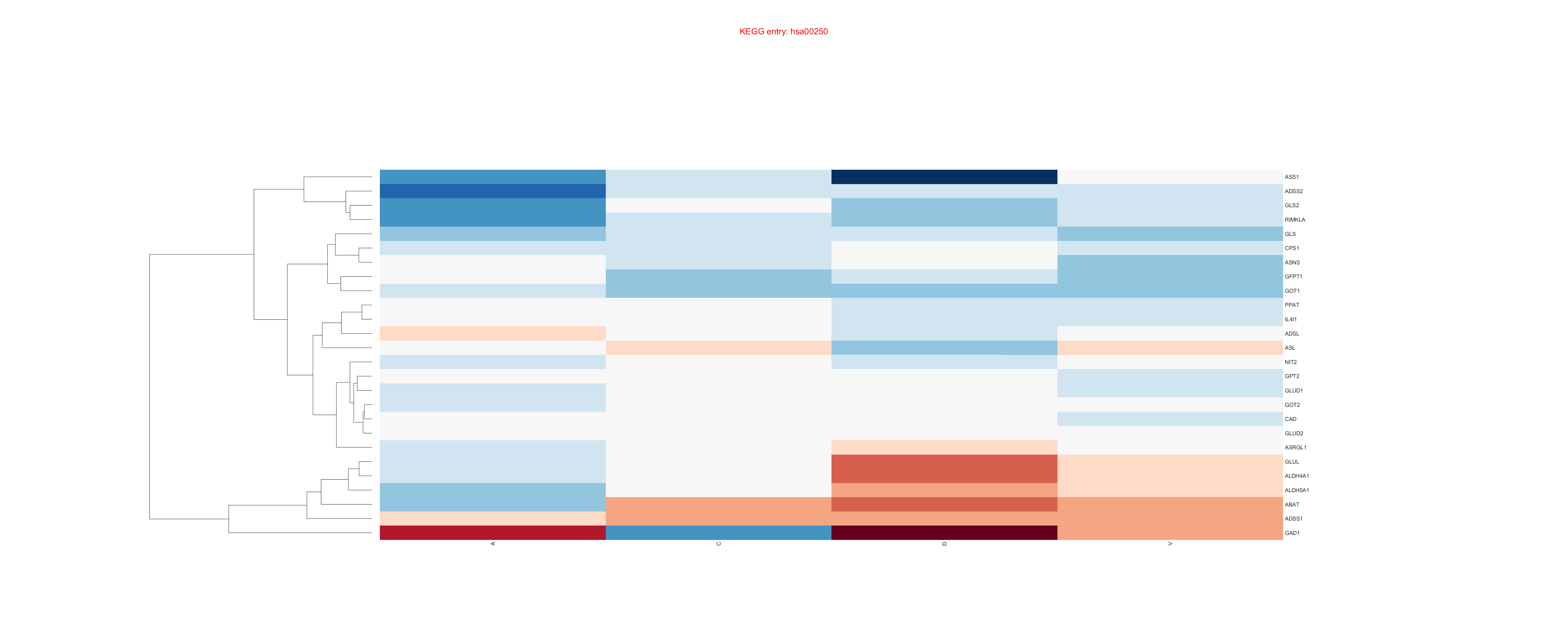

Supplement: Supplementary file 6 — Source data Fig. 4 [file 44319_2025_641_MOESM6_ESM.zip › Fig4/Fig 4C-D/output/hsa002501_fld.png]

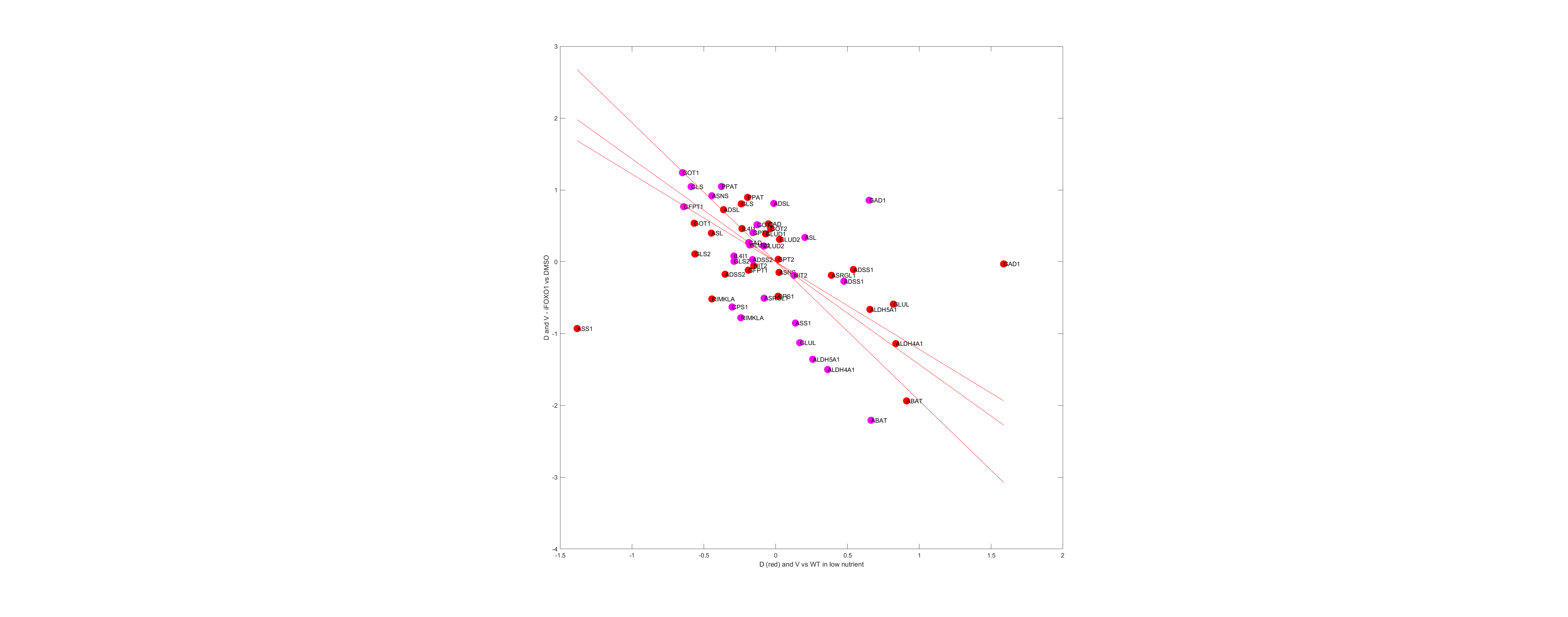

Supplement: Supplementary file 6 — Source data Fig. 4 [file 44319_2025_641_MOESM6_ESM.zip › Fig4/Fig 4C-D/output/hsa00250comparison_fld.png]

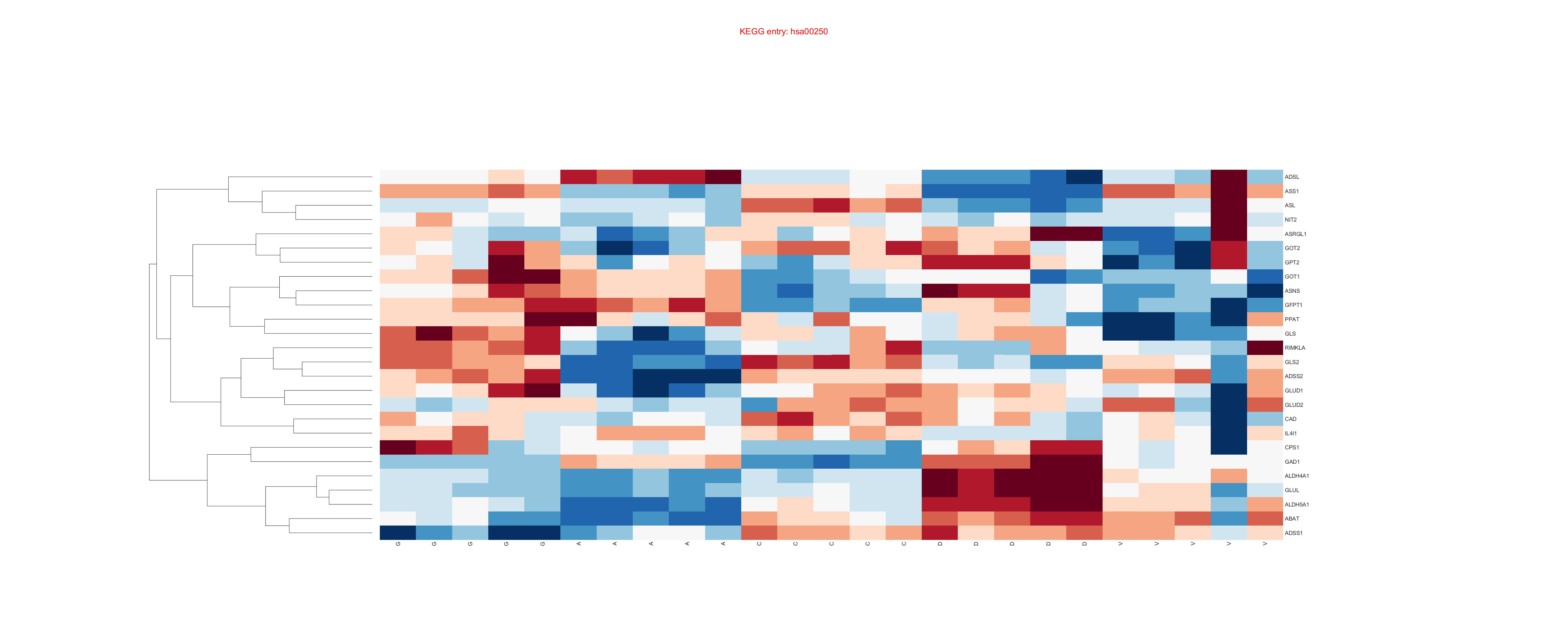

Supplement: Supplementary file 6 — Source data Fig. 4 [file 44319_2025_641_MOESM6_ESM.zip › Fig4/Fig 4C-D/output/hsa00250_cnt.png]

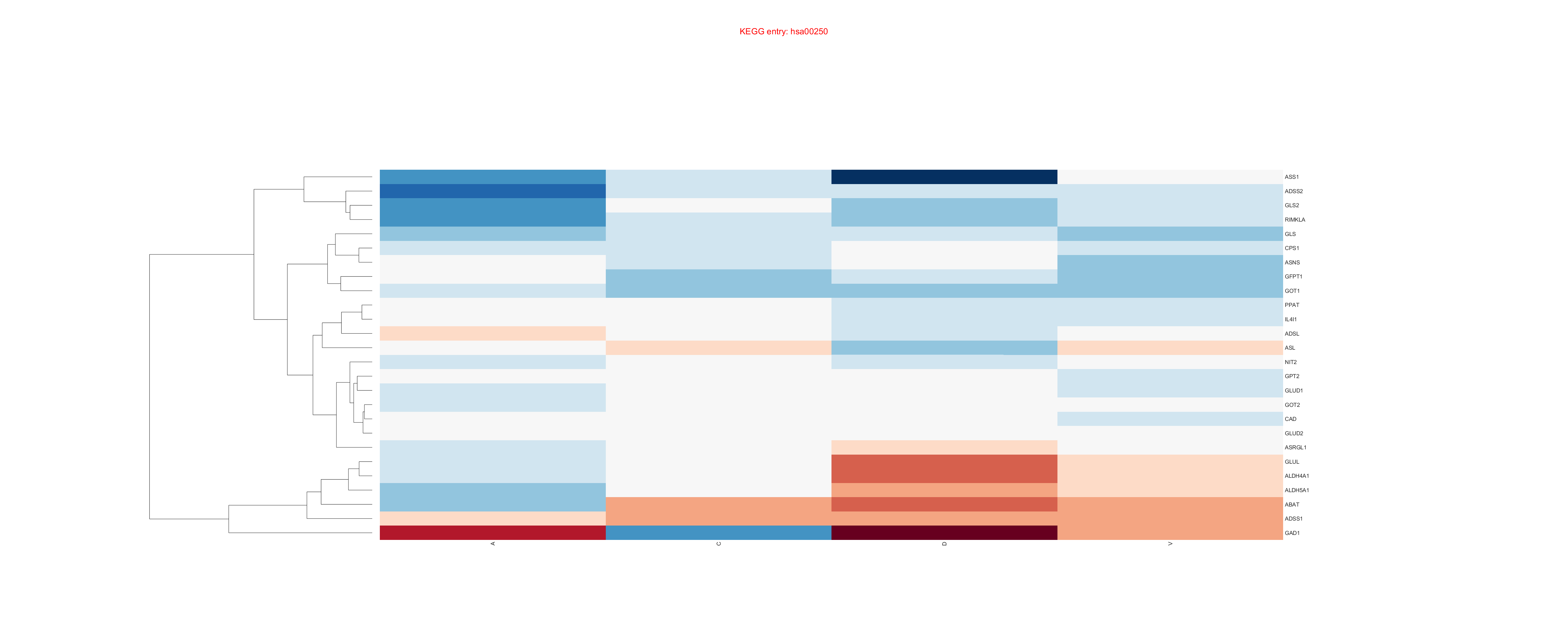

Supplement: Supplementary file 6 — Source data Fig. 4 [file 44319_2025_641_MOESM6_ESM.zip › Fig4/Fig 4C-D/output/hsa00250_fld.png]

# A.

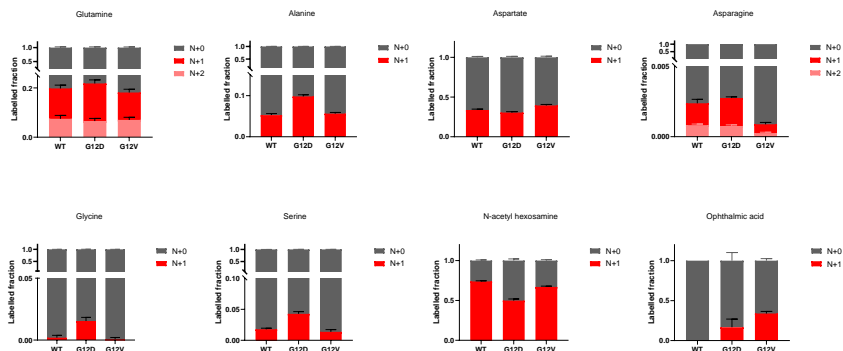

# B.

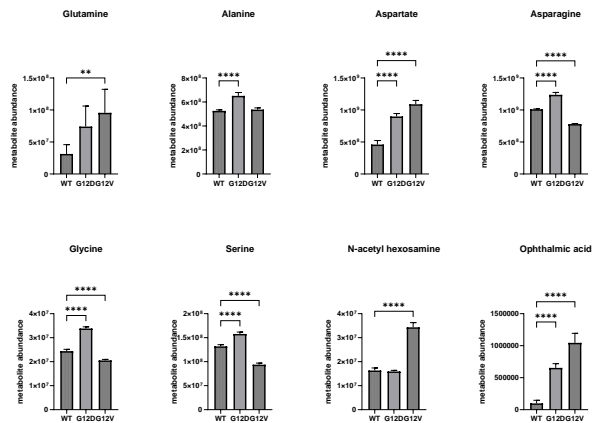

Supplement: Supplementary file 6 — Source data Fig. 4 [file 44319_2025_641_MOESM6_ESM.zip › Fig4/Metabolomics_Ammonia_ALL/Ber_et_al_15N_ammonia_glutamine/15N_Ammonia_Fractions and total.pdf]

**A.**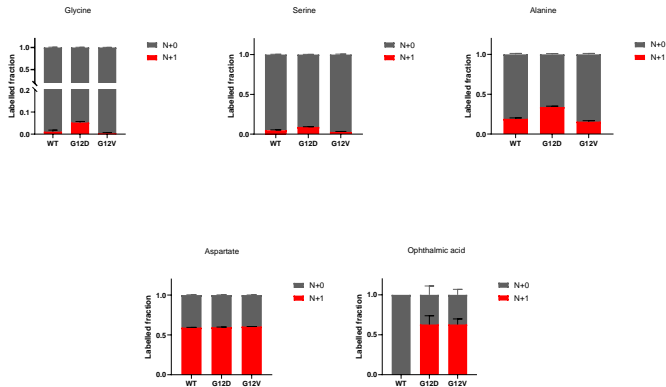**B.**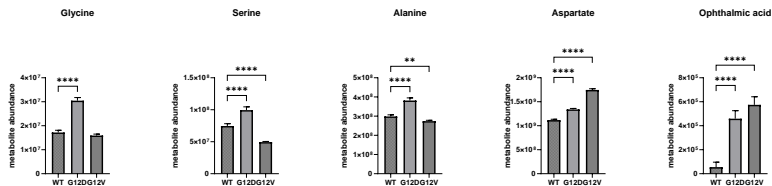

Supplement: Supplementary file 6 — Source data Fig. 4 [file 44319_2025_641_MOESM6_ESM.zip › Fig4/Metabolomics_Ammonia_ALL/Ber_et_al_15N_ammonia_glutamine/15_Alpha_Fractions and total.pdf]

# A.

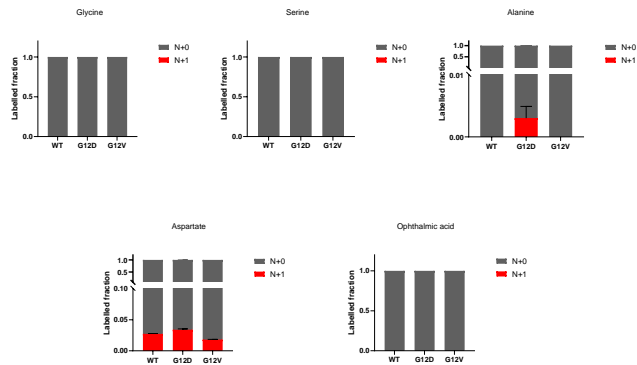

# B.

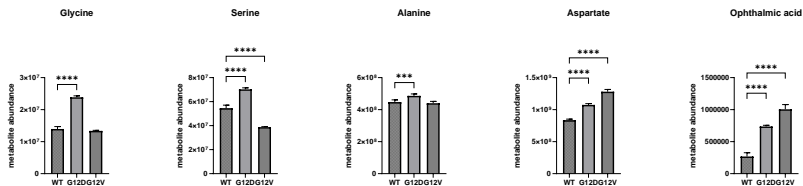

Supplement: Supplementary file 6 — Source data Fig. 4 [file 44319_2025_641_MOESM6_ESM.zip › Fig4/Metabolomics_Ammonia_ALL/Ber_et_al_15N_ammonia_glutamine/15_Amide_Fractions and total.pdf]

# A.

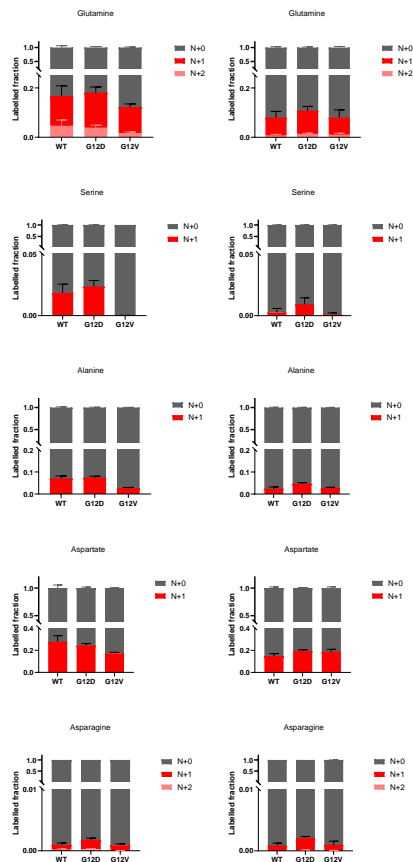

DMSO

FOXOi

# B.

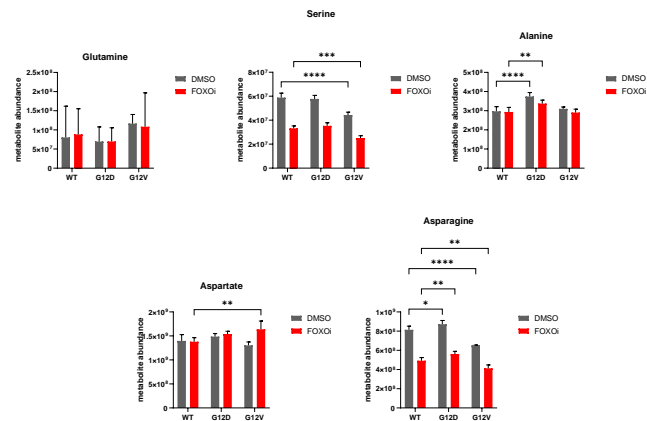

Supplement: Supplementary file 6 — Source data Fig. 4 [file 44319_2025_641_MOESM6_ESM.zip › Fig4/Metabolomics_Ammonia_ALL/Ber_et_al_15N_ammonia_iFOXO1/15N_DMSO_FOXOi_Ammonia_Fractions and total.pdf]
