# Supplementary material for: FOXO1 links KRAS G12D and G12V alleles to glutamine and nitrogen metabolism in colorectal cancer
Source: EMBO Rep. 2025 Nov 20;27(1):142–62. doi: 10.1038/s44319-025-00641-z (PMC12795846; doi:10.1038/s44319-025-00641-z)
Supplement: Supplementary file 8 — Appendix Figures Source Data [file 44319_2025_641_MOESM8_ESM.zip › FigS1.zip!/FigS1/FigS1F-J/MATLAB_KRAS_SW48/2024_fdr005_fc1all_diff_equal_fig1B/pca_biplot.pdf]

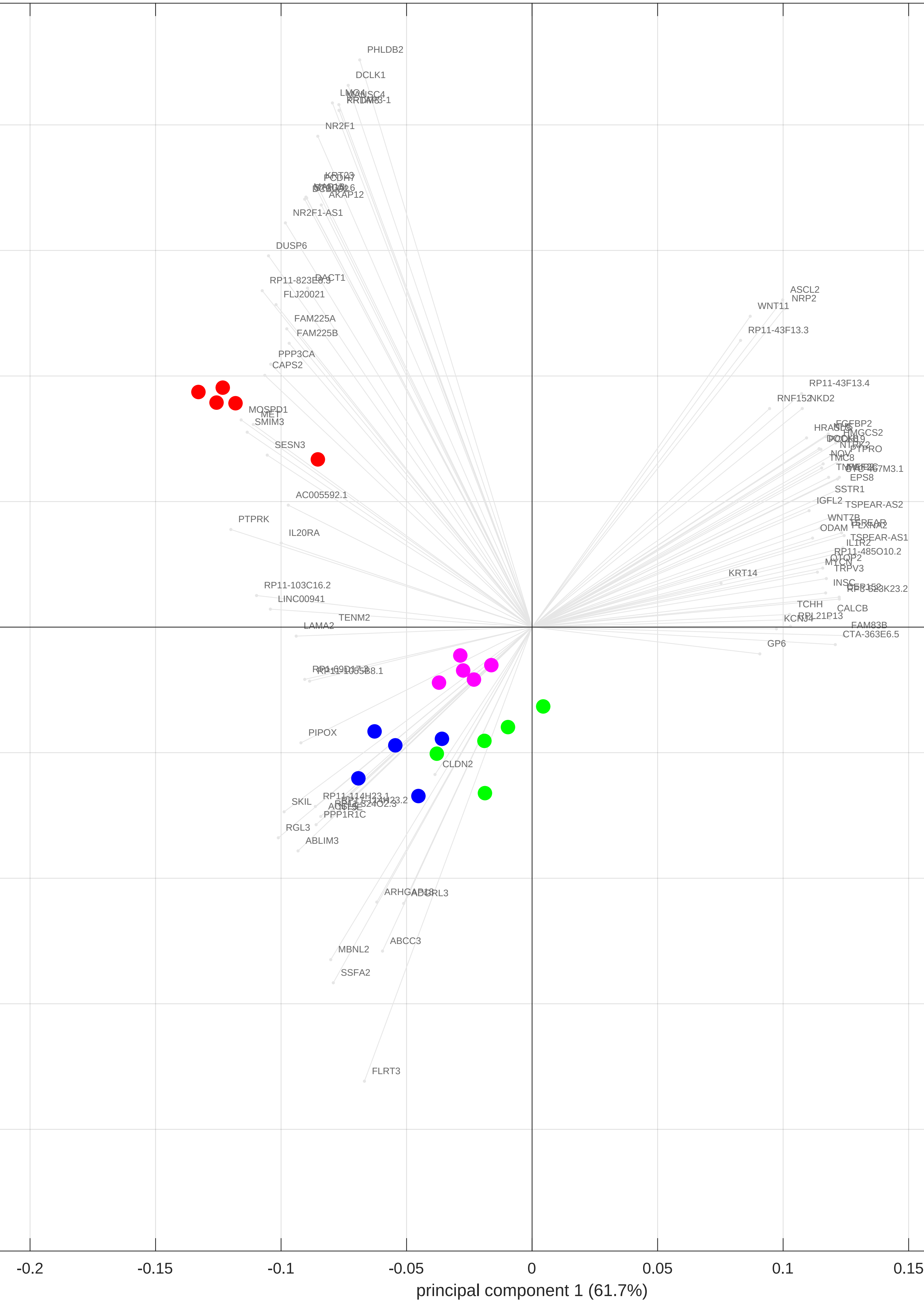
PHLDB2
DCLK1
NR2F1
AKAP12
NR2F1-AS1
DUSP6
FLJ20021
FAM225A
FAM225B
PPP3CA
CAPS2
SMIM3
SESN3
AC005592.1
PTPRK
IL20RA
RP11-103C16.2
LINC00941
TENM2
LAMA2
PIPOX
CLDN2
SKIL
PPP1R1C
RGL3
ABLIM3
ABCC3
MBNL2
SSFA2
FLRT3
ASCL2
NRP2
WNT11
RP11-43F13.3
RP11-43F13.4
EPS8
SSTR1
IGFL2
TSPEAR-AS2
ODAM
TSPEAR-AS1
TRPV3
KRT14
TCHH
CALCB
CTA-363E6.5
GP6
-0.2
-0.15
-0.1
-0.05
0
0.05
0.1
0.15
principal component 1 (61.7%)
