# Supplementary material for: FOXO1 links KRAS G12D and G12V alleles to glutamine and nitrogen metabolism in colorectal cancer
Source: EMBO Rep. 2025 Nov 20;27(1):142–62. doi: 10.1038/s44319-025-00641-z (PMC12795846; doi:10.1038/s44319-025-00641-z)
Supplement: Supplementary file 8 — Appendix Figures Source Data [file 44319_2025_641_MOESM8_ESM.zip › FigS1.zip!/FigS1/FigS1F-J/MATLAB_KRAS_SW48/2024_fdr005_fc1all_diff_equal_fig1B/vulcano.pdf]

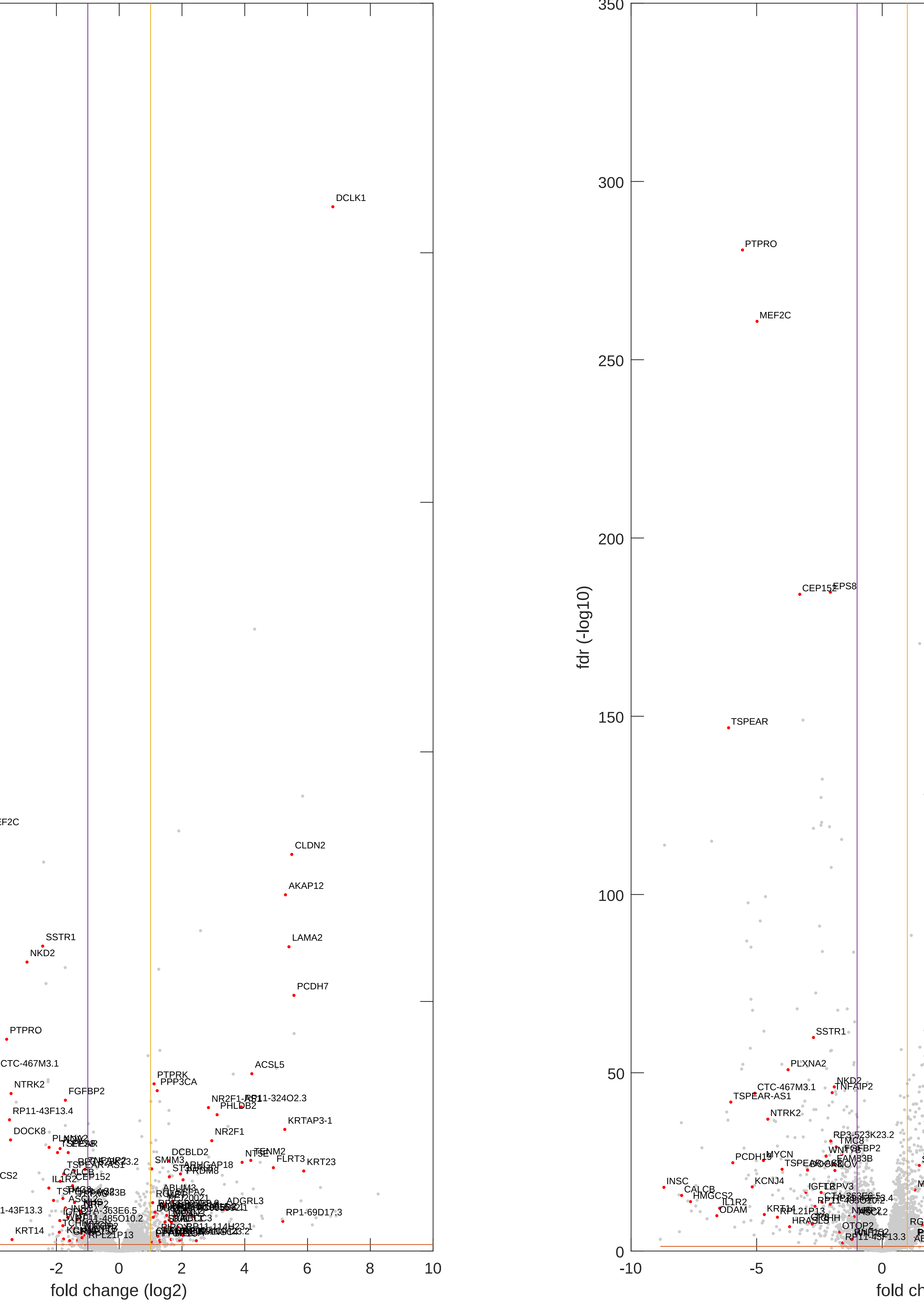

fold change (log2)
fdr (-log10)
-2
0
2
4
6
8
10
-10
-5
0
50
100
150
200
250
300
350
DCLK1
CLDN2
AKAP12
LAMA2
PCDH7
SSTR1
NKD2
PTPRO
CTC-467M3.1
NTRK2
FGFBP2
RP11-43F13.4
DOCK8
ACSL5
PTPRK
PPP3CA
PHLDB2
KRTAP3-1
NR2F1
DCBLD2
SMIM3
ARHGAP18
PRDM8
TENM2
FLRT3
KRT23
ADGRL3
RP1-69D17.3
RP11-114H23.1
KRT14
RPL21P13
IL1R2
CEP152
MEF2C
EPS8
TSPEAR
PLXNA2
TNFAIP2
TSPEAR-AS1
MYCN
PCDH19
KCNJ4
INSC
CALCB
HMGCS2
ODAM
KRT14
HRASLS
OTOP2
TMC8
FAM83B
IGFL2
TRPV3
SSTR1
RP3-523K23.2
RP11-43F13.3
