# Supplementary material for: FOXO1 links KRAS G12D and G12V alleles to glutamine and nitrogen metabolism in colorectal cancer
Source: EMBO Rep. 2025 Nov 20;27(1):142–62. doi: 10.1038/s44319-025-00641-z (PMC12795846; doi:10.1038/s44319-025-00641-z)
Supplement: Supplementary file 8 — Appendix Figures Source Data [file 44319_2025_641_MOESM8_ESM.zip › FigS1.zip!/FigS1/FigS1F-J/MATLAB_KRAS_SW48/2024_fdr005_fc1atleastone_fig1A/clustergram.pdf]

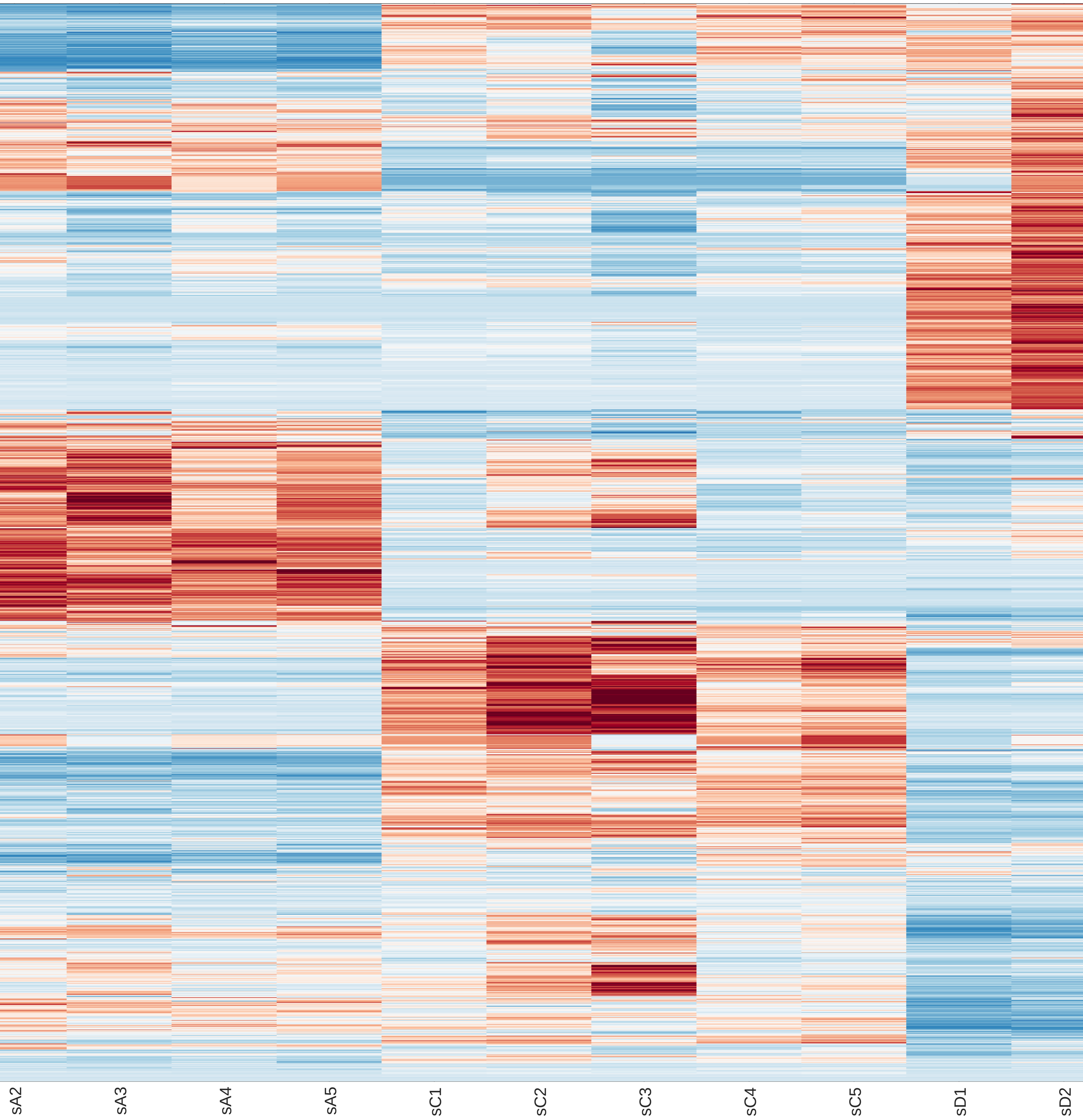
sA2
sA3
sA4
sA5
sC1
sC2
sC3
sC4
sC5
sD1
sD2
