# Supplementary material for: FOXO1 links KRAS G12D and G12V alleles to glutamine and nitrogen metabolism in colorectal cancer
Source: EMBO Rep. 2025 Nov 20;27(1):142–62. doi: 10.1038/s44319-025-00641-z (PMC12795846; doi:10.1038/s44319-025-00641-z)
Supplement: Supplementary file 8 — Appendix Figures Source Data [file 44319_2025_641_MOESM8_ESM.zip › FigS1.zip!/FigS1/FigS1F-J/MATLAB_KRAS_SW48/2024_fdr005_fc1atleastone_fig1A/vulcano.pdf]

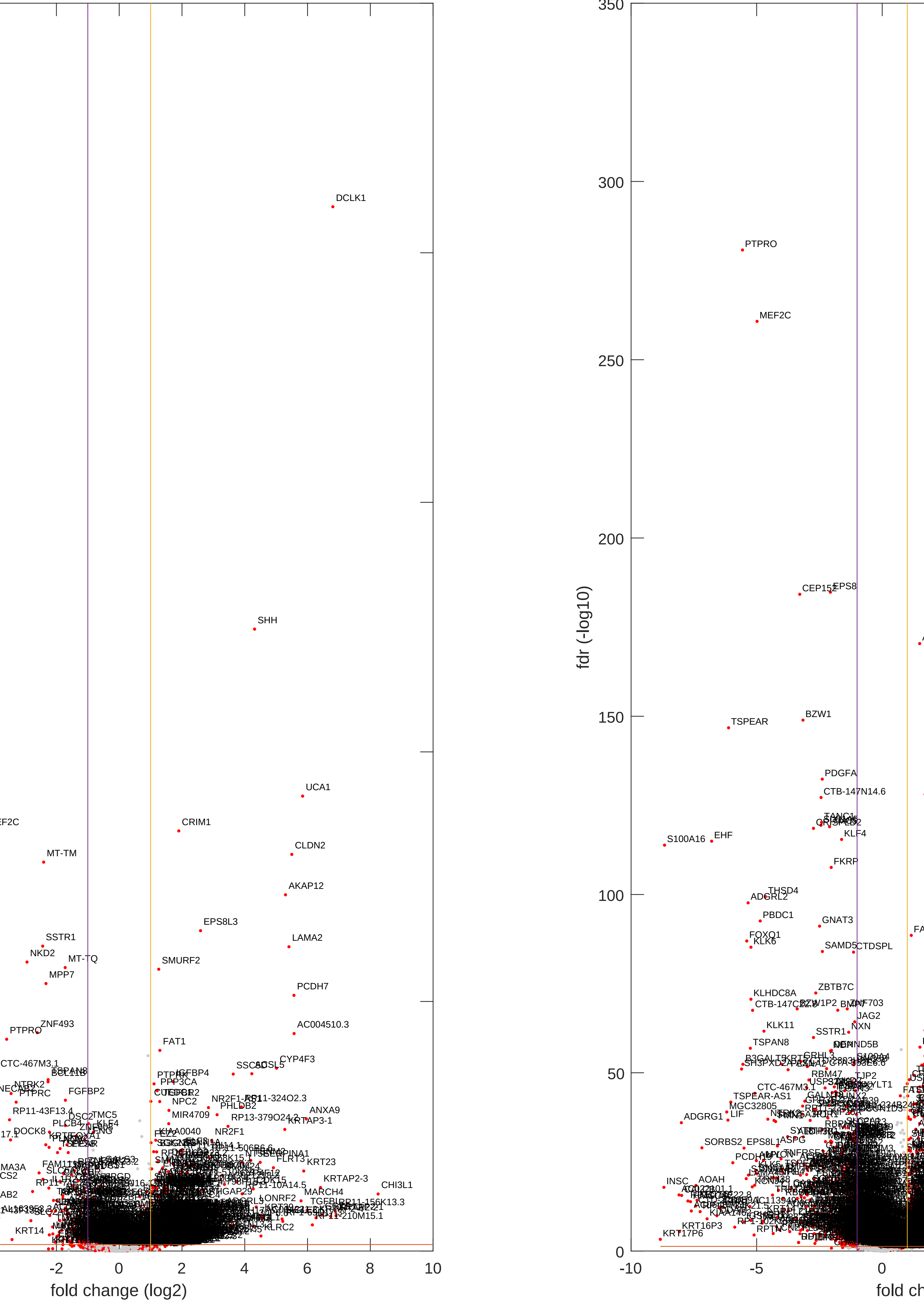

fdr (-log10)
fold change (log2)
fold ch
-2
0
2
4
6
8
10
-10
-5
0
50
100
150
200
250
300
350
DCLK1
SHH
UCA1
CRIM1
CLDN2
MT-TM
AKAP12
EPS8L3
LAMA2
SSTR1
NKD2
MT-TQ
SMURF2
MPP7
PCDH7
ZNF493
PTPRO
AC004510.3
FAT1
CTC-467M3.1
CYP4F3
SSC5D
IGFBP4
PPP3CA
FGFBP2
NPC2
MIR4709
PHLDB2
ANXA9
KRTAP3-1
NR2F1
KIAA0040
FLRT3
KRT23
KRTAP2-3
CHI3L1
MARCH4
RP11-10A14.5
KRTAP2-3
RP11-156K13.3
RP11-210M15.1
KLRC2
KRT14
PTPRO
MEF2C
CEP152
EPS8
TSPEAR
BZW1
PDGFA
CTB-147N14.6
S100A16
EHF
KLF4
FKRP
THSD4
ADGRL2
PBDC1
GNAT3
FOXQ1
KLK6
SAMD5
CTDSPL
KLHDC8A
ZBTB7C
BMP7
ZNF703
JAG2
KLK11
NXN
SSTR1
TSPAN8
DENND5B
RBM47
CTC-467M3.1
TSPEAR-AS1
MGC32805
LIF
ADGRG1
SORBS2
EPS8L1
INSC
AOAH
KRT16P3
KRT17P6
