# Supplementary material for: FOXO1 links KRAS G12D and G12V alleles to glutamine and nitrogen metabolism in colorectal cancer
Source: EMBO Rep. 2025 Nov 20;27(1):142–62. doi: 10.1038/s44319-025-00641-z (PMC12795846; doi:10.1038/s44319-025-00641-z)
Supplement: Supplementary file 8 — Appendix Figures Source Data [file 44319_2025_641_MOESM8_ESM.zip › FigS1.zip!/FigS1/FigS1F-J/MATLAB_KRAS_SW48/2024_fdr005_fc1topgenes_A/vulcano.pdf]

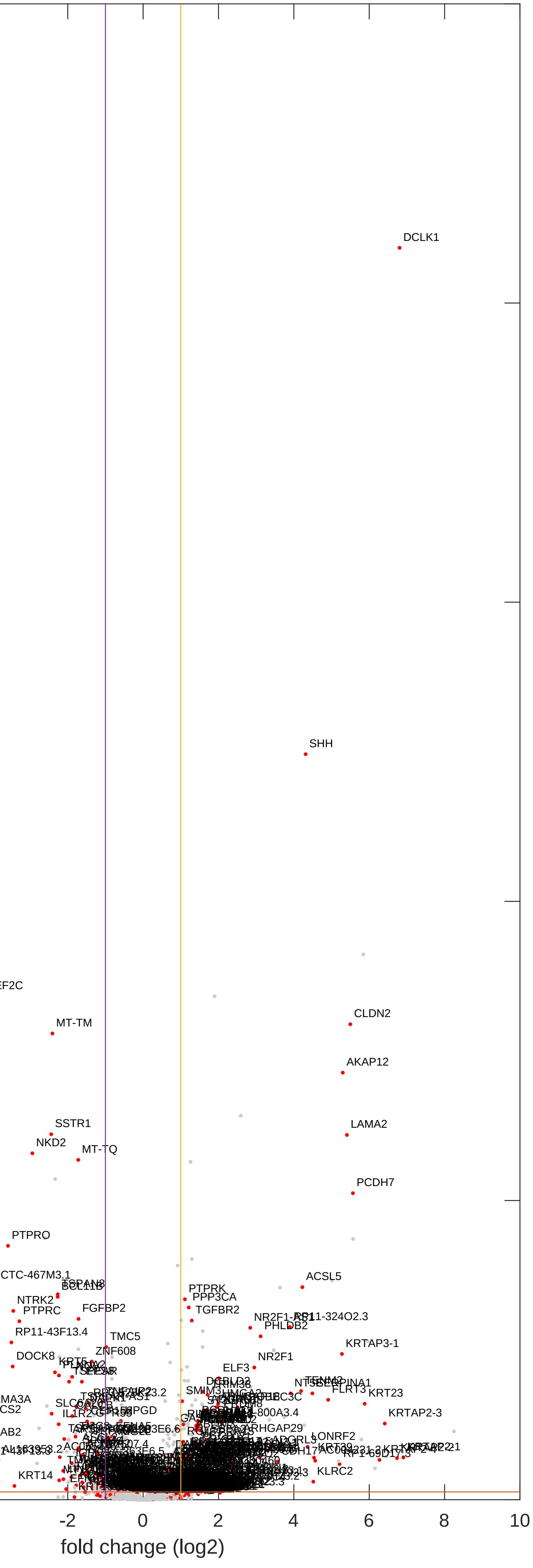

DCLK1
SHH
CLDN2
AKAP12
LAMA2
PCDH7
MT-TM
SSTR1
NKD2
MT-TQ
PTPRO
CTC-467M3.1
NTRK2
PTPRC
FGFBP2
RP11-43F13.4
TMC5
ZNF608
DOCK8
PTPRK
PPP3CA
TGFBR2
ACSL5
PHLDB2
NR2F1
ELF3
KRTAP3-1
KRT23
KRTAP2-3
FLRT3
LONRF2
KLRC2
KRT14
-2
0
2
4
6
8
10
fold change (log2)

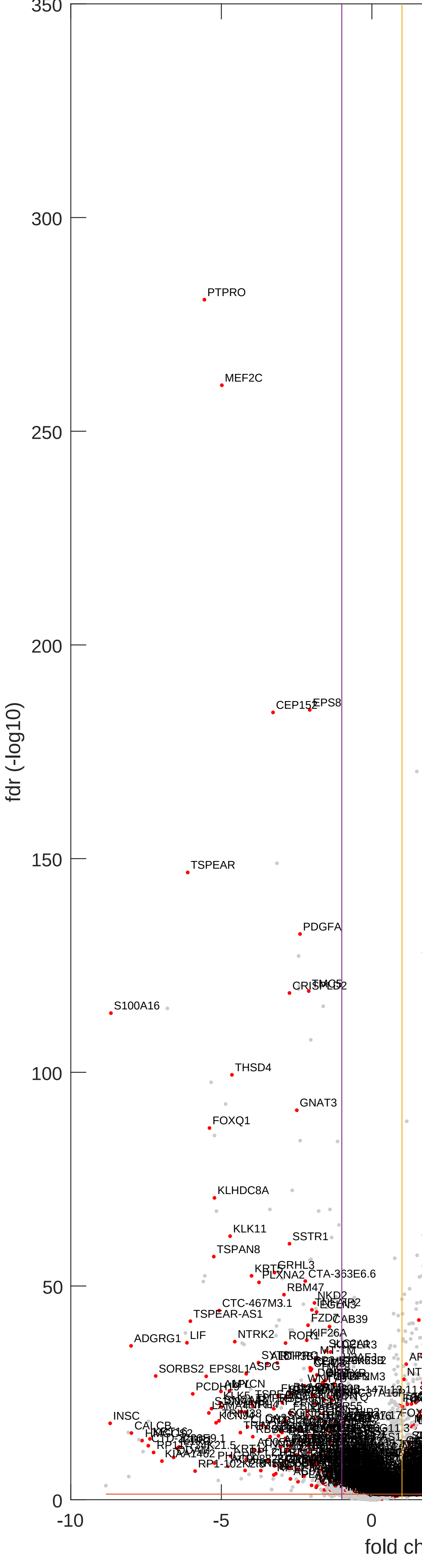

350
300
250
200
150
100
50
0
fdr (-log10)
PTPRO
MEF2C
CEP152
EPS8
TSPEAR
PDGFA
S100A16
THSD4
GNAT3
FOXQ1
KLHDC8A
KLK11
SSTR1
TSPAN8
CTA-363E6.6
RBM47
CTC-467M3.1
TSPEAR-AS1
ADGRG1
LIF
NTRK2
KIF26A
SORBS2
EPS8L1
ASPG
INSC
-10
-5
0
