# Supplementary material for: FOXO1 links KRAS G12D and G12V alleles to glutamine and nitrogen metabolism in colorectal cancer
Source: EMBO Rep. 2025 Nov 20;27(1):142–62. doi: 10.1038/s44319-025-00641-z (PMC12795846; doi:10.1038/s44319-025-00641-z)
Supplement: Supplementary file 8 — Appendix Figures Source Data [file 44319_2025_641_MOESM8_ESM.zip › FigS1.zip!/FigS1/FigS1F-J/MATLAB_KRAS_SW48/2024_fdr005_fc1topgenes_C/pca_biplot.pdf]

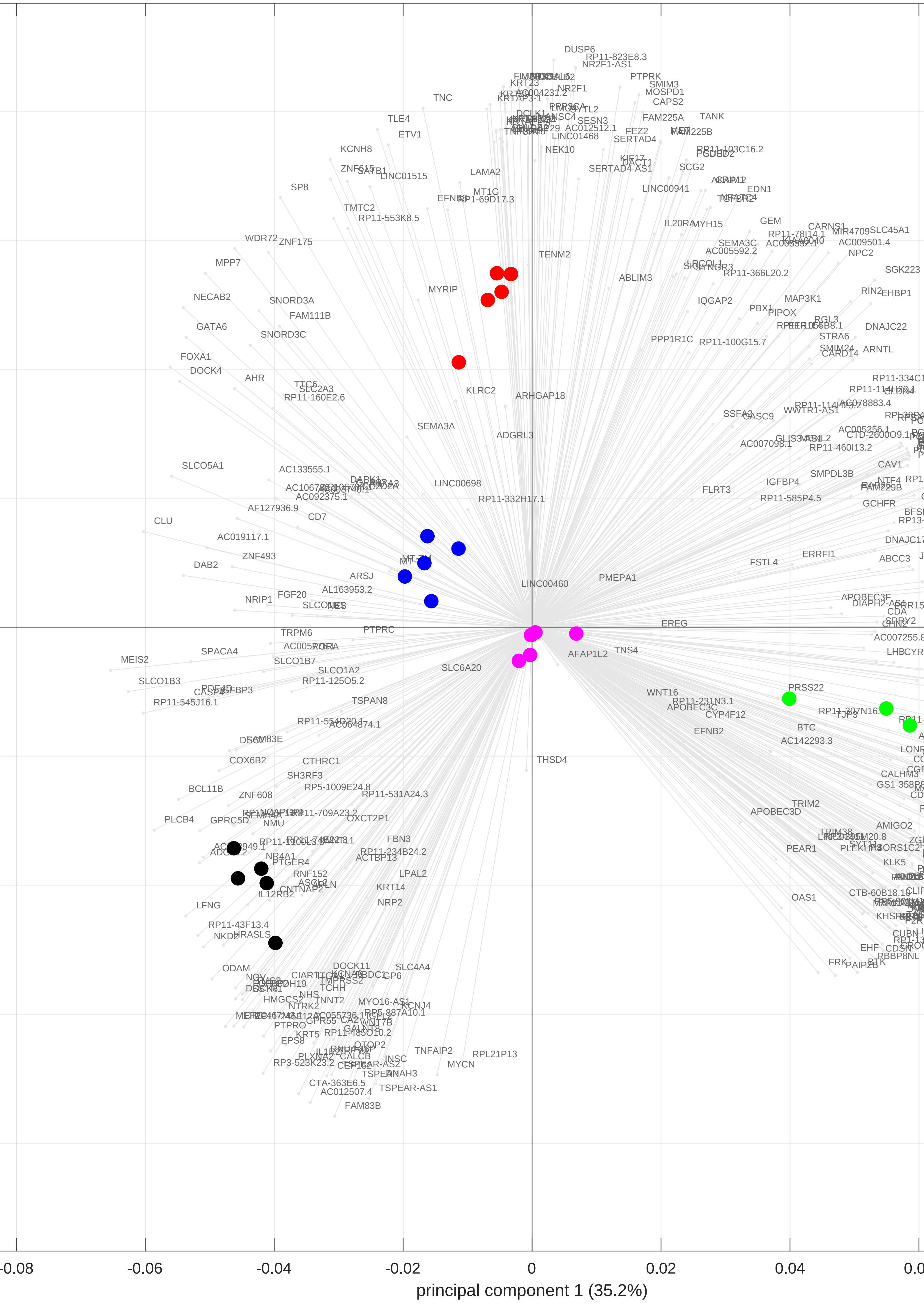

-0.08
-0.06
-0.04
-0.02
0
0.02
0.04
principal component 1 (35.2%)
DUSP6
RP11-823E8.3
NR2F1-AS1
PTPRK
SMIM3
MOSPD1
CAPS2
NR2F1
TNC
TLE4
ETV1
KCNH8
SESN3
FAM225A
TANK
FAM225B
FEZ2
SERTAD4
LINC01468
NEK10
RP11-103C16.2
SCG2
SERTAD4-AS1
LAMA2
LINC01515
SP8
TMTC2
RP11-553K8.5
MT1G
RP1-69D17.3
LINC00941
EDN1
IL20RA
MYH15
GEM
CARNS1
SLC45A1
WDR72
ZNF175
TENM2
SEMA3C
AC005592.2
AC009501.4
NPC2
MPP7
ABLIM3
RP11-366L20.2
SGK223
NECAB2
SNORD3A
MYRIP
IQGAP2
MAP3K1
EHBP1
FAM111B
PIPOX
GATA6
SNORD3C
PPP1R1C
RP11-100G15.7
STRA6
DNAJC22
FOXA1
DOCK4
ARNTL
AHR
SLC2A3
RP11-160E2.6
KLRC2
ARHGAP18
SEMA3A
ADGRL3
SSFA2
CASC9
AC007098.1
RP11-460I13.2
SLCO5A1
AC133555.1
LINC00698
CAV1
SMPDL3B
IGFBP4
FLRT3
RP11-585P4.5
FAM229B
GCHFR
RP11-332H17.1
AC092375.1
AF127936.9
CD7
CLU
AC019117.1
ZNF493
DAB2
ARSJ
AL163953.2
FSTL4
ERRFI1
ABCC3
LINC00460
PMEPA1
NRIP1
FGF20
APOBEC3F
CDA
EREG
TRPM6
PTPRC
MEIS2
SPACA4
SLCO1B7
SLCO1A2
RP11-125O5.2
AFAP1L2
TNS4
SLC6A20
SLCO1B3
RP11-545J16.1
TSPAN8
PRSS22
WNT16
RP11-231N3.1
APOBEC3C
CYP4F12
EFNB2
BTC
AC142293.3
RP11-554D20.1
AC064874.1
COX6B2
CTHRC1
SH3RF3
THSD4
BCL11B
ZNF608
RP5-1009E24.8
RP11-531A24.3
CALHM3
PLCB4
GPRC5D
NMU
OXCT2P1
TRIM2
APOBEC3D
AMIGO2
FBN3
RP11-234B24.2
ACTBP13
PEAR1
NR4A1
PTGER4
KLK5
RNF152
LPAL2
KRT14
IL12RB2
NRP2
OAS1
LFNG
RP11-43F13.4
ODAM
EHF
FRK
DOCK11
SLC4A4
CIART
TMPRSS2
TCHH
HMGCS2
NTRK2
TNNT2
MYO16-AS1
KCNJ4
PTPRO
EPS8
KRT5
RP11-485O10.2
OTOP2
CALCB
TNFAIP2
RPL21P13
MYCN
RP3-523K23.2
TSPEAR-AS2
DNAH3
CTA-363E6.5
AC012507.4
TSPEAR-AS1
FAM83B
