# Supplementary material for: FOXO1 links KRAS G12D and G12V alleles to glutamine and nitrogen metabolism in colorectal cancer
Source: EMBO Rep. 2025 Nov 20;27(1):142–62. doi: 10.1038/s44319-025-00641-z (PMC12795846; doi:10.1038/s44319-025-00641-z)
Supplement: Supplementary file 8 — Appendix Figures Source Data [file 44319_2025_641_MOESM8_ESM.zip › FigS1.zip!/FigS1/FigS1F-J/MATLAB_KRAS_SW48/2024_fdr005_fc1topgenes_C/vulcano.pdf]

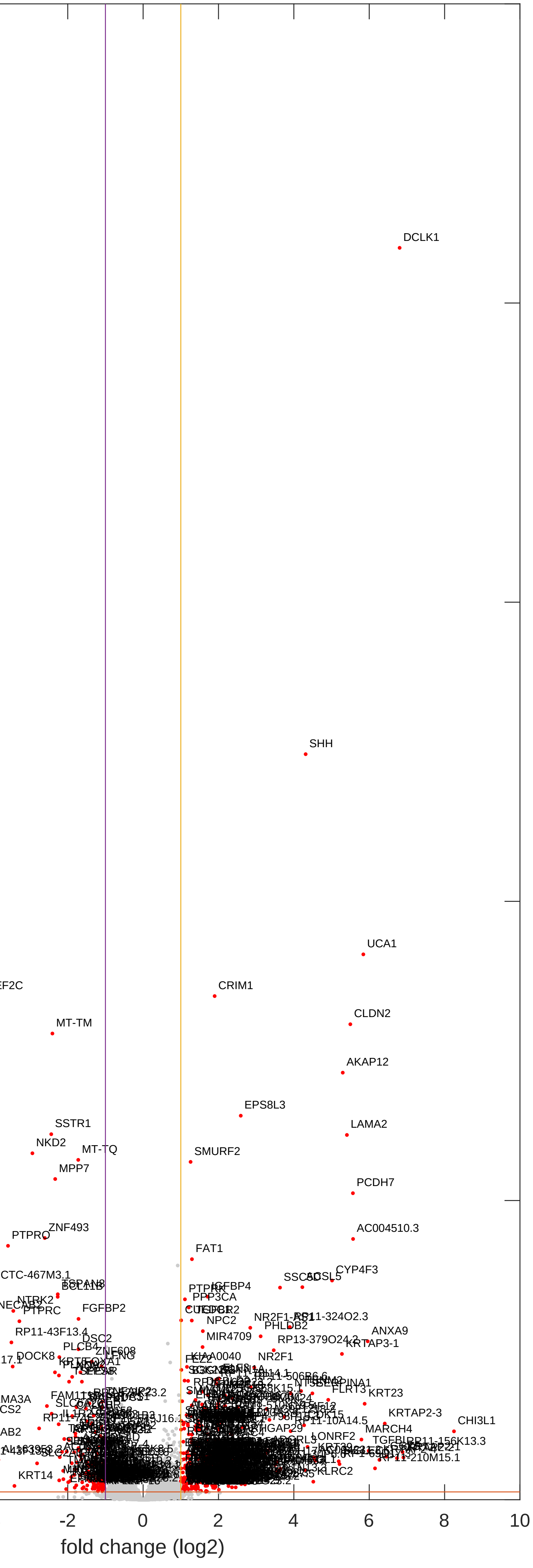
DCLK1
SHH
UCA1
CRIM1
CLDN2
MT-TM
AKAP12
EPS8L3
SSTR1
LAMA2
NKD2
MT-TQ
SMURF2
MPP7
PCDH7
ZNF493
PTPRO
AC004510.3
FAT1
CYP4F3
IGFBP4
PPP3CA
FGFBP2
NPC2
MIR4709
PHLDB2
ANXA9
KRTAP3-1
NR2F1
KRT23
KRTAP2-3
CHI3L1
MARCH4
KRT14
RP11-210M15.1
KLRC2
-2
0
2
4
6
8
10
fold change (log2)

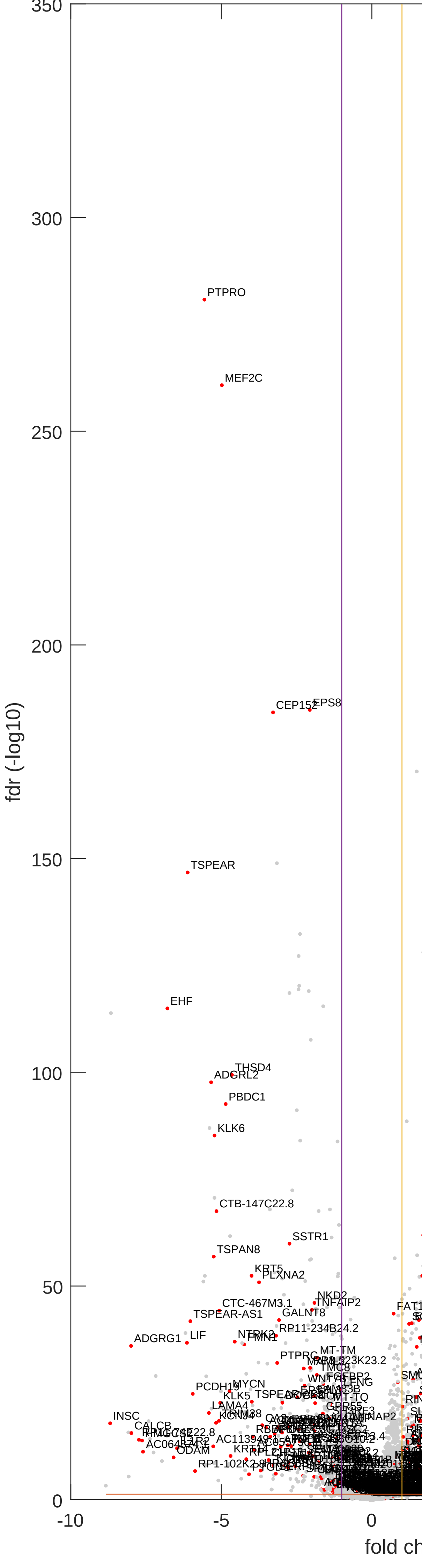
350
300
250
200
150
100
50
0
fdr (-log10)
PTPRO
MEF2C
CEP152
EPS8
TSPEAR
EHF
THSD4
ADGRL2
PBDC1
KLK6
CTB-147C22.8
SSTR1
TSPAN8
KRT5
PLXNA2
NKD2
TNFAIP2
CTC-467M3.1
GALNT8
TSPEAR-AS1
RP11-234B24.2
ADGRG1
LIF
MT-TM
INSC
-10
-5
0
