# Supplementary material for: FOXO1 links KRAS G12D and G12V alleles to glutamine and nitrogen metabolism in colorectal cancer
Source: EMBO Rep. 2025 Nov 20;27(1):142–62. doi: 10.1038/s44319-025-00641-z (PMC12795846; doi:10.1038/s44319-025-00641-z)
Supplement: Supplementary file 8 — Appendix Figures Source Data [file 44319_2025_641_MOESM8_ESM.zip › FigS1.zip!/FigS1/FigS1F-J/MATLAB_KRAS_SW48/2024_fdr005_fc1topgenes_D/vulcano.pdf]

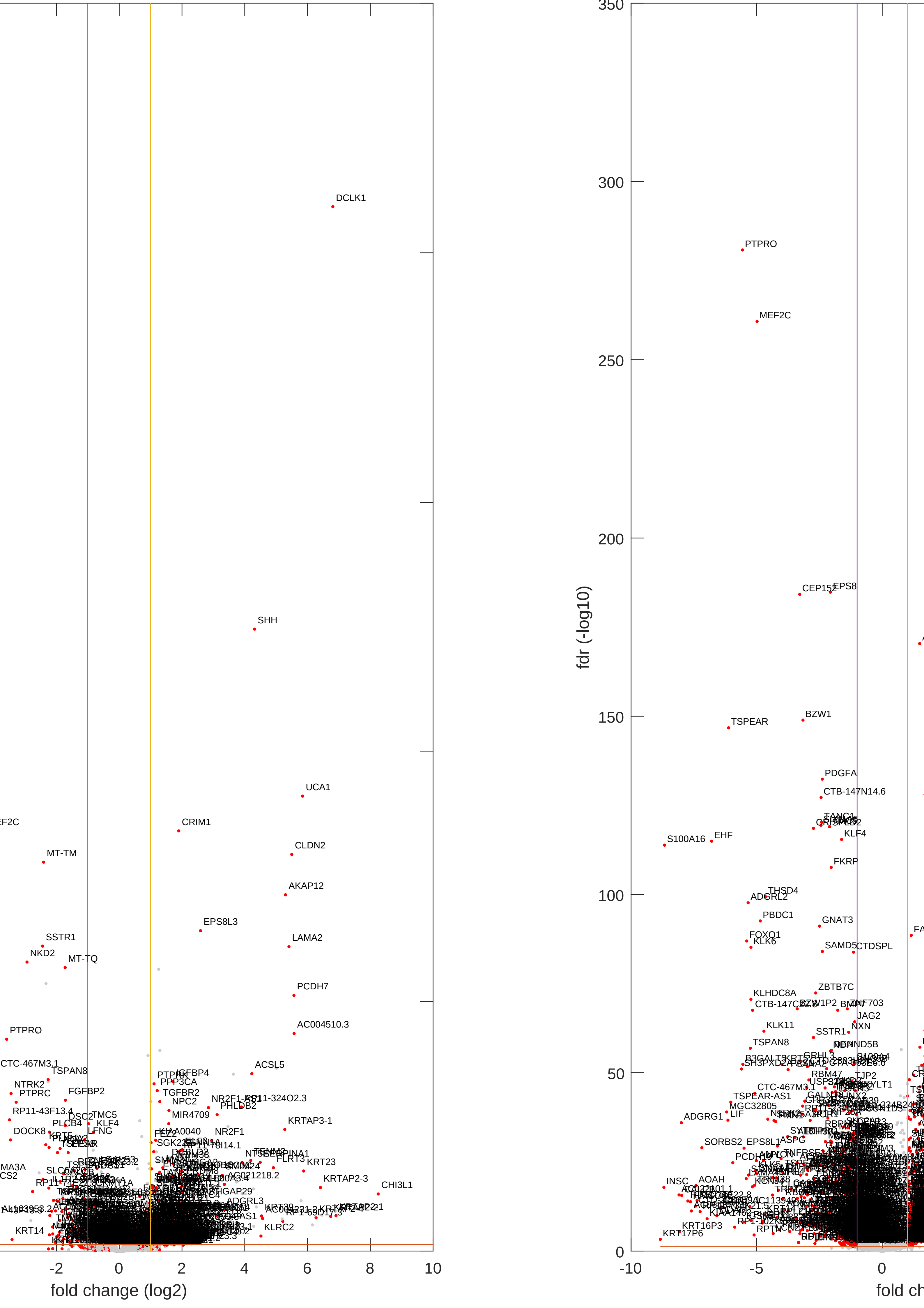

fold change (log2)
-2
0
2
4
6
8
10
DCLK1
SHH
UCA1
CRIM1
CLDN2
MT-TM
AKAP12
EPS8L3
SSTR1
LAMA2
NKD2
MT-TQ
PCDH7
PTPRO
AC004510.3
CTC-467M3.1
TSPAN8
ACSL5
NTRK2
PTPRC
FGFBP2
IGFBP4
PPP3CA
TGFBR2
NPC2
MIR4709
NR2F1
PHLDB2
RP11-43F13.4
DSC2
TMC5
PLCB4
KLF4
LFNG
DOCK8
KRTAP3-1
KIAA0040
KRT23
KRTAP2-3
CHI3L1
ADGRL3
KRT14
KLRC2
fdr (-log10)
350
300
250
200
150
100
50
0
-10
-5
0
PTPRO
MEF2C
CEP152
EPS8
TSPEAR
BZW1
PDGFA
CTB-147N14.6
S100A16
EHF
KLF4
FKRP
THSD4
ADGRL2
PBDC1
GNAT3
FOXQ1
KLK6
SAMD5
CTDSPL
KLHDC8A
ZBTB7C
CTB-147C22.8
BZW1P2
BMP7
ZNF703
JAG2
KLK11
NXN
SSTR1
TSPAN8
DENND5B
RBM47
CTC-467M3.1
TJP2
TSPEAR-AS1
MGC32805
ADGRG1
LIF
SORBS2
EPS8L1
ASPG
INSC
AOAH
KRT16P3
KRT17P6
