# Supplementary material for: FOXO1 links KRAS G12D and G12V alleles to glutamine and nitrogen metabolism in colorectal cancer
Source: EMBO Rep. 2025 Nov 20;27(1):142–62. doi: 10.1038/s44319-025-00641-z (PMC12795846; doi:10.1038/s44319-025-00641-z)
Supplement: Supplementary file 8 — Appendix Figures Source Data [file 44319_2025_641_MOESM8_ESM.zip › FigS1.zip!/FigS1/FigS1F-J/MATLAB_KRAS_SW48/2024_fdr005_fc1topgenes_V/pca_biplot.pdf]

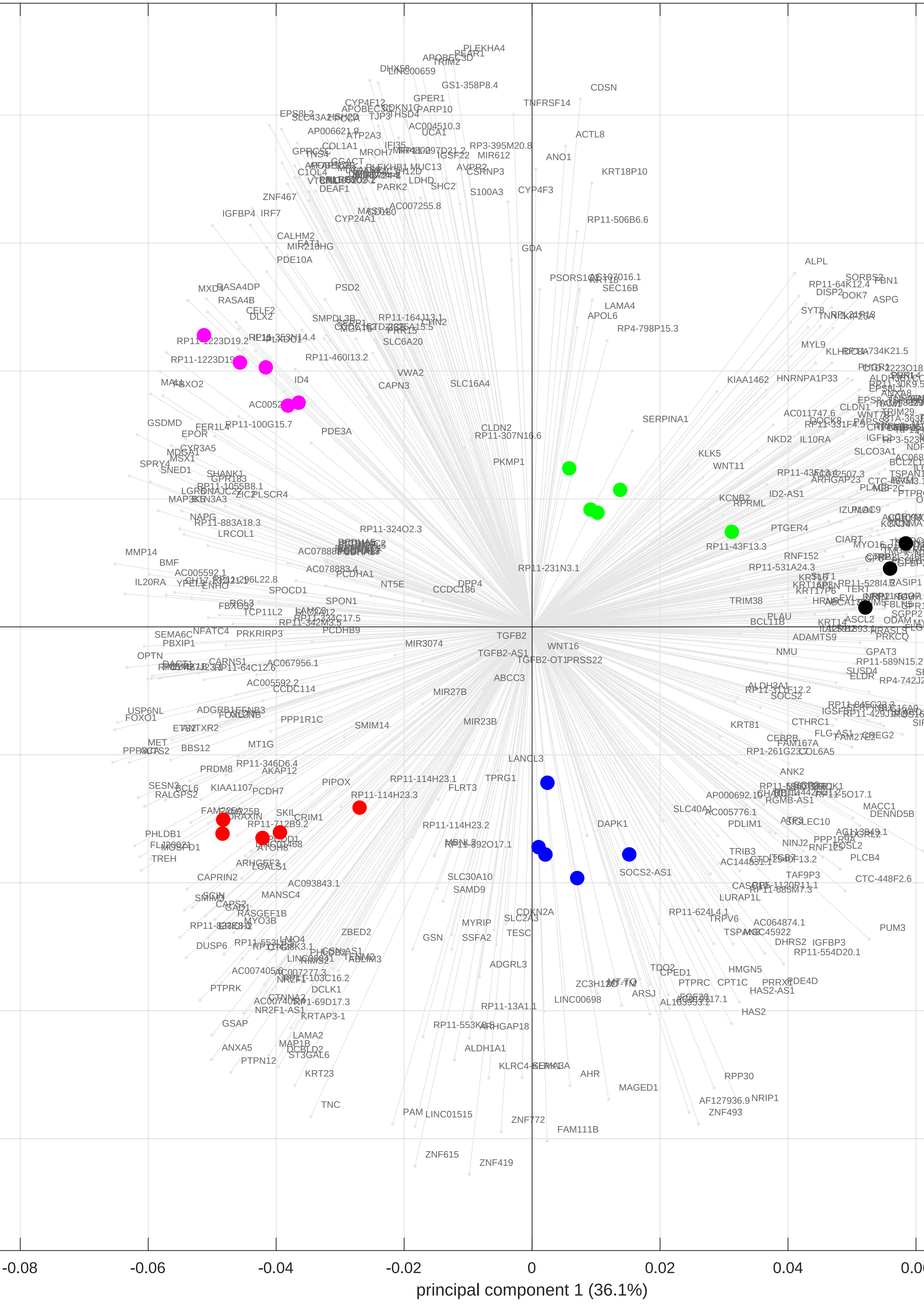

-0.08
-0.06
-0.04
-0.02
0
0.02
0.04
principal component 1 (36.1%)
PLEKHA4
CDSN
TNFRSF14
ACTL8
KRT18P10
ANO1
CYP4F3
RP11-506B6.6
GDA
S100A3
ZNF467
IGFBP4
IRF7
PDE10A
PSD2
RASA4B
SLC6A20
RP11-460I13.2
VWA2
CAPN3
SLC16A4
ID4
GSDMD
EPOR
PDE3A
CLDN2
RP11-307N16.6
PKMP1
SERPINA1
KLK5
WNT11
ALPL
SORBS2
ASPG
MYL9
KIAA1462
HNRNPA1P33
NKD2
IL10RA
ID2-AS1
PTGER4
RP11-43F13.3
RNF152
RP11-531A24.3
TRIM38
MMP14
BMF
IL20RA
SPOCD1
SPON1
NT5E
CCDC186
RP11-231N3.1
RP11-324O2.3
MIR3074
TGFB2
TGFB2-AS1
WNT16
ABCC3
MIR27B
MIR23B
SMIM14
PPP1R1C
OPTN
PBXIP1
SEMA6C
NFATC4
PRKRIPP3
PCDHB9
USP6NL
FOXO1
MET
BBS12
MT1G
PRDM8
AKAP12
PIPOX
LANCL3
TPRG1
FLRT3
RP11-114H23.1
RP11-114H23.3
RP11-114H23.2
DAPK1
SLC40A1
PDLIM1
ANK2
MACC1
DENND5B
PLCB4
TAF9P3
CTC-448F2.6
PUM3
TREH
PHLDB1
CAPRIN2
MANSC4
AC093843.1
SLC30A10
SAMD9
SOCS2-AS1
LURAP1L
RP11-624L4.1
TRPV6
AC064874.1
DHRS2
IGFBP3
RP11-554D20.1
HMGN5
PDE4D
HAS2-AS1
HAS2
PTPRC
CPT1C
ARSJ
LINC00698
RP11-13A1.1
ZBED2
GSN
MYRIP
SSFA2
TESC
ADGRL3
DUSP6
PTPRK
GSAP
ANXA5
PTPN12
LAMA2
ST3GAL6
KRT23
TNC
PAM
LINC01515
ZNF772
FAM111B
ZNF615
ZNF419
ALDH1A1
AHR
MAGED1
RPP30
AF127936.9
NRIP1
ZNF493
DCLK1
KRTAP3-1
NR2F1-AS1
