# Supplementary material for: FOXO1 links KRAS G12D and G12V alleles to glutamine and nitrogen metabolism in colorectal cancer
Source: EMBO Rep. 2025 Nov 20;27(1):142–62. doi: 10.1038/s44319-025-00641-z (PMC12795846; doi:10.1038/s44319-025-00641-z)
Supplement: Supplementary file 8 — Appendix Figures Source Data [file 44319_2025_641_MOESM8_ESM.zip › FigS1.zip!/FigS1/FigS1F-J/MATLAB_KRAS_SW48/2024_fdr005_fc1topgenes_V/vulcano.pdf]

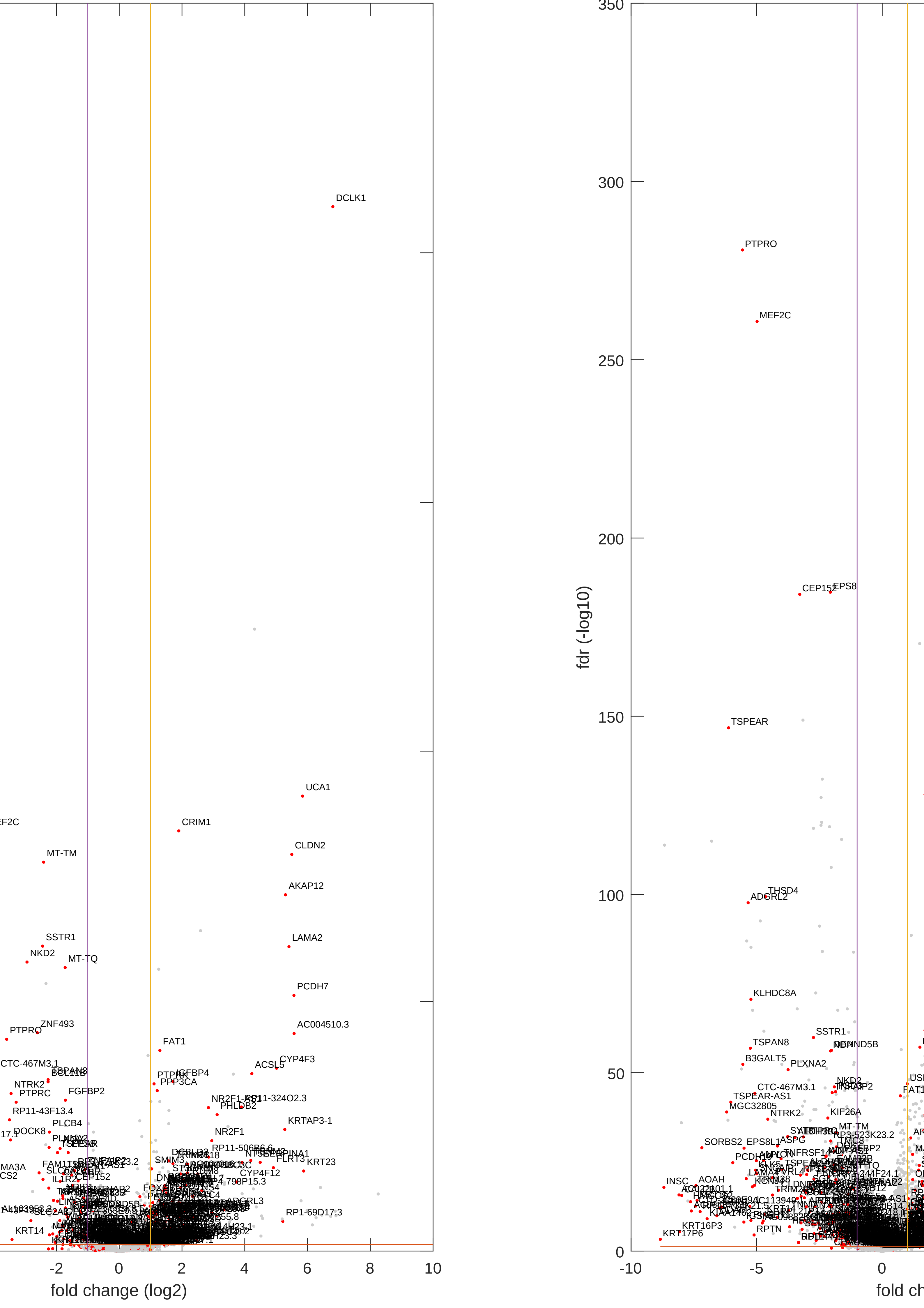

DCLK1
UCA1
CRIM1
CLDN2
MT-TM
AKAP12
SSTR1
LAMA2
NKD2
MT-TQ
PCDH7
ZNF493
PTPRO
AC004510.3
FAT1
CYP4F3
ACSL5
IGFBP4
PPP3CA
FGFBP2
NR2F1-AS1
PHLDB2
RP11-43F13.4
KRTAP3-1
PLCB4
DOCK8
NR2F1
KRT23
CYP4F12
ADGRL3
RP1-69D17.3
KRT14
-2
0
2
4
6
8
10
fold change (log2)
350
300
250
200
150
100
50
0
fdr (-log10)
PTPRO
MEF2C
CEP152
EPS8
TSPEAR
THSD4
ADGRL2
KLHDC8A
SSTR1
TSPAN8
B3GALT5
PLXNA2
CTC-467M3.1
TSPEAR-AS1
MGC32805
NTRK2
KIF26A
MT-TM
SORBS2
EPS8L1
ASPG
INSC
AOAH
KRT16P3
KRT17P6
RPTN
-10
-5
0
