# Supplementary material for: FOXO1 links KRAS G12D and G12V alleles to glutamine and nitrogen metabolism in colorectal cancer
Source: EMBO Rep. 2025 Nov 20;27(1):142–62. doi: 10.1038/s44319-025-00641-z (PMC12795846; doi:10.1038/s44319-025-00641-z)
Supplement: Supplementary file 8 — Appendix Figures Source Data [file 44319_2025_641_MOESM8_ESM.zip › FigS1.zip!/FigS1/FigS1F-J/MATLAB_KRAS_SW48/2024_fdr005_fc1VD_diff_equal_fig1C/pca_biplot.pdf]

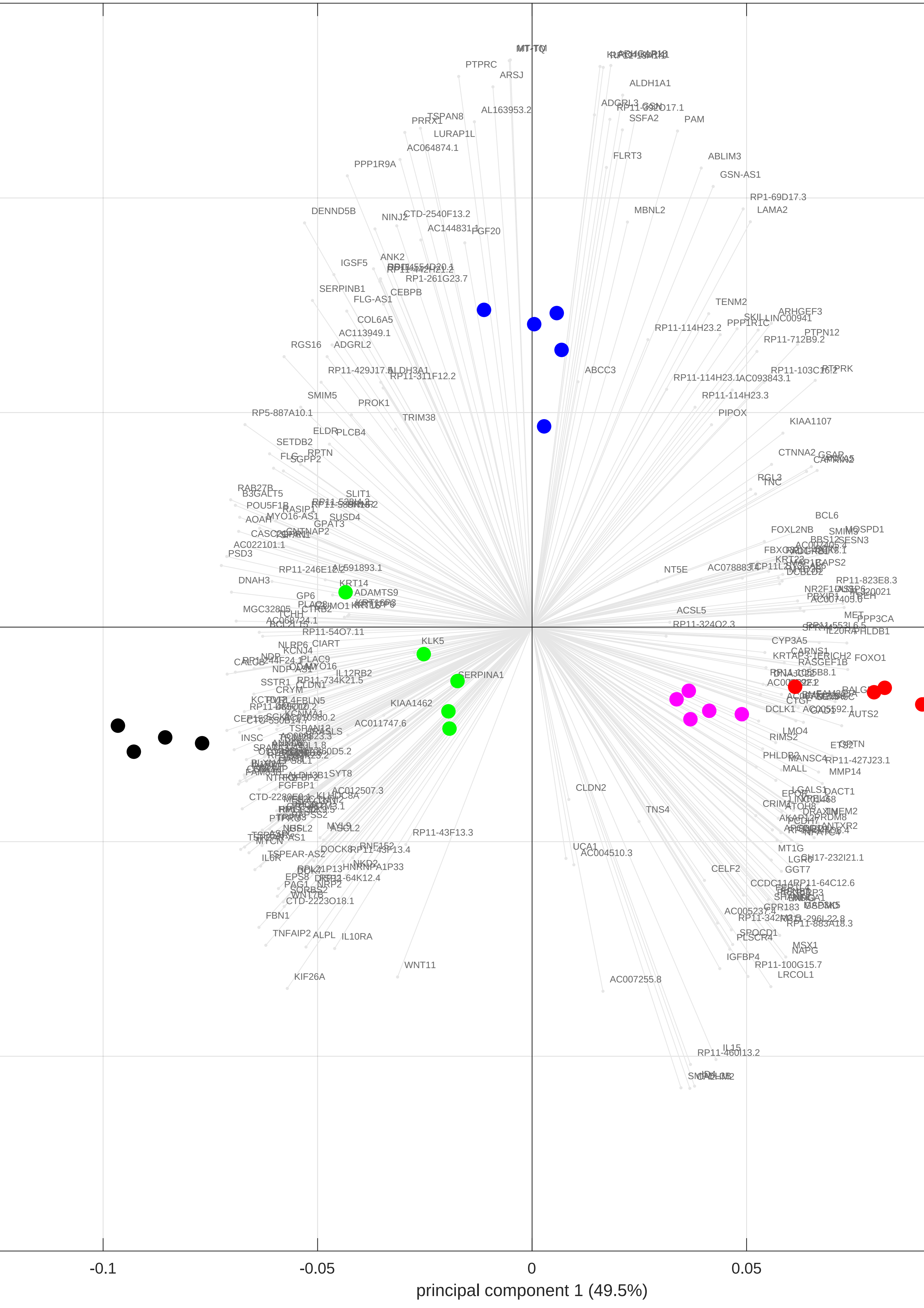

PTPRC
ARSJ
AL163953.2
ALDH1A1
RP11-392O17.1
SSFA2
PAM
PRRX1
TSPAN8
LURAP1L
AC064874.1
FLRT3
ABLIM3
GSN-AS1
PPP1R9A
RP1-69D17.3
LAMA2
DENND5B
NINJ2
CTD-2540F13.2
MBNL2
AC144831.1
FGF20
IGSF5
ANK2
RP1-261G23.7
SERPINB1
CEBPB
FLG-AS1
TENM2
ARHGEF3
COL6A5
AC113949.1
RP11-114H23.2
PPP1R1C
PTPN12
RP11-712B9.2
RGS16
ADGRL2
RP11-429J17.5
ALDH3A1
RP11-311F12.2
ABCC3
RP11-114H23.1
AC093843.1
RP11-103C16.2
PTPRK
SMIM5
PROK1
RP11-114H23.3
RP5-887A10.1
TRIM38
PIPOX
KIAA1107
SETDB2
RPTN
CTNNA2
B3GALT5
SLIT1
POU5F1B
SUSD4
AOAH
GPAT3
BCL6
FOXL2NB
SESN3
AC022101.1
PSD3
RP11-246E12.2
AL591893.1
NT5E
DCBLD2
DNAH3
KRT14
ADAMTS9
RP11-823E8.3
GP6
MGC32805
ACSL5
AC007405.6
RP11-324O2.3
PPP3CA
RP11-540T11
CIART
KCNJ4
CYP3A5
KLK5
PLAC9
FOXO1
RASGEF1B
IL12RB2
RP11-734K21.5
SERPINA1
SSTR1
CRYM
CLDN1
KIAA1462
DCLK1
AC005592.1
AUTS2
INSC
AC011747.6
LMO4
RIMS2
ETS2
OPTN
PHLDB2
RP11-427J23.1
MALL
MMP14
SYT8
FGFBP1
CLDN2
AC012507.3
LGALS1
DACT1
TNS4
RP11-43F13.3
TMEM2
UCA1
AC004510.3
DOCK8
RP11-43F13.4
RP11-64K12.4
GGT7
CELF2
RP11-64C12.6
WNT7B
CTD-2223O18.1
FBN1
RP11-883A18.3
TNFAIP2
ALPL
IL10RA
SPOCD1
PLSCR4
IGFBP4
RP11-100G15.7
LRCOL1
WNT11
KIF26A
AC007255.8
IL15
RP11-460I13.2
-0.1
-0.05
0
0.05
principal component 1 (49.5%)
