# Supplementary material for: FOXO1 links KRAS G12D and G12V alleles to glutamine and nitrogen metabolism in colorectal cancer
Source: EMBO Rep. 2025 Nov 20;27(1):142–62. doi: 10.1038/s44319-025-00641-z (PMC12795846; doi:10.1038/s44319-025-00641-z)
Supplement: Supplementary file 8 — Appendix Figures Source Data [file 44319_2025_641_MOESM8_ESM.zip › FigS1.zip!/FigS1/FigS1F-J/MATLAB_KRAS_SW48/2024_fdr005_fc1VD_UP_orangecluster/pca_biplot.pdf]

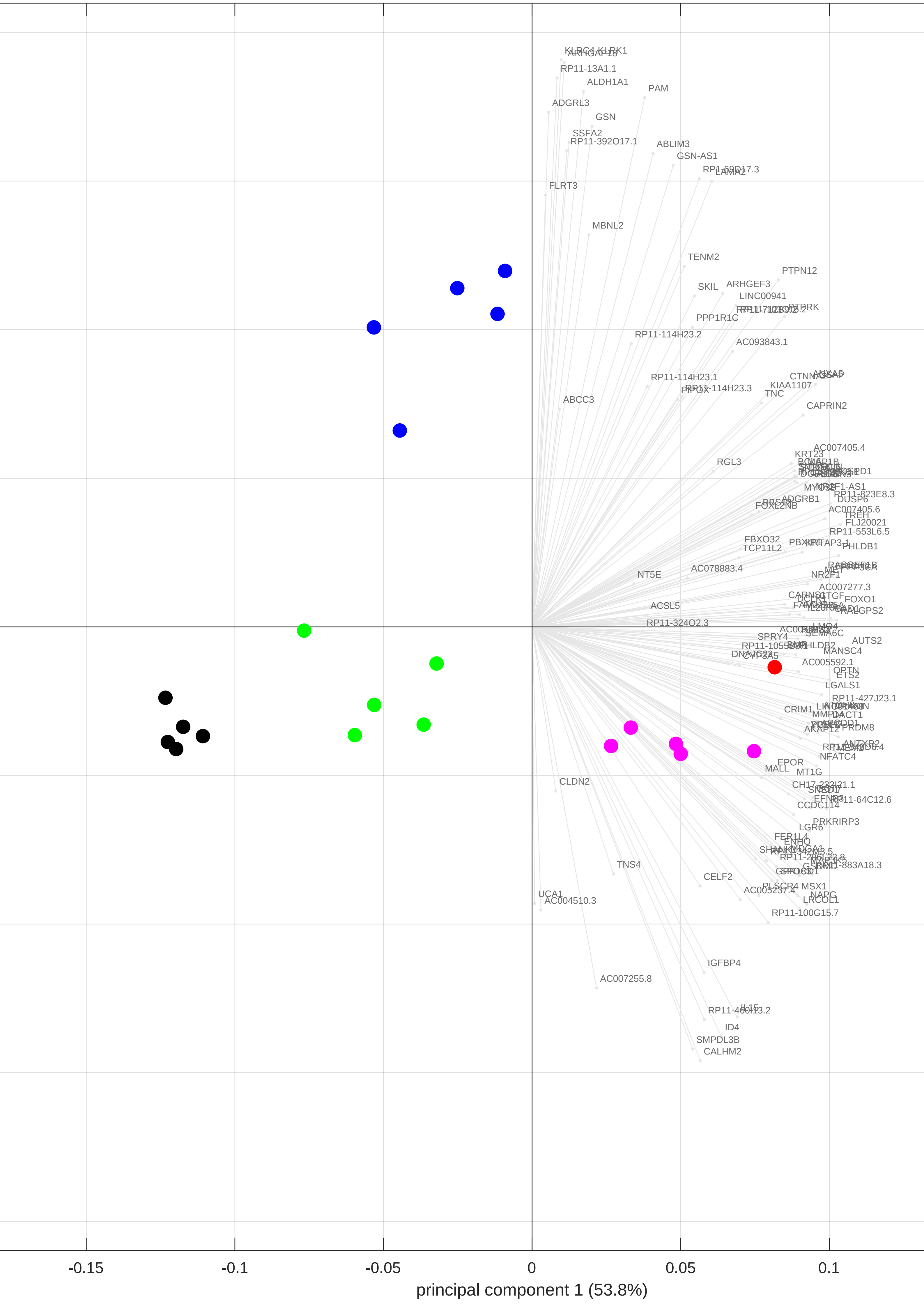
PAM
RP11-13A1.1
ALDH1A1
ADGRL3
GSN
SSFA2
RP11-392O17.1
ABLIM3
GSN-AS1
FLRT3
MBNL2
TENM2
PTPN12
SKIL
ARHGEF3
LINC00941
PPP1R1C
RP11-114H23.2
AC093843.1
RP11-114H23.1
KIAA1107
TNC
CAPRIN2
ABCC3
AC007405.4
KRT23
RGL3
ADGRB1
DUSP6
AC007405.6
TREH
FLJ20021
RP11-553L6.5
FBXO32
TCP11L2
PHLDB1
NT5E
AC078883.4
NR2F1
AC007277.3
FOXO1
ACSL5
RALGPS2
RP11-324O2.3
SPRY4
AUTS2
MANSC4
AC005592.1
OPTN
ETS2
LGALS1
RP11-427J23.1
CRIM1
PRDM8
NFATC4
EPOR
MALL
MT1G
CLDN2
CCDC114
PRKRIRP3
LGR6
TNS4
CELF2
MSX1
LRCOL1
RP11-100G15.7
UCA1
AC004510.3
IGFBP4
AC007255.8
ID4
SMPDL3B
CALHM2
-0.15
-0.1
-0.05
0
0.05
0.1
principal component 1 (53.8%)
