# Supplementary material for: FOXO1 links KRAS G12D and G12V alleles to glutamine and nitrogen metabolism in colorectal cancer
Source: EMBO Rep. 2025 Nov 20;27(1):142–62. doi: 10.1038/s44319-025-00641-z (PMC12795846; doi:10.1038/s44319-025-00641-z)
Supplement: Supplementary file 8 — Appendix Figures Source Data [file 44319_2025_641_MOESM8_ESM.zip › FigS1.zip!/FigS1/FigS1F-J/MATLAB_KRAS_SW48/2024_fdr005_fc1VD_UP_orangecluster/vulcano.pdf]

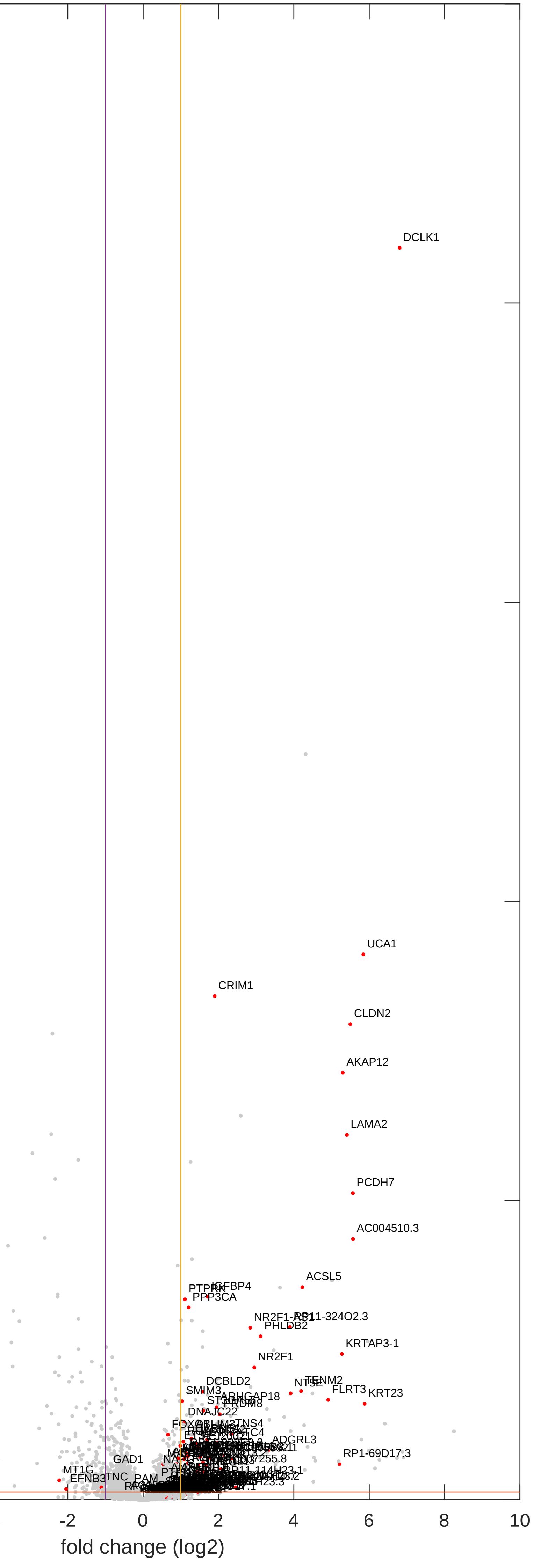
DCLK1
UCA1
CRIM1
CLDN2
AKAP12
LAMA2
PCDH7
AC004510.3
ACSL5
PTPRK
IGFBP4
PPP3CA
NR2F1-AS1
RP11-324O2.3
PHLDB2
KRTAP3-1
NR2F1
DCBLD2
TENM2
NT5E
FLRT3
KRT23
SMIM3
ARHGAP18
PRDM8
DNAJC22
ADGRL3
RP1-69D17.3
MT1G
EFNB3
TNC
GAD1
PAM
-2
0
2
4
6
8
10
fold change (log2)
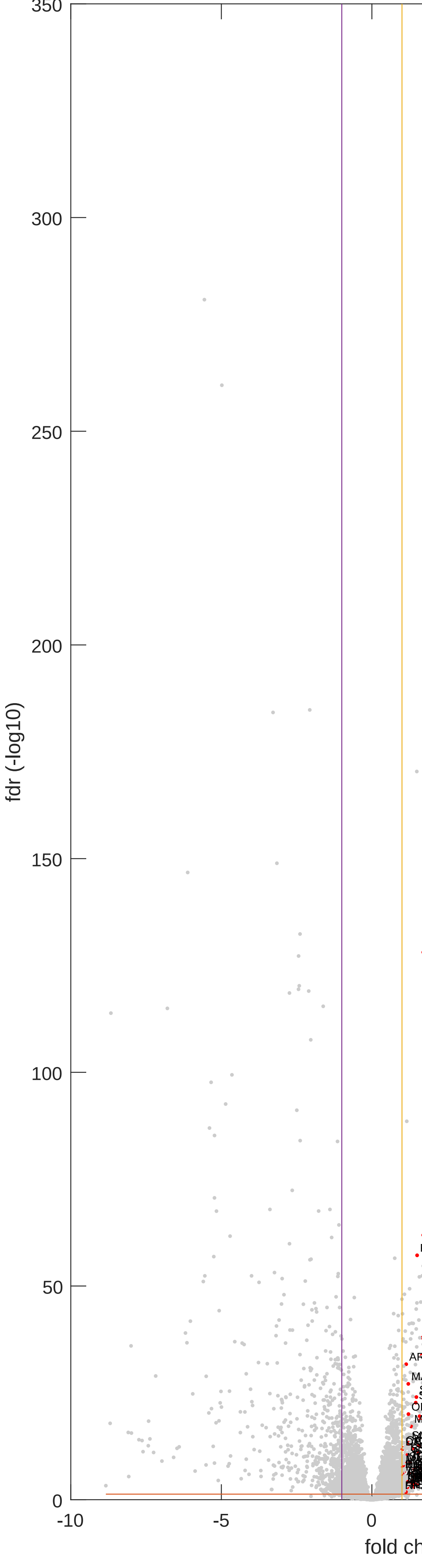
350
300
250
200
150
100
50
0
fdr (-log10)
-10
-5
0
